# Effectiveness over time of a primary series of the original monovalent COVID-19 vaccines in adults in the United States

**DOI:** 10.1371/journal.pone.0320434

**Published:** 2025-05-06

**Authors:** J. Bradley Layton, Patricia C. Lloyd, Lauren S. Peetluk, Yixin Jiao, Djeneba Audrey Djibo, Joann F. Gruber, Jie Deng, Christine Bui, An-Chi Lo, Rachel P. Ogilvie, Ron Parambi, Michael Miller, Jennifer Song, Lisa B. Weatherby, Sylvia Cho, Hui Lee Wong, Tainya C. Clarke, Jessica Rose Hervol, Dóra Illei, Elizabeth J. Bell, Grace Wenya Yang, John D. Seeger, Michael Wernecke, Morgan M. Richey, Richard A. Forshee, Steven A. Anderson, Yoganand Chillarige, Cheryl N. McMahill-Walraven, Kandace L. Amend, Mary S. Anthony, Azadeh Shoaibi

**Affiliations:** 1 RTI Health Solutions, Research Triangle Park, North Carolina, United States of America; 2 Center for Biologics Evaluation and Research, US Food and Drug Administration, Silver Spring, Maryland, United States of America; 3 Optum Epidemiology, Boston, Massachusetts, United States of America; 4 Acumen LLC, Burlingame, California, United States of America; 5 Safety Surveillance & Collaboration, CVS Health, Blue Bell, Pennsylvania, United States of America; 6 RTI International, Washington, District of Columbia, United States of America; 7 OptumServe, Falls Church, Virginia, United States of America; Gabriele d'Annunzio University of Chieti and Pescara: Universita degli Studi Gabriele d'Annunzio Chieti Pescara, ITALY

## Abstract

With data from 2 US claims databases (Optum, CVS Health) supplemented with Immunization Information System COVID-19 vaccine records, we evaluated overall and time-specific vaccine effectiveness (VE) of an initial primary series for 3 monovalent COVID-19 vaccines—BNT162b2, mRNA-1273, and JNJ-7836735—in adults (18-64 years). Vaccinated individuals were matched to unvaccinated comparators, and we estimated VE against any medically diagnosed COVID-19 and hospital/emergency department (ED)-diagnosed COVID-19. Additionally, we estimated VE by era of predominant variants, in subgroups, and compared across vaccine brands. The cohorts consisted of 341,097 (Optum) and 1,151,775 (CVS Health) matched pairs for BNT162b2; 201,604 (Optum) and 651,545 (CVS Health) for mRNA-1273; and 49,285 (Optum) and 149,813 (CVS Health) for JNJ-7836735. The study period began 11 December 2020 (date of first COVID-19 vaccine availability in the US) and ended 15 January 2022 in Optum and 31 March 2022 in CVS Health. Summary VE estimates from meta-analysis against hospital/ED-diagnosed COVID-19 were: BNT162b2, 77% (95% CI, 76%-78%); mRNA-1273, 84% (95% CI, 83%-85%), JNJ-7836735 66% (95% CI, 63%-68%). VE estimates were higher for hospital/ED-diagnosed COVID-19 than for medically diagnosed COVID-19, and VE estimates were highest in adults receiving mRNA-1273 for both outcomes. VE was sustained for approximately 7 months for medically diagnosed and up to 9 months for hospital/ED-diagnosed COVID-19. VE differed by brand and variant era. Ongoing real-world surveillance of COVID-19 vaccines using robust data sources and methodology is needed as new variants and recommendations for updated vaccines have evolved.

## Introduction

Vaccines for the prevention of coronavirus disease 2019 (COVID-19) became available in the United States (US) through either emergency use authorization or licensure by the US Food and Drug Administration (FDA). This study evaluated the initial, monovalent primary series of the first 3 authorized vaccines in the US—BNT162b2 (Pfizer-BioNTech’s messenger ribonucleic acid [mRNA] COVID-19 vaccine, Comirnaty®), mRNA-1273 (Moderna’s mRNA COVID-19 vaccine, Spikevax®), and JNJ-7836735 (Janssen Pharmaceutical Company’s adenovirus COVID-19 vaccine). These vaccines demonstrated efficacy in preventing various COVID-19 outcomes, including symptomatic infection [1–3], severe COVID-19 [1–4], and death due to COVID-19 [4] in clinical trials before authorization.

Many early, real-world US studies affirmed the real-world effectiveness of COVID-19 vaccines in preventing severe disease [5–12], though some suggested that vaccine effectiveness (VE) waned over time in fully vaccinated individuals [3,7,13–15]. However, some studies demonstrated only minimal or no meaningful decreases in VE estimates over time against more severe COVID-19 or COVID-19-related hospitalization [5–7,9,16]. One systematic review analyzing global real-world vaccine effectiveness studies or randomized controlled trials found that for severe COVID-19, VE or efficacy decreased by an average of 10 percentage points between 1 and 6 months after the final dose across all vaccine types analyzed, with most studies showing effectiveness against severe disease remaining above 70% over time [17]. In the context of rapidly evolving pandemic conditions, real-world studies on the duration of vaccines’ effectiveness may be challenging. Approaches that compare individuals with different lengths of follow-up may be subject to selection bias and time-related biases (e.g., immortal person-time bias) [18]. Changes in the predominant circulating viral variants further complicate the question of waning effectiveness over time. Specifically, the emergence of Delta and Omicron variants, which emerged several months after vaccination rollout, resulted in large increases in COVID-19 cases among unvaccinated and vaccinated individuals in the US [19]. FDA continues to monitor the safety and effectiveness of COVID-19 vaccines using real-world evidence through the Biologics Effectiveness and Safety (BEST) Initiative. This study, which is part of the BEST Initiative, evaluated the potential for changing effectiveness of the original complete COVID-19 primary series vaccine over time using rich real-world data and robust methodology. Authorizations and recommendations for COVID-19 vaccine dosing have changed over time, but during the study period, a “complete primary series” for the general population of adults was considered 2 doses of BNT162b2 21 days apart, 2 doses of mRNA-1273 28 days apart, or 1 dose of JNJ-7836735.

## Materials and methods

### Data source and population

This cohort study of adults aged 18-64 years was conducted using 2 US health insurance claims data sources (Optum pre-adjudicated commercial claims; CVS Health adjudicated commercial claims) supplemented with vaccination records from US Immunization Information Systems (IISs) (S1 Table in S1 Text) [20]. The data sources contain enrollment information, dispensed prescription drug claims, and hospital, physician, and healthcare professional health insurance claims for individuals with commercial insurance from throughout the US. The study period began on 11 December 2020 (the date COVID-19 vaccines were first available in the US) and ended on 15 January 2022 in Optum and 31 March 2022 in CVS Health (end of complete data at the time of analysis). The date of data access for study purposes was 28 April 2022 for Optum and 28 August 2022 for CVS Health.

Vaccinated individuals were identified at the first record of a COVID-19 vaccination during the study period (Time 0), and unvaccinated individuals were matched by calendar date, US county of residence, age (5-year increments), sex, immunocompromised status, pregnancy status, history of COVID-19 diagnosis, influenza vaccination, and presence of at least 1 of the conditions identified by CDC as increasing individuals’ risk of severe COVID-19 [21]. The calendar date of the vaccinated individual was set at Time 0 for the matched unvaccinated comparator.

Vaccinated and matched unvaccinated individuals were required to meet the following eligibility criteria on or before Time 0: have continuous medical and pharmacy coverage in the database before Time 0 for at least 365 days prior to Time 0 and beginning on or earlier than 11 December 2020 (the day COVID-19 vaccines were initially authorized in the US); be within the indicated/authorized age range of the vaccine; and reside within the catchment area of the combined claims and IIS data. To align vaccinated and unvaccinated individuals, all eligible individuals were required to be free of any of the following conditions in the specified time periods before Time 0, given that these could reduce the probability of vaccination: monoclonal antibody or convalescent plasma treatment (90 days); COVID-19 diagnosis (30 days); fever, nausea/vomiting, rash diagnosis (3 days); hospitalization or emergency department (ED) visit (3 days); or be hospitalized or a long-term care resident (on Time 0) [22].

### Exposure assessment

Vaccine doses were identified in insurance claims and IIS databases using procedure billing codes for vaccine administration, National Drug Codes for vaccine products, or IIS administration records [23]. The dose number was inferred from the order of observed doses within an individual’s record—thus continuous enrollment for the entire period since the introduction of vaccines was required. If a vaccine dose of the same brand or a dose record which did not specify the vaccine brand occurred within 3 days following another dose, the second record was considered a duplicate and was excluded; if a dose for a different brand was received within 3 days, the brand of the first dose was considered unknown and both dose records were excluded.

**Fig 1:**
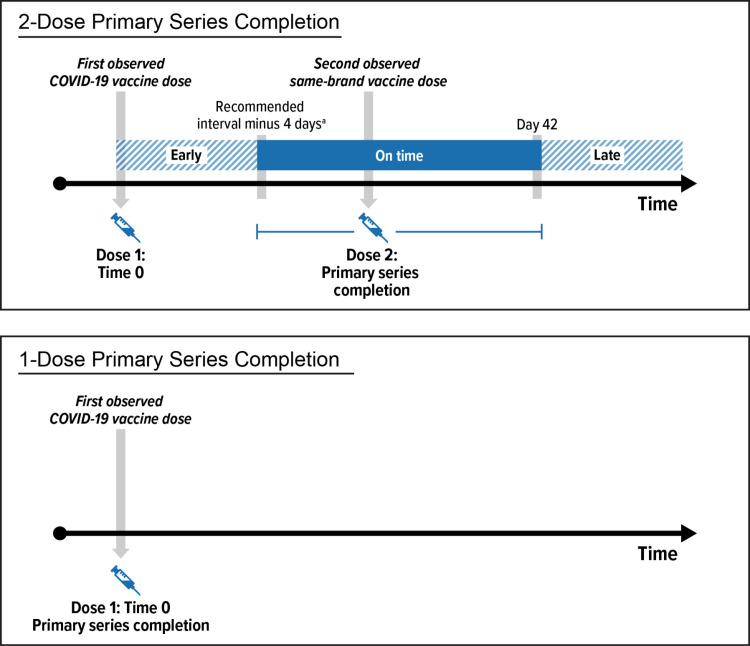
Patterns of Primary Series Completion for 2-Dose and 1-Dose COVID-19 Vaccines. COVID-19 =  coronavirus disease 2019, ^a^ Day 17 for BNT162b2, day 24 for mRNA-1273.

All subsequent COVID-19 vaccines received by an individual after Time 0 were used to evaluate continuing adherence to the primary series (Fig 1). Individuals were censored if they deviated from their vaccination strategy. For vaccines with 2-dose primary series, Dose 2 needed to be received no more than 4 days before the recommended interval and no more than 42 days after Dose 1 (S2 Table in S1 Text). Unvaccinated individuals who received a dose were censored from the unvaccinated group, and they could be considered for inclusion in the vaccinated group on the date of their vaccine receipt (thus individuals may be included in both exposure groups). A secondary analysis evaluated receipt of only a single dose of the 2-dose mRNA vaccine primary series (S1 Supplemental methods in S1 Text).

### Outcome assessment

Individuals were followed from Time 0 until the occurrence of an outcome of interest, or were censored at the first of the following: last day of the study period; health plan disenrollment; relocation outside of the claims and IIS data catchment area; death; deviation from receiving the primary series (i.e., receipt of Dose 2 too early, failure to receive Dose 2 on time, receiving a booster/additional dose) for the vaccinated, or receipt of any COVID-19 vaccine for the unvaccinated.

Two nested, non-mutually exclusive, COVID-19 outcomes were evaluated separately: (1) medically diagnosed COVID-19, identified as a recorded COVID-19 diagnosis from hospital, ED, outpatient, or physician encounters; and (2) hospital/ED-diagnosed COVID-19. Recorded COVID-19 diagnosis codes (ICD-10-CM U07.1) were identified in claims in any diagnosis field coding position. For each outcome, the date of the first observed qualifying diagnosis was assigned as the outcome date.

### Covariates

**Fig 2:**
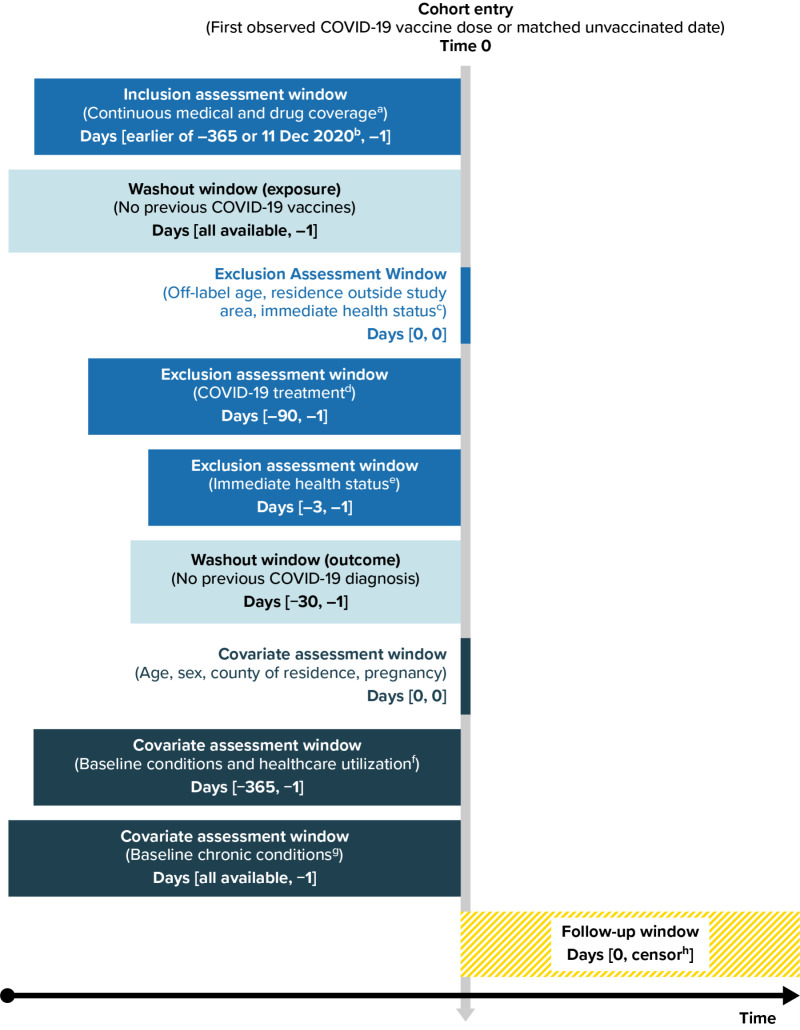
Schematic for Assessing Eligibility, Covariates, and COVID-19 Outcomes Among Vaccinated Individuals and Matched Unvaccinated Comparators. COVID-19 = coronavirus disease 2019; ED = emergency department; LTC = long-term care. Note: Use of “all available” data indicates that the entire duration of an individual’s available continuous enrollment information before Time 0, back to the beginning of data availability (1 December 2017), was used; the duration of available data was at least 365 days, but may vary for each individual. ^a^ Gaps in medical and pharmacy coverage < 32 days permitted. ^b^ Date of first COVID-19 vaccine authorization in the US. ^c^ Hospitalization or long-term care residence at Time 0. ^d^ COVID-19 monoclonal antibodies or convalescent plasma. ^e^ Diagnoses of general acute symptoms (fever, nausea/vomiting, rash) and healthcare utilization (hospitalization, ED visit) serving as an indicator of health status at the time of vaccination. ^f^ Number of hospitalizations, number of ED visits, skilled nursing facility stay, influenza vaccination, pneumococcal vaccination, encounter for cancer screening, eye examination, colonoscopy, bone mineral density test, well-check/well-child preventive healthcare visit, arthritis, lipid abnormality, ambulance use/life support service, weakness, pregnancy completion before Time 0. ^g^ Autoimmune disorders, cancer, chronic kidney disease or renal disease, chronic liver disease, chronic lung diseases (e.g., asthma, chronic obstructive pulmonary disease [COPD], cystic fibrosis, pulmonary embolism), dementia or other neurological conditions, diabetes mellitus type 1 or 2, Down syndrome, heart conditions (e.g., heart failure, coronary artery disease, arrhythmias), hypertension, immunocompromised state, mental health conditions, obese or severely obese, sickle cell disease or thalassemia, stroke or cerebrovascular disease, tuberculosis, COVID-19 laboratory test performed (binary indicator of any test performed or none), COVID-19 diagnoses. ^h^ End of study period, end of continuous health plan enrollment, relocation out of study area, deviation from the categorized vaccine exposure status.

Descriptive characteristics were measured on or before Time 0 (Fig 2) using enrollment, diagnosis, procedure, and pharmacy data [24]. Covariates in both data sources included demographics, comorbidities, frailty indicators, healthcare utilization, and conditions potentially increasing risk of severe COVID-19 [21] (complete list shown in Fig 2 and Supporting Information S3-S5 Table in S1 Text).

### Statistical analysis

Analyses were performed separately by data source and vaccine brand. The distribution of characteristics by vaccination group were described with means, standard deviations (SDs), medians, and first and third quartiles (Q1, Q3) for continuous variables, and counts and proportions for categorical variables. Covariate balance between vaccination groups was evaluated with absolute standardized differences [25].

In addition to individual matching on select characteristics, propensity score weighting was used to address confounding. Propensity scores were estimated with multivariable logistic regression models including all prespecified covariates and matching factors (S1 Fig in S1 Text). Stabilized inverse probability of treatment (sIPT) weights were estimated from propensity scores with truncation below the 1st percentile and above the 99th percentile of the propensity score distribution.

The cumulative incidence of each COVID-19 outcome was estimated in the sIPT-weighted vaccine exposure groups as 1 minus the Kaplan-Meier estimator [26]. Cumulative incidence of COVID-19 outcomes in the first 14 days of follow-up was evaluated as a negative control outcome because vaccines are not expected to produce an immune response until 10-14 days after vaccination [27, 28]. Hazard ratios (HRs) for the association of vaccination status with COVID-19 outcomes were estimated using sIPT-weighted Cox proportional hazards models; 95% confidence intervals (CIs) were estimated with robust sandwich variance estimators [29]. Overall VE was estimated as 1 minus the HR. Time-specific VEs were estimated using daily cumulative incidence estimates in both treatment groups to estimate daily risk ratios (RRs) from which VEs were estimated as 1 minus the RR; time-specific risk differences (RDs) were estimated by subtracting daily cumulative incidence estimates. Nonparametric bootstrapping was used to estimate 95% CIs for time-specific VEs and RDs.

Meta-analyses across data sources were performed with fixed-effects meta-analysis methods [30] for the overall, subgroup, and variant era–specific VE estimates. Statistical evidence of heterogeneity between data source estimates was evaluated by obtaining log-transformed HR estimates and their standard errors, with *p* values less than 0.05 indicating evidence of statistical heterogeneity.

Subgroup analyses for the overall VE estimation were performed by age, immunocompromised status, and history of COVID-19 diagnosis. Analyses by variant era (pre-Delta era, 11 December 2020 through 31 May 2021; Delta era, 1 June 2021 through 24 December 2021; Omicron era, 25 December 2021 through the end of data availability [31]) were restricted to individuals with Time 0 within the era, and follow-up was censored on the last day of the era. Two sensitivity analyses evaluated the potential selection bias introduced by censoring for receipt of a censoring dose (e.g., individuals in the unvaccinated group receiving any vaccine, or individuals in the vaccinated group receiving Dose 2 too early, Dose 2 of a different brand, or a third dose): (1) delay censoring for 7 days after receipt of a censoring vaccine dose, as there would not be an expected effect during this time; and (2) apply inverse probability of censoring weights.

Quantitative bias analyses (QBA) [32, 33] estimated the impact of potential exposure misclassification because of missing vaccine records by estimating corrected HRs accounting for a range of vaccine exposure sensitivities (S2 Supplemental methods in S1 Text).

A secondary analysis evaluated the relative VE (RVE) of receiving a complete primary series of different COVID-19 vaccine brands. For each pairwise comparison (i.e., mRNA-1273 vs. BNT162b2; JNJ-7836735 vs. BNT162b2; JNJ-7836735 vs. mRNA-1273), the study period was restricted to calendar time periods when both vaccine brands in that comparison were authorized. To account for variation in local COVID-19 burden between the 2 exposure groups, vaccinated individuals in both exposure groups were exact-matched 1:1 without replacement on calendar week, age, sex, and US county of residence. RVE was estimated as 1 minus the HR for each comparison.

Statistical analyses were performed with SAS Version 9.4 (SAS Institute Inc., Cary, NC) and R 4.1.2 (R Foundation for Statistical Computing, Vienna, Austria). This surveillance activity was conducted as part of the FDA public health surveillance mandate. As a public health surveillance activity conducted under the direction of a US public health authority, this activity did not constitute research and was not subject to Institutional Review Board oversight according to US regulations 45 CFR Part 46.102(k) and 46.102(l)(2). Individual-level consent was not required for this analysis of secondary healthcare data. Individual participants could not be identified during or after data collection by the study authors. The study protocol was publicly posted on the BEST Initiative webpage [24].

## Results

### Descriptive analyses

In Optum, we identified 612,125 individuals who received an eligible first COVID-19 vaccine dose during the study period. After matching, the analytic cohorts consisted of 341,097 in each group for BNT162b2, 201,604 in each group for mRNA-1273, and 49,285 in each group for JNJ-7836735. In CVS Health, we identified 1,979,109 eligible individuals receiving a first COVID-19 vaccine dose during the study period. After matching, the analytic cohorts consisted of 1,151,775 in each group for BNT162b2, 651,545 in each group for mRNA-1273, and 149,813 in each group for JNJ-7836735. The length of follow-up varied across vaccine brands and outcome analyses, but in Optum, median follow-up ranged from 232 to 248 days for the vaccinated groups, and 88 to 114 days for the unvaccinated; in CVS Health, median follow-up ranged from 244 to 258 days in the vaccinated, and from 102 to 129 days in the unvaccinated.

Selected characteristics of individuals who received a COVID-19 vaccine dose and matched unvaccinated comparators are shown in Table 1 (complete details in S3 Table through S5 Table in S1 Text). Across both data sources, the mean age was approximately 42 to 43 years for each vaccine-specific cohort. The BNT162b2 and mRNA-1273 matched cohorts were slightly more female (51%-52%), but the JNJ-7836735 matched cohort was slightly more male (56%). A very small proportion of the cohorts were pregnant at the time of vaccination (approximately ≤  1%), and approximately 4% of each cohort were immunocompromised. Vaccine and comparator groups were matched exactly on age, sex, geography, pregnancy, COVID-19 history, and immunocompromised status, thus these characteristics were all perfectly balanced in the matched groups. Additionally, all other measured characteristics (i.e., healthcare utilization, comorbidities, frailty markers) were all well balanced between treatment groups as indicated by absolute standardized differences close to 0 (S3 Table through S5 Table in S1 Text). The substantial overlap of propensity score distributions for all cohorts indicated a high degree of exchangeability between vaccinated and unvaccinated groups for all measured covariates (S1 Fig in S1 Text).

**Table 1:** Selected Characteristics of Adults Aged 18-64 Years Vaccinated with a COVID-19 Vaccine and Matched Unvaccinated Comparators.

Characteristic	BNT162b2		mRNA-1273		JNJ-7836735	
	Vaccinated	Matched, unvaccinated comparators	Vaccinated	Matched, unvaccinated comparators	Vaccinated	Matched, unvaccinated comparators
**Optum**	**N = 341,097**	**N = 341,097**	**N = 201,604**	**N = 201,604**	**N = 49,285**	**N = 49,285**
*Age, years*						
Median (Q1, Q3)	42 (32, 53)	42 (32, 53)	44 (33, 54)	44 (33, 54)	44 (33, 54)	44 (33, 54)
Mean (SD)	42.03 (12.95)	42.02 (12.95)	43.23 (12.95)	43.19 (12.97)	42.91 (12.88)	42.89 (12.87)
*Sex, N (%)*						
Female	175,029 (51.31%)	175,029 (51.31%)	103,947 (51.56%)	103,947 (51.56%)	21,443 (43.51%)	21,443 (43.51%)
Male	166,068 (48.69%)	166,068 (48.69%)	97,657 (48.44%)	97,657 (48.44%)	27,842 (56.49%)	27,842 (56.49%)
*Region, N (%)*						
Midwest	144,657 (42.41%)	144,657 (42.41%)	85,564 (42.44%)	85,564 (42.44%)	22,568 (45.79%)	22,568 (45.79%)
Northeast	40,009 (11.73%)	40,009 (11.73%)	26,580 (13.18%)	26,580 (13.18%)	5,838 (11.85%)	5,838 (11.85%)
South	86,610 (25.39%)	86,610 (25.39%)	47,138 (23.38%)	47,138 (23.38%)	10,032 (20.36%)	10,032 (20.36%)
West	69,821 (20.47%)	69,821 (20.47%)	42,322 (20.99%)	42,322 (20.99%)	10,847 (22.01%)	10,847 (22.01%)
Pregnant at Time 0, N (%)	1,837 (1.05%)	1,837 (1.05%)	759 (0.73%)	759 (0.73%)	72 (0.34%)	72 (0.34%)
Immunocompromised	15,135 (4.44%)	15,135 (4.44%)	9,000 (4.46%)	9,000 (4.46%)	1,865 (3.78%)	1,865 (3.78%)
**CVS Health**	**N = 1,151,775**	**N = 1,151,775**	**N = 651,545**	**N = 651,545**	**N = 149,813**	**N = 149,813**
*Age, years*						
Median (Q1, Q3)	42 (31, 53)	42 (31, 53)	44 (33, 55)	44 (32, 55)	44 (32, 54)	44 (32, 54)
Mean (SD)	41.92 (13.22)	41.91 (13.23)	43.37 (13.4)	43.33 (13.42)	42.98 (13.31)	42.97 (13.32)
*Sex, N (%)*						
Female	615,551 (53.44%)	615,551 (53.44%)	338,084 (51.89%)	338,084 (51.89%)	67,116 (44.80%)	67,116 (44.80%)
Male	536,224 (46.56%)	536,224 (46.56%)	313,461 (48.11%)	313,461 (48.11%)	82,697 (55.20%)	82,697 (55.20%)
*Region, N (%)*						
Midwest	206,280 (17.91%)	206,280 (17.91%)	114,493 (17.57%)	114,493 (17.57%)	28,431 (18.98%)	28,431 (18.98%)
Northeast	204,777 (17.78%)	204,777 (17.78%)	139,585 (21.42%)	139,585 (21.42%)	29,728 (19.84%)	29,728 (19.84%)
South	251,233 (21.81%)	251,233 (21.81%)	113,510 (17.42%)	113,510 (17.42%)	26,237 (17.51%)	26,237 (17.51%)
West	489,485 (42.50%)	489,485 (42.50%)	283,957 (43.58%)	283,957 (43.58%)	65,417 (43.67%)	65,417 (43.67%)
Pregnant at Time 0, N (%)	6,611 (0.57%)	6,611 (0.57%)	2,557 (0.39%)	2,557 (0.39%)	274 (0.18%)	274 (0.18%)
Immunocompromised	49,753 (4.32%)	49,753 (4.32%)	31,211 (4.79%)	31,211 (4.79%)	5,561 (3.71%)	5,561 (3.71%)

COVID-19 = coronavirus disease 2019; IQR = interquartile range; SD = standard deviation.

### Outcome analyses

In each cohort, incidence of medically diagnosed COVID-19 was much higher than the incidence of hospital/ED-diagnosed COVID-19. For instance, in Optum, the BNT162b2 vaccinated group had 11,399 medically diagnosed cases and 1,066 hospital/ED-diagnosed cases in nearly 70 million person-days of follow-up; in CVS Health, the BNT162b2 vaccinated group had 40,116 medically diagnosed cases and 4,496 hospital/ED-diagnosed cases in nearly 270 million person-days of follow-up (S6 Table in S1 Text).

#### Overall vaccine effectiveness.

**Fig 3:**
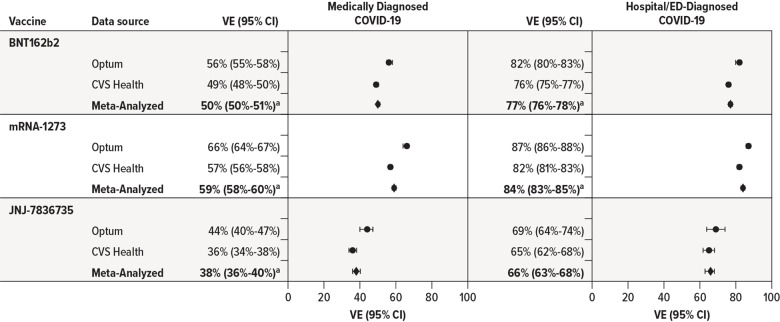
Estimated Effectiveness of Receiving a Complete Primary Series of COVID-19 Vaccines Compared With Being Unvaccinated in Adults Aged 18-64 Years. CI =  confidence interval; ED =  emergency department; VE =  vaccine effectiveness. ^a^ Indicates evidence of statistical heterogeneity between data source–specific estimates, *p* < 0.05.

The overall VE estimates are summarized in Fig 3 (complete details in S6 Table in S1 Text). Summary VE estimates from meta-analysis against medically diagnosed COVID-19 for receiving the complete primary series compared with being unvaccinated ranged from 38% to 59% (BNT162b2, 50% [95% CI, 50%-51%]; mRNA-1273, 59% [95% CI, 58%-60%]; JNJ-7836735, 38% [95% CI, 36%-40%]). Meta-analysis VE estimates against hospital/ED-diagnosed COVID-19 were higher, ranging from 66% to 84% (BNT162b2, 77% [95% CI, 76%-78%]; mRNA-1273, 84% [95% CI, 83%-85%]; JNJ-7836735, 66% [95% CI, 63%-68%]). For all outcomes and vaccines, Optum VE estimates were slightly higher than those in CVS Health. Because of the precision of the study estimates, most estimates of overall VE demonstrated evidence of statistical heterogeneity, but estimated magnitudes were similar clinically; therefore, summary VE estimates were reported for all estimates as summaries across both data sources, despite evidence of statistical heterogeneity.

In subgroup analyses, VE estimates for those with a previous COVID-19 diagnosis were generally lower than in those without a previous COVID-19 diagnosis for all brands and both outcomes in both data sources (S2 Fig in S1 Text); however, in the unvaccinated groups, COVID-19 incidence was generally lower among those with a previous COVID-19 diagnosis than among those without a previous diagnosis (S7 Table in S1 Text). Sensitivity analyses were mostly consistent with the primary analyses, but the IPC-weighted analyses resulted in slightly higher VE estimates for the medically diagnosed COVID-19 outcome (S3 Fig in S1 Text). QBA for potentially missing vaccine records suggest the observed overall VE estimates may underestimate the true VE by 2% to 14%, depending on the outcome, analysis, and data source (S8 Table in S1 Text).

#### Vaccine effectiveness over time.

To evaluate potential changing VE over time for the primary vaccinated versus unvaccinated comparison, the cumulative incidence of COVID-19 outcomes over time by vaccination status and vaccine brand was plotted (S4 Fig in S1 Text). Because of its introduction later in the study period, there was less available follow-up time for the JNJ-7836735 vaccine than the other available vaccines. For each COVID-19 vaccine comparison and outcome, rates were higher in the unvaccinated group throughout follow-up. Generally, differences in outcome rates between the 2 groups were initially small but then widened over time.

**Fig 4:**
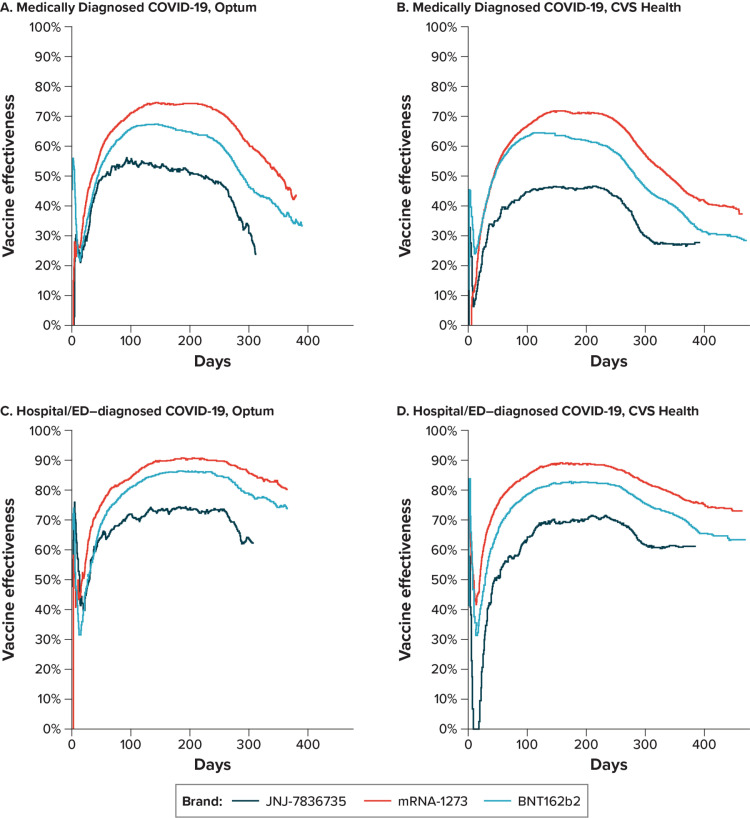
Estimated Effectiveness Over Time of Receiving a Complete Primary Series of COVID-19 Vaccines Compared With Being Unvaccinated in Adults Aged 18-64 Years. COVID-19 = coronavirus disease 2019; ED = emergency department.

Daily VE estimates throughout follow-up were estimated, and the VE estimates over time were plotted (Fig 4; VE and RD estimates and 95% CIs at specific timepoints are reported in S9 Table in S1 Text). Estimates fluctuated widely during the first week after Time 0 (as the early time periods have the smallest number of events, and estimates were highly subject to change), and negative control outcome analyses of the first 14 days revealed some small differences between vaccinated and unvaccinated groups, mainly in the first few days immediately after vaccination (S5 Fig in S1 Text). Following the initial few days after vaccination, all vaccines followed similar patterns: for medically diagnosed COVID-19, VE estimates increased over time until approximately 90 days and were sustained through approximately day 183, with evidence of waning afterward by day 270; for hospital/ED-diagnosed COVID-19, the pattern was very similar, but the VE estimates were higher throughout, and a smaller degree of waning was observed by day 270 (Fig 4, S9 Table in S1 Text). Despite evidence of waning of the VE estimate, absolute RD estimates showed increasing reductions in numbers of cases through at least day 270 after vaccination (S9 Table in S1 Text).

#### Vaccine effectiveness by variant era.

VE against both COVID-19 outcomes generally declined between the pre-Delta and Omicron eras (S6 Fig in S1 Text), although there was less available follow-up time during the Omicron era (e.g., only 20 days of follow-up in Optum occurred during the Omicron era), resulting in imprecise estimates. The change in VE between the pre-Delta and Delta eras was greatest for medically diagnosed COVID-19; the meta-analyzed VE of JNJ-7836735 declined by 12 percentage points, followed by BNT162b2 with a 3–percentage point decline. In contrast, the VE of mRNA-1273 increased by 4 percentage points between the pre-Delta and Delta period. Much larger decreases in VE were observed between the Delta and Omicron eras (S6 Fig in S1 Text). For hospital/ED-diagnosed COVID-19, a similar pattern was observed, with only modest changes between the pre-Delta and Delta eras, and more substantial decreases with the Omicron era.

#### Relative vaccine effectiveness.

When estimating the overall RVE for each vaccine brand during time periods when both brands were available, the summary VE estimates for mRNA-1273 demonstrated 16% (95% CI: 15%, 18%) to 19% (95% CI: 14%, 24%) higher effectiveness than BNT162b2 for medically diagnosed and hospital/ED-diagnosed COVID-19, respectively. The relative effectiveness of the 1-dose JNJ-7836735 series was lower than both 2-dose mRNA vaccine brands for both outcomes (S7 Fig in S1 Text).

#### Receiving only one dose.

For the analysis of receiving only a single dose of a 2-dose primary series compared with being unvaccinated, meta-analyzed VE estimates for hospital/ED-diagnosed COVID-19 were 57% (95% CI: 54%, 59%) and 69% (95% CI: 67%, 72%) for single doses of BNT162b2 and mRNA-1273, respectively (S8 Fig, S9 Fig in S1 Text). However, these VE estimates for both outcomes were more modest than those seen for the analyses of the complete 2-dose primary series.

## Discussion

In this large, real-world evaluation, receiving a complete primary series of COVID-19 vaccination was effective at preventing medically diagnosed COVID-19 and hospital/ED-diagnosed COVID-19 in adults aged 18 to 64 years, and that effectiveness was sustained for at least 7 months. The observed VE estimates were generally higher for hospital/ED-diagnosed COVID-19 than for any medically diagnosed COVID-19 and were highest in adults receiving the mRNA-1273 vaccine across all analyses. Results from both data sources suggested lower VE in adults receiving the JNJ-7836735 vaccine.

VE was also reduced during the Omicron era across all brands, although sample sizes were too small for the hospital/ED-diagnosed COVID-19 results to be meaningful. When evaluating VE by variant era, VE estimates were lower in the Omicron era than in the Delta or pre-Delta eras for all vaccines and both outcomes. Although waning of VE for the primary series and lower VE in later variant eras was observed, booster doses were recommended and have also been demonstrated to be effective [34].

The efficacy and effectiveness of the 3 COVID-19 vaccine brands available in the US has been the subject of a large volume of research over the past several years. Multiple real-world studies have also evaluated changes in VE over time using a variety of data sources and study designs [17], and real-world estimates of the vaccines’ effectiveness have varied substantially depending on geography, severity of disease, and the variant in circulation at the time of data collection [35]. However, several U.S. state-based cohort studies showed similar patterns of waning as this study. In these cohort studies, effectiveness of the primary vaccine series against hospitalization remained strong (above 85%) over calendar time [36], with one cohort study reporting effectiveness against hospitalization of approximately 89% for BNT162b2 and 94% effectiveness mRNA-1273 at 7 months after the first dose [37]. Another cohort study, which included individuals over age 12, found vaccine effectiveness against hospitalization for the BNT162b2 vaccine during the delta era was 93% at 6 months, though effectiveness against COVID-19 infection declined during that time [7]. Pooled VE estimates from meta-analyses of international real-world studies also demonstrated generally sustained VE against hospitalization over at least 6 months, but reductions in VE against documented infections during the Omicron era [38] and rapid waning of VE against symptomatic disease and laboratory-confirmed infection during the Delta and Omicron eras [39]. Additionally, the comparative effectiveness results we observed are generally consistent with other published studies, namely that the mRNA-based vaccines had higher VE than JNJ-7836735 [40, 41] and that, while VE estimates for both were high, mRNA-1273 had slightly higher VE than BNT162b2 [42–44]. While we did not specifically explore the potential underlying causes of the differences between vaccines’ VE estimates, differences between the vaccines’ humoral responses have been noted previously [45, 46].

While many previous studies used test-negative designs or cohort designs evaluating follow-up after receipt of a certain number of doses, our study used a cohort approach starting follow-up at the receipt of a first dose and defining adherence to a complete primary series; this avoided selection bias common to many studies comparing different lengths of exposure [47], and evaluated a clearly-defined vaccination strategy rather than discrete doses (matching the vaccination recommendations in place at the time). Our study utilized 2 US data sources, representing commercially insured adults from jurisdictions across the US with participating IISs who received COVID-19 vaccinations in various healthcare settings and geographic locations. Despite the different data sources, the overall results as well as many of subgroup results were consistent. To summarize the results from these 2 data sources, VE estimates from a meta-analysis are presented. The VE estimates and CIs are based on large sample sizes, and thus the estimates were very precise; therefore, statistical heterogeneity was observed in many of these results, based on the *I*^*2*^ statistic, which in some settings would prompt reconsideration of the appropriateness of meta-analysis. However, because our analytical approaches in each data source were identical and the populations were similar, we did not consider the observed differences in VE estimates to be clinically different (the direction and magnitude of the estimates were similar); thus, we considered meta-analysis to be a reasonable approach to summarizing, smoothing, and drawing inferences [48, 49].

The eligibility and matching criteria were designed to avoid selection bias by identifying vaccinated and unvaccinated individuals who were eligible for vaccination on each calendar day. Starting follow-up on Time 0 without considering future vaccination behaviors avoided immortal person-time bias by not using future information to define exposure status at baseline [18]. The daily VE estimates allowed for granular evaluation of changing VE over time.

Initial vaccination rollout focused on higher-risk individuals, but the exact prioritization procedures and groups varied by jurisdiction and changed over time. Because authorizations and recommendations for vaccination changed rapidly during the initial vaccine role out, defining time period- and geography-specific eligibility criteria for the entire study population was impractical; thus, we matched unvaccinated individuals to vaccinated on multiple indicators of eligibility status, including calendar date, immunocompromised status, pregnancy, comorbidities, and age; this matching approximated the rolling eligibility criteria by identifying unvaccinated individuals who were similar to vaccinated individuals on each calendar day in each US county of residence.

Despite the study’s matching on demographic and clinical characteristics and using propensity score weighting, residual confounding may remain. The generally increased VE estimates during the first 14 days of follow-up (i.e., the negative control analysis) suggested a potential difference between the exposure groups during a time when vaccines are assumed to have no biologic effect while the body mounts an immune response to a novel antigen. However, other studies [50] have demonstrated differential COVID-19 testing and diagnoses in the 3-4 days after Time 0, as recently vaccinated individuals may not seek COVID-19 testing, attributing symptoms to vaccine side effects. Additionally, diagnoses of COVID-19 recorded on the same day as vaccination may indicate recording of history of COVID-19 [51], and these Time 0 events may be more common with vaccine brands administered in healthcare practice settings, rather than pharmacies or mass vaccination centers where concurrent diagnoses cannot be recorded. However, longer-term differences in healthcare-seeking behavior or residual unmeasured confounding cannot be ruled out.

This study evaluated a complete primary series of a COVID-19 vaccine, i.e., 2 doses of BNT162b2 or mRNA-1273 with the correct spacing between doses (S2 Table in S1 Text) or a single dose of JNJ-7836735. During the study period, immunocompromised individuals were authorized to receive an additional dose of vaccine as part of the primary series; however, booster doses for immunocompetent individuals were also authorized during the same period. Local interpretations of immunocompromised status to determine additional dose eligibility may have varied across geography, over time, and by use case, making it difficult to differentiate those receiving a booster dose and immunocompromised individuals receiving an additional dose. Therefore, we considered additional doses combined with booster doses and treated them as censoring criteria in this study. We evaluated the effectiveness of booster/additional doses separately, and the results are presented elsewhere [34].

COVID-19 testing capacity and capability have changed rapidly throughout the course of the pandemic, and COVID-19 laboratory testing results were not widely available in the databases utilized for this study, so COVID-19 diagnoses rather than confirmed COVID-19 infections were used as outcomes. COVID-19 severity is not accurately represented in diagnoses [52], so we evaluated COVID-19 diagnoses overall in any medically attended setting, and separately in hospital/ED settings as a proxy for severity. However, as the pandemic has progressed, COVID-19–related hospitalizations may be the most relevant public health measure for contemporary surveillance and prevention efforts [52, 53]. We matched vaccination groups on calendar time and evaluated outcomes over calendar time to account for the potentially changing meaning of a COVID-19 diagnosis over time (e.g., care seeking for COVID-19 versus incidental findings).

This real-world study is subject to many limitations common to observational research using existing data sources (e.g., key study elements may be misclassified or missing, the observed VEs may be subject to confounding by unmeasured characteristics, and these results may not be generalizable to other populations). We used health insurance claims supplemented with IIS encounters to identify vaccination status, substantially increasing vaccination capture compared to either data source alone [20]. However, some vaccines may still be missed, resulting in differential misclassification of exposure. We expect that some truly vaccinated persons may have been classified as unvaccinated due to missing or unidentifiable records, recording errors, or other technical challenges that resulted in incomplete recording of information during the pandemic response. Given this potential for less than 100% sensitivity of our exposure classification records, VE may be underestimated; truly vaccinated individuals, along with their presumably lower risk of COVID-19, would be included in the unvaccinated pool, and thus decrease estimates of VE. To address this potential misclassification, we performed a QBA to determine how our estimates of VE would change based on hypothesized rates of underreporting of vaccination; for medically diagnosed COVID-19, the observed VE may underestimate the true VE by up to 14%. The extent of vaccination completeness and exposure misclassification was estimated using CDC and state-level estimates from the entire population aged less than 65 years, which may not always perfectly align with the study population. Thus, these bias analyses should be interpreted as suggestive of general estimates of direction and magnitude of bias, and not perfectly corrected estimates. The capture of covariates in health insurance diagnosis data may be affected by pandemic-related reductions in healthcare access and utilization, which also may change over the study period. Follow-up time was generally shorter in the unvaccinated group than the vaccinated group, as unvaccinated individuals could be censored for receiving a first dose of vaccine; additionally, vaccinated individuals could be censored for receiving a booster dose. The selection due to these factors was evaluated with 2 sensitivity analyses addressing informative censoring, and both showed VE results generally comparable to or slightly above those of the primary analyses.

## Conclusions

Receiving a complete primary series of BNT162b2, mRNA-1273, or JNJ-7836735 was associated with reductions in medically diagnosed COVID-19 incidence and hospital/ED-diagnosed COVID-19 in the US adult population, although the observed VE estimates differed across vaccine brands and by variant era. In the rapidly changing dynamics of the COVID-19 pandemic, additional real-world evidence is important for evaluating and understanding changes in VE and impacts of waning immunity on serious COVID-19 outcomes over time.

## Supporting information

S1 Text S1 Supplemental methods **Receiving Only 1 Dose of a 2-Dose Primary Series. S2 Supplemental methods**: **Quantitative Bias Analysis for Exposure Misclassification**. **S1 Table. IIS Jurisdictions and Study Periods Utilized. S2 Table. Details of Follow-up for the Complete Primary Vaccination Series Exposure Patterns. S3 Table. Characteristics of Adults Aged 18-64 Years Vaccinated With BNT162b2 COVID-19 Vaccine and Matched Unvaccinated Comparators. S4 Table. Characteristics of Adults Aged 18-64 Years Vaccinated With an mRNA 1273 COVID-19 Vaccine and Matched Unvaccinated Comparators. S5 Table. Characteristics of Adults Aged 18-64 Years Vaccinated With JNJ-7836735 COVID-19 Vaccine and Matched Unvaccinated Comparators. S6 Table. Estimated Effectiveness of Receiving a Complete Primary Series of COVID-19 Vaccine in Adults Aged 18-64 Years, Compared With Being Unvaccinated, Overall. S7 Table. Estimated Effectiveness of Receiving a Complete Primary Series of COVID-19 Vaccine in Adults Aged 18-64 Years, Compared With Being Unvaccinated, by Previous COVID-19 Diagnosis Status. S8 Table. Estimated Effectiveness of Receiving a Complete Primary Series of COVID-19 Vaccine Compared with Being Unvaccinated, Corrected for Potentially Missing Vaccine Records. S9 Table. Estimated Effectiveness of Receiving a Complete Primary Series of COVID-19 Vaccine Compared With Being Unvaccinated, Over Time. S1 Fig.Propensity Score Distributions of Adults Aged 18-64 Years Receiving a Complete Primary Series of COVID-19 Vaccine and Matched Unvaccinated Comparators. S2 Fig.Estimated Effectiveness of Receiving a Complete Primary Series of COVID-19 Vaccine in Adults Aged 18-64 Years, Compared With Being Unvaccinated, Overall and Within Subgroups. S3 Fig.Estimated Effectiveness of Receiving a Complete Primary Series of COVID-19 Vaccine in Adults Aged 18-64 Years, Compared With Being Unvaccinated, Primary and Sensitivity Analyses. S4 Fig.Weighted Cumulative Incidence of COVID‑19 Outcomes in Adults Aged 18-64 Years Receiving a Complete Primary Series of COVID-19 Vaccines and Matched Unvaccinated Comparators. S5 Fig. Cumulative Incidence of COVID 19 Outcomes in Adults Receiving a Complete Primary Series of COVID-19 Vaccines and Matched Unvaccinated Comparators, Negative Control Outcome in the First 14 Days. S6 Fig.Estimated Effectiveness of Receiving a Complete Primary Series of COVID-19 Vaccine in Adults Aged 18-64 Years, Compared With Being Unvaccinated, by SARS-CoV-2 Variant Era. S7 Fig. Estimated Relative Effectiveness of Receiving a Complete Primary Series of COVID-19 Vaccine in Adults Aged 18-64 Years, Compared With Receiving a Complete Primary Series of Different COVID-19 Vaccines. S8 Fig. Estimated Effectiveness of Receiving a Single Dose of a 2-Dose Primary Series of COVID-19 Vaccine in Adults Aged 18‑64 Years, Compared With Being Unvaccinated. S9 Fig.Weighted Cumulative Incidence of COVID‑19 Outcomes in Adults Aged 18-64 Years Receiving a Single Dose of a 2-Dose Primary Series of COVID-19 Vaccine and Matched Unvaccinated Comparators**.
(DOCX)
