# Supplementary material for: Effectiveness over time of a primary series of the original monovalent COVID-19 vaccines in adults in the United States
Source: PLoS One. 2025 May 6;20(5):e0320434. doi: 10.1371/journal.pone.0320434 (PMC12054878; doi:10.1371/journal.pone.0320434)
Supplement: S1 Text — Receiving Only 1 Dose of a 2-Dose Primary Series. S2 Supplemental methods: Quantitative Bias Analysis for Exposure Misclassification. S1 Table. IIS Jurisdictions and Study Periods Utilized. S2 Table. Details of Follow-up for the Complete Primary Vaccination Series Exposure Patterns. S3 Table. Characteristics of Adults Aged 18-64 Years Vaccinated With BNT162b2 COVID-19 Vaccine and Matched Unvaccinated Comparators. S4 Table. Characteristics of Adults Aged 18-64 Years Vaccinated With an mRNA 1273 COVID-19 Vaccine and Matched Unvaccinated Comparators. S5 Table. Characteristics of Adults Aged 18-64 Years Vaccinated With JNJ-7836735 COVID-19 Vaccine and Matched Unvaccinated Comparators. S6 Table. Estimated Effectiveness of Receiving a Complete Primary Series of COVID-19 Vaccine in Adults Aged 18-64 Years, Compared With Being Unvaccinated, Overall. S7 Table. Estimated Effectiveness of Receiving a Complete Primary Series of COVID-19 Vaccine in Adults Aged 18-64 Years, Compared With Being Unvaccinated, by Previous COVID-19 Diagnosis Status. S8 Table. Estimated Effectiveness of Receiving a Complete Primary Series of COVID-19 Vaccine Compared with Being Unvaccinated, Corrected for Potentially Missing Vaccine Records. S9 Table. Estimated Effectiveness of Receiving a Complete Primary Series of COVID-19 Vaccine Compared With Being Unvaccinated, Over Time. S1 Fig.Propensity Score Distributions of Adults Aged 18-64 Years Receiving a Complete Primary Series of COVID-19 Vaccine and Matched Unvaccinated Comparators. S2 Fig.Estimated Effectiveness of Receiving a Complete Primary Series of COVID-19 Vaccine in Adults Aged 18-64 Years, Compared With Being Unvaccinated, Overall and Within Subgroups. S3 Fig.Estimated Effectiveness of Receiving a Complete Primary Series of COVID-19 Vaccine in Adults Aged 18-64 Years, Compared With Being Unvaccinated, Primary and Sensitivity Analyses. S4 Fig.Weighted Cumulative Incidence of COVID‑19 Outcomes in Adults Aged 18-64 Years Receiving a Complete Pri [file pone.0320434.s001.docx]

Effectiveness over time of a primary series of the original monovalent COVID-19 vaccines in adults in the United States

Supporting Information

J. Bradley Layton^1*^, Patricia C. Lloyd^2^, Lauren S. Peetluk^3^, Yixin Jiao^4^, Djeneba Audrey Djibo^5^, Joann F. Gruber^2^, Jie Deng^3^, Christine Bui^1^, An-Chi Lo^4^, Rachel P. Ogilvie^3^, Ron Parambi^3^, Michael Miller^3^, Jennifer Song^3^, Lisa B. Weatherby^3^, Sylvia Cho^2^, Hui Lee Wong^2#^, Tainya C. Clarke^2^, Jessica Rose Hervol^4^, Dóra Illei^6^, Elizabeth J. Bell^3#^, Grace Wenya Yang^7^, John D. Seeger^3#^, Michael Wernecke^4^, Morgan M. Richey^1#^, Richard A. Forshee^2^, Steven A. Anderson^2^,

Yoganand Chillarige^4^, Cheryl N. McMahill-Walraven^5^, Kandace L. Amend^3^, Mary S. Anthony^1^, Azadeh Shoaibi^2#^

^1^ RTI Health Solutions, Research Triangle Park, North Carolina, United States

^2^ US Food and Drug Administration, Center for Biologics Evaluation and Research, Silver Spring, Maryland, United States

^3^ Optum Epidemiology, Boston, Massachusetts, United States

^4^ Acumen LLC, Burlingame, California, United States

^5^ CVS Health, Safety Surveillance & Collaboration, Blue Bell, Pennsylvania, United States

^6^ RTI International, Washington, DC, United States

^7^ OptumServe, Falls Church, Virginia, United States

^#^ Affiliation at the time of study

*Corresponding author

Email: [jblayton@rti.org](mailto:jblayton@rti.org) (JBL)

## S1 Supplemental methods: receiving only 1 dose of a 2-dose primary series

This secondary analysis evaluated the effectiveness of receiving a single dose of a 2-dose primary series (BNT-162b2 or mRNA-1273), by vaccine brand, compared with being unvaccinated.

The individuals included in this analysis were drawn from the same brand-specific matched analytic cohorts used for the primary analysis (complete vaccine series versus being unvaccinated). Time 0 for both the vaccinated (receipt of Dose 1) and unvaccinated comparator (matched unvaccinated date) groups were the same, and the same 1:1 matching of vaccinated (with an eligible Dose 1 of BNT162b2 or mRNA-1273) and unvaccinated individuals was maintained. However, as this analysis evaluated a different vaccine exposure pattern than the primary analysis (i.e., receiving a single dose of a 2-dose primary series rather than receiving a complete primary series), the censoring criteria for deviation from the vaccine exposure pattern differed for the vaccinated group; vaccinated individuals were censored at the receipt of an additional COVID‑19 vaccine dose after Dose 1, as follows:

| Vaccine exposure pattern | Included individuals | Time 0 (beginning of follow-up) | Deviation from vaccine exposure pattern after Time 0 resulting in censoring |
| --- | --- | --- | --- |
| Single dose of a BNT162b2 primary series | All eligible individuals receiving Dose 1 of BNT162b2 | Date of Dose 1 of BNT162b2 | Receipt of any other COVID-19 vaccine dose |
| Single dose of an mRNA-1273 primary series | All eligible individuals receiving Dose 1 of mRNA-1273 | Date of Dose 1 of mRNA-1273 | Receipt of any other COVID-19 vaccine dose |
| Unvaccinated | Matched eligible unvaccinated comparator individuals | Matched calendar date | Receipt of any COVID-19 vaccine |

COVID-19 = coronavirus disease 2019.

The same propensity scores and IPT weights used in the primary analysis were used in this analysis. The IPT-weighted cumulative incidence curves of COVID-19 outcomes were plotted by exposure group, and HRs and 95% CIs with the corresponding VE were estimated using IPT-weighted Cox proportional hazards models.

## S1 Supplemental methods: quantitative bias analysis for exposure misclassification

Although vaccinations were identified in both health insurance claims data and IIS COVID-19 vaccine records [1], the possibility for missing vaccine information remains. Quantitative bias analyses [2,3] were performed to estimate the impact of truly vaccinated individuals being misclassified as unvaccinated due to missing vaccine records. No gold standard for vaccine status is available, so estimates of statewide receipt of at least 1 COVID-19 vaccine dose among individuals aged younger than 65 years from CDC, state departments of health, and capture-recapture methods [4-6] were obtained and compared with observed state-level estimates in the study data [1] to estimate maximum and minimum potential sensitivities of vaccine exposure.

Using the minimum and maximum sensitivity estimates from CDC, state departments of health, or capture-recapture methods, 2 “corrected” RR estimates (the primary analyses estimated HRs, but for the purposes of the quantitative bias analyses, RRs and 95% CIs were estimated in the weighted cohorts using a fixed 61-day follow-up time for both outcomes)—a minimum and maximum corrected estimate—were generated for each outcome by reassigning exposure status from unvaccinated to vaccinated based on the sensitivity estimate. A correction factor was then estimated as follows:

$$bias correction factor=1-\frac{corrected RR}{uncorrected RR}$$

The bias correction factors were then applied to the observed HR estimates from the primary analyses. Specificity of the study’s vaccine assessment was assumed to be 100% (i.e., all observed claims or IIS records were assumed to be true vaccination events, and no truly unvaccinated individuals were misclassified as being vaccinated).

**References**

1. CBER Surveillance Program, Schneider K, Bell E, Zhou C, et al. *Supplementing administrative claims COVID-19 vaccine data with Immunization Information Systems (IIS) data: A feasibility study report*. U.S. Food & Drug Administration; 2022. <https://bestinitiative.org/wp-content/uploads/2022/08/IIS-Feasibility-Optum-Study-Report-2022.pdf>. Accessed October 2022.
2. Funk MJ, Landi SN. Misclassification in administrative claims data: Quantifying the impact on treatment effect estimates. *Curr Epidemiol Rep*. 2014 Dec;1(4):175-85. doi:<http://dx.doi.org/10.1007/s40471-014-0027-z>.
3. Lash TL, Fox MP, MacLehose RF, et al. Good practices for quantitative bias analysis. *Int J Epidemiol*. 2014 Dec;43(6):1969-85. doi:<http://dx.doi.org/10.1093/ije/dyu149>.
4. International Working Group for Disease Monitoring and Forecasting. Capture-recapture and multiple-record systems estimation I: History and theoretical development. *Am J Epidemiol*. 1995 Nov 15;142(10):1047-58. PMID: 7485050.
5. Seeger JD, Schumock GT, Kong SX. Estimating the rate of adverse drug reactions with capture-recapture analysis. *Am J Health Syst Pharm*. 1996 Jan 15;53(2):178-81. doi:<http://dx.doi.org/10.1093/ajhp/53.2.178>.
6. Hook EB, Regal RR. Capture-recapture methods in epidemiology: Methods and limitations. *Epidemiol Rev*. 1995;17(2):243-64. doi:<http://dx.doi.org/10.1093/oxfordjournals.epirev.a036192>.
7. IIS Jurisdictions and Study Periods Utilized

| IIS Jurisdiction | Optum Study Period | CVS Health Study Period |
| --- | --- | --- |
| 1 | NA | 11 December 2020 – 31 March 2022 |
| 2 | 11 December 2020 – 15 January 2022 | NA |
| 3 | NA | 11 December 2020 – 31 March 2022 |
| 4 | NA | 11 December 2020 – 31 March 2022 |
| 5 | NA | 11 December 2020 – 31 March 2022 |
| 6 | NA | 11 December 2020 – 31 March 2022 |
| 7 | 11 December 2020 – 15 January 2022 | 11 December 2020 – 31 March 2022 |
| 8 | 11 December 2020 – 15 January 2022 | 11 December 2020 – 31 March 2022 |
| 9 | 11 December 2020 – 15 January 2022 | 11 December 2020 – 31 March 2022 |
| 10 | 11 December 2020 – 15 January 2022 | 11 December 2020 – 31 March 2022 |
| 11 | 11 December 2020 – 15 January 2022 | NA |
| 12 | 11 December 2020 – 15 January 2022 | 11 December 2020 – 31 March 2022 |
| 13 | 11 December 2020 – 15 January 2022 | NA |
| 14 | 11 December 2020 – 15 January 2022 | NA |
| 15 | 11 December 2020 – 15 January 2022 | NA |
| 16 | NA | 11 December 2020 – 31 March 2022 |

IIS = Immunization Information System; NA = not applicable.

Note: Optum used data from 10 IIS jurisdictions from 10 US states; CVS Health used data from 11 IIS jurisdictions from 9 unique US states. Sixteen unique IIS jurisdictions were used in the study.

1. Details of Follow-up for the Complete Primary Vaccination Series Exposure Patterns

| Vaccine exposure pattern | Included individuals | Time 0 (beginning of follow-up) | Deviation from vaccine exposure pattern after Time 0 resulting in censoring |
| --- | --- | --- | --- |
| BNT162b2 complete primary series | All eligible individuals receiving Dose 1 of BNT162b2 | Date of Dose 1 of BNT162b2 | - Receipt of Dose 2 of BNT162b2 < 17 days after Dose 1 - Failure to receive Dose 2 of BNT162b2 by day 42 ^a^ - Receipt of any other COVID-19 vaccine brand or unspecified brand - Receipt of a third dose |
| mRNA-1273 complete primary series | All eligible individuals receiving Dose 1 of mRNA-1273 | Date of Dose 1 of mRNA-1273 | - Receipt of Dose 2 of mRNA-1273 < 24 days after Dose 1 - Failure to receive Dose 2 of mRNA-1273 by day 42 ^a^ - Receipt of any other COVID-19 vaccine brand or unspecified brand - Receipt of a third dose |
| JNJ-7836735 complete primary series | All eligible individuals receiving Dose 1 of JNJ-7836735 | Date of Dose 1 of JNJ-7836735 | - Receipt of any other COVID-19 vaccine dose |
| Unvaccinated | Matched eligible unvaccinated comparator individuals | Matched calendar date | - Receipt of any COVID-19 vaccine dose |

COVID-19 = coronavirus disease 2019.

^a^ A 42-day maximum time period was considered for receiving Dose 2 of a 2-dose series.

1. Characteristics of Adults Aged 18-64 Years Vaccinated With BNT162b2 COVID-19 Vaccine and Matched Unvaccinated Comparators

A. Optum

| Characteristic | Individuals vaccinated with BNT162b2  N = 341,097 | Matched unvaccinated individuals  N = 341,097 | ASD |
| --- | --- | --- | --- |
| Characteristics assessed at Time 0 | | | |
| *Age, years* |  |  |  |
| Median (Q1, Q3) | 42 (32, 53) | 42 (32, 53) |  |
| Mean (SD) | 42.03 (12.95) | 42.02 (12.95) | 0.00 |
| *Sex, N (%)* |  |  |  |
| Male | 166,068 (48.69%) | 166,068 (48.69%) | 0.00 |
| Female | 175,029 (51.31%) | 175,029 (51.31%) | 0.00 |
| *Region, N (%)* |  |  |  |
| Northeast | 40,009 (11.73%) | 40,009 (11.73%) | 0.00 |
| South | 86,610 (25.39%) | 86,610 (25.39%) | 0.00 |
| Midwest | 144,657 (42.41%) | 144,657 (42.41%) | 0.00 |
| West | 69,821 (20.47%) | 69,821 (20.47%) | 0.00 |
| Pregnant at Time 0, N (%) | 1,837 (0.54%) | 1,837 (0.54%) | 0.00 |
| Characteristics assessed in the 365 days before Time 0, N (%) | | | |
| *Hospitalizations* |  |  |  |
| 0 | 231,487 (67.87%) | 231,943 (68.00%) | 0.00 |
| 1 | 60,877 (17.85%) | 60,921 (17.86%) | 0.00 |
| 2+ | 48,733 (14.29%) | 48,233 (14.14%) | 0.00 |
| *Emergency department visits* |  |  |  |
| 0 | 307,322 (90.10%) | 303,855 (89.08%) | 0.03 |
| 1 | 27,521 (8.07%) | 29,610 (8.68%) | 0.02 |
| 2+ | 6,254 (1.83%) | 7,632 (2.24%) | 0.03 |
| Skilled nursing facility stay | 536 (0.16%) | 254 (0.07%) | 0.02 |
| Influenza vaccination | 132,439 (38.83%) | 132,439 (38.83%) | 0.00 |
| Pneumococcal vaccination | 4,732 (1.39%) | 4,316 (1.27%) | 0.01 |
| Encounter for cancer screening | 96,432 (28.27%) | 91,430 (26.80%) | 0.03 |
| Eye examination | 21,650 (6.35%) | 20,043 (5.88%) | 0.02 |
| Colonoscopy | 14,213 (4.17%) | 13,179 (3.86%) | 0.02 |
| Bone mineral density test | 4,011 (1.18%) | 3,684 (1.08%) | 0.01 |
| Well-check/well-child preventive healthcare visit | 146,908 (43.07%) | 139,588 (40.92%) | 0.04 |
| Arthritis | 44,721 (13.11%) | 45,255 (13.27%) | 0.00 |
| Lipid abnormality | 60,872 (17.85%) | 58,489 (17.15%) | 0.02 |
| Ambulance use or life support services | 5,239 (1.54%) | 6,070 (1.78%) | 0.02 |
| Weakness | 6,580 (1.93%) | 6,492 (1.90%) | 0.00 |
| Pregnancy completion before Time 0 | 7,183 (4.10%) | 7,389 (4.22%) | 0.01 |
| Characteristics assessed using all available data before Time 0, N (%) | | | |
| Autoimmune disorders | 15,710 (4.61%) | 15,224 (4.46%) | 0.01 |
| Cancer | 20,072 (5.88%) | 19,337 (5.67%) | 0.01 |
| Chronic kidney disease or renal disease | 6,231 (1.83%) | 6,313 (1.85%) | 0.00 |
| Chronic liver disease | 14,482 (4.25%) | 14,576 (4.27%) | 0.00 |
| Chronic lung diseases (e.g., asthma, COPD, cystic fibrosis, pulmonary embolism) | 36,710 (10.76%) | 36,427 (10.68%) | 0.00 |
| Dementia or other neurological conditions | 23,297 (6.83%) | 23,987 (7.03%) | 0.01 |
| Diabetes mellitus, type 1 or 2 | 25,169 (7.38%) | 24,030 (7.04%) | 0.01 |
| Down syndrome | 83 (0.02%) | 61 (0.02%) | 0.00 |
| Heart conditions (e.g., heart failure, coronary artery disease, arrhythmias) | 45,648 (13.38%) | 46,051 (13.50%) | 0.00 |
| Hypertension | 72,219 (21.17%) | 71,842 (21.06%) | 0.00 |
| Immunocompromised state | 15,135 (4.44%) | 15,135 (4.44%) | 0.00 |
| Mental health conditions | 96,696 (28.35%) | 95,899 (28.11%) | 0.01 |
| Obese or severely obese | 71,294 (20.90%) | 70,829 (20.77%) | 0.00 |
| Sickle cell disease or thalassemia | 816 (0.24%) | 904 (0.27%) | 0.01 |
| Stroke or cerebrovascular disease | 5,025 (1.47%) | 5,159 (1.51%) | 0.00 |
| Tuberculosis | 210 (0.06%) | 223 (0.07%) | 0.00 |
| At least 1 COVID-19 laboratory test performed | 127,334 (37.33%) | 115,959 (34.00%) | 0.07 |
| COVID-19 diagnoses occurring outside a hospital or emergency department setting | 18,045 (5.29%) | 17,947 (5.26%) | 0.00 |
| Hospital/ED-diagnosed COVID-19 | 2,810 (0.82%) | 2,777 (0.81%) | 0.00 |

ASD = absolute standardized difference; COVID-19 = coronavirus disease 2019; Q1, Q3 = first and third quartiles; SD = standard deviation.

B. CVS Health

| Characteristic | Individuals vaccinated with BNT162b2  N = 1,151,775 | Matched unvaccinated individuals  N = 1,151,775 | ASD |
| --- | --- | --- | --- |
| Characteristics assessed at Time 0 | | | |
| *Age, years* |  |  |  |
| Median (Q1, Q3) | 42 (31, 53) | 42 (31, 53) | 0.00 |
| Mean (SD) | 41.92 (13.22) | 41.91 (13.23) | 0.00 |
| *Sex, N (%)* |  |  |  |
| Male | 536,224 (46.56%) | 536,224 (46.56%) | 0.00 |
| Female | 615,551 (53.44%) | 615,551 (53.44%) | 0.00 |
| *Region, N (%)* |  |  |  |
| Northeast | 204,777 (17.78%) | 204,777 (17.78%) | 0.00 |
| South | 251,233 (21.81%) | 251,233 (21.81%) | 0.00 |
| Midwest | 206,280 (17.91%) | 206,280 (17.91%) | 0.00 |
| West | 489,485 (42.50%) | 489,485 (42.50%) | 0.00 |
| Pregnant at Time 0, N (%) | 6,611 (0.57%) | 6,611 (0.57%) | 0.00 |
| Characteristics assessed in the 365 days before Time 0, N (%) | | | |
| *Hospitalizations* |  |  |  |
| 0 | 812,173 (70.51%) | 814,894 (70.75%) | 0.01 |
| 1 | 180,041 (15.63%) | 179,387 (15.57%) | 0.00 |
| 2+ | 159,561 (13.85%) | 157,494 (13.67%) | 0.01 |
| *Emergency department visits* |  |  |  |
| 0 | 1,041,322 (90.41%) | 1,027,640 (89.22%) | 0.04 |
| 1 | 88,687 (7.70%) | 96,968 (8.42%) | 0.03 |
| 2+ | 21,766 (1.89%) | 27,167 (2.36%) | 0.03 |
| Skilled nursing facility stay | 837 (0.07%) | 870 (0.08%) | 0.00 |
| Influenza vaccination | 388,801 (33.76%) | 388,801 (33.76%) | 0.00 |
| Pneumococcal vaccination | 15,854 (1.38%) | 14,639 (1.27%) | 0.01 |
| Encounter for cancer screening | 309,502 (26.87%) | 290,259 (25.20%) | 0.04 |
| Eye examination | 89,086 (7.73%) | 80,665 (7.00%) | 0.03 |
| Colonoscopy | 46,035 (4.00%) | 42,535 (3.69%) | 0.02 |
| Bone mineral density test | 16,067 (1.39%) | 14,520 (1.26%) | 0.01 |
| Well-check/well-child preventive healthcare visit | 455,406 (39.54%) | 428,537 (37.21%) | 0.05 |
| Arthritis | 156,828 (13.62%) | 159,792 (13.87%) | 0.01 |
| Lipid abnormality | 215,402 (18.70%) | 207,851 (18.05%) | 0.02 |
| Ambulance use or life support services | 17,857 (1.55%) | 21,695 (1.88%) | 0.03 |
| Weakness | 21,292 (1.85%) | 21,612 (1.88%) | 0.00 |
| Pregnancy completion before Time 0 | 25,139 (2.18%) | 25,011 (2.17%) | 0.00 |
| Characteristics Assessed Using all Available Data, N (%) | | | |
| Autoimmune disorders | 56,025 (4.86%) | 54,760 (4.75%) | 0.01 |
| Cancer | 70,717 (6.14%) | 66,968 (5.81%) | 0.01 |
| Chronic kidney disease or renal disease | 25,567 (2.22%) | 26,629 (2.31%) | 0.01 |
| Chronic liver disease | 56,281 (4.89%) | 56,607 (4.91%) | 0.00 |
| Chronic lung diseases (e.g., asthma, COPD, cystic fibrosis, pulmonary embolism) | 131,033 (11.38%) | 132,083 (11.47%) | 0.00 |
| Dementia or other neurological conditions | 85,608 (7.43%) | 87,745 (7.62%) | 0.01 |
| Diabetes mellitus, type 1 or 2 | 88,515 (7.69%) | 86,908 (7.55%) | 0.01 |
| Down syndrome | 427 (0.04%) | 364 (0.03%) | 0.00 |
| Heart conditions (e.g., heart failure, coronary artery disease, arrhythmias) | 163,592 (14.20%) | 166,283 (14.44%) | 0.01 |
| Hypertension | 243,221 (21.12%) | 243,503 (21.14%) | 0.00 |
| Immunocompromised state | 49,753 (4.32%) | 49,753 (4.32%) | 0.00 |
| Mental health conditions | 323,819 (28.11%) | 326,266 (28.33%) | 0.00 |
| Obese or severely obese | 241,844 (21.00%) | 244,264 (21.21%) | 0.01 |
| Sickle cell disease or thalassemia | 4,042 (0.35%) | 3,904 (0.34%) | 0.00 |
| Stroke or cerebrovascular disease | 20,848 (1.81%) | 22,258 (1.93%) | 0.01 |
| Tuberculosis | 1,145 (0.10%) | 1,098 (0.10%) | 0.00 |
| At least 1 COVID-19 laboratory performed | 527,313 (45.78%) | 484,001 (42.02%) | 0.08 |
| COVID-19 diagnoses occurring outside of a hospital or emergency department | 52,778 (4.58%) | 52,350 (4.55%) | 0.00 |
| Hospitalization or emergency department-diagnosed COVID-19 | 8,983 (0.78%) | 9,161 (0.80%) | 0.00 |

ASD = absolute standardized difference; COVID-19 = coronavirus disease 2019; Q1, Q3 = first and third quartiles; SD = standard deviation.

1. Characteristics of Adults Aged 18-64 Years Vaccinated With an mRNA‑1273 COVID-19 Vaccine and Matched Unvaccinated Comparators

A. Optum

| Characteristic | Individuals vaccinated with mRNA-1273  N = 201,604 | Matched unvaccinated individuals  N = 201,604 | ASD |
| --- | --- | --- | --- |
| **Characteristics assessed at Time 0** | | | |
| *Age, years* |  |  |  |
| Median (Q1, Q3) | 44 (33, 54) | 44 (33, 54) |  |
| Mean (SD) | 43.23 (12.95) | 43.19 (12.97) | 0.00 |
| *Sex, N (%)* |  |  |  |
| Male | 97,657 (48.44%) | 97,657 (48.44%) | 0.00 |
| Female | 103,947 (51.56%) | 103,947 (51.56%) | 0.00 |
| *Region, N (%)* |  |  |  |
| Northeast | 26,580 (13.18%) | 26,580 (13.18%) | 0.00 |
| South | 47,138 (23.38%) | 47,138 (23.38%) | 0.00 |
| Midwest | 85,564 (42.44%) | 85,564 (42.44%) | 0.00 |
| West | 42,322 (20.99%) | 42,322 (20.99%) | 0.00 |
| Pregnant at Time 0, N (%) | 759 (0.38%) | 759 (0.38%) | 0.00 |
| **Characteristics assessed in the 365 days before Time 0, N (%)** | | | |
| *Hospitalizations* |  |  |  |
| 0 | 134,597 (66.76%) | 134,239 (66.59%) | 0.00 |
| 1 | 36,616 (18.16%) | 36,784 (18.25%) | 0.00 |
| 2+ | 30,391 (15.07%) | 30,581 (15.17%) | 0.00 |
| *Emergency department visits* |  |  |  |
| 0 | 181,625 (90.09%) | 179,425 (89.00%) | 0.04 |
| 1 | 16,280 (8.08%) | 17,696 (8.78%) | 0.03 |
| 2+ | 3,699 (1.83%) | 4,483 (2.22%) | 0.03 |
| Skilled nursing facility stay | 210 (0.10%) | 123 (0.06%) | 0.02 |
| Influenza vaccination | 84,003 (41.67%) | 84,003 (41.67%) | 0.00 |
| Pneumococcal vaccination | 3,293 (1.63%) | 2,916 (1.45%) | 0.02 |
| Encounter for cancer screening | 59,499 (29.51%) | 57,384 (28.46%) | 0.02 |
| Eye examination | 13,344 (6.62%) | 12,829 (6.36%) | 0.01 |
| Colonoscopy | 8,905 (4.42%) | 8,366 (4.15%) | 0.01 |
| Bone mineral density test | 2,526 (1.25%) | 2,522 (1.25%) | 0.00 |
| Well-check/well-child preventive healthcare visit | 85,408 (42.36%) | 83,689 (41.51%) | 0.02 |
| Arthritis | 27,688 (13.73%) | 27,843 (13.81%) | 0.00 |
| Lipid abnormality | 40,255 (19.97%) | 38,246 (18.97%) | 0.03 |
| Ambulance use or life support services | 3,211 (1.59%) | 3,669 (1.82%) | 0.02 |
| Weakness | 3,741 (1.86%) | 3,908 (1.94%) | 0.01 |
| Pregnancy completion before Time 0 | 3,593 (3.46%) | 4,090 (3.93%) | 0.03 |
| Characteristics Assessed Using all Available Data, N (%) | | | |
| Autoimmune disorders | 9,343 (4.63%) | 9,329 (4.63%) | 0.00 |
| Cancer | 12,495 (6.20%) | 12,215 (6.06%) | 0.01 |
| Chronic kidney disease or renal disease | 4,027 (2.00%) | 3,925 (1.95%) | 0.00 |
| Chronic liver disease | 9,056 (4.49%) | 9,026 (4.48%) | 0.00 |
| Chronic lung diseases (e.g., asthma, COPD, cystic fibrosis, pulmonary embolism) | 23,005 (11.41%) | 22,562 (11.19%) | 0.01 |
| Dementia or other neurological conditions | 14,078 (6.98%) | 14,325 (7.11%) | 0.00 |
| Diabetes mellitus, type 1 or 2 | 17,003 (8.43%) | 16,045 (7.96%) | 0.02 |
| Down syndrome | 50 (0.02%) | 41 (0.02%) | 0.00 |
| Heart conditions (e.g., heart failure, coronary artery disease, arrhythmias) | 28,052 (13.91%) | 28,853 (14.31%) | 0.01 |
| Hypertension | 47,534 (23.58%) | 46,870 (23.25%) | 0.01 |
| Immunocompromised state | 9,000 (4.46%) | 9,000 (4.46%) | 0.00 |
| Mental health conditions | 59,501 (29.51%) | 58,206 (28.87%) | 0.01 |
| Obese or severely obese | 44,965 (22.30%) | 44,041 (21.85%) | 0.01 |
| Sickle cell disease or thalassemia | 433 (0.21%) | 450 (0.22%) | 0.00 |
| Stroke or cerebrovascular disease | 3,236 (1.61%) | 3,501 (1.74%) | 0.01 |
| Tuberculosis | 142 (0.07%) | 109 (0.05%) | 0.01 |
| At least 1 COVID-19 laboratory performed | 72,155 (35.79%) | 65,871 (32.67%) | 0.07 |
| COVID-19 diagnoses occurring outside of a hospital or emergency department | 9,428 (4.68%) | 9,380 (4.65%) | 0.00 |
| Hospitalization or emergency department-diagnosed COVID-19 | 1,476 (0.73%) | 1,445 (0.72%) | 0.00 |

ASD = absolute standardized difference; COVID-19 = coronavirus disease 2019; Q1, Q3 = first and third quartiles; SD = standard deviation.

B. CVS Health

| Characteristic | Individuals vaccinated with mRNA-1273  N = 651,545 | Matched unvaccinated individuals  N = 651,545 | ASD |
| --- | --- | --- | --- |
| **Characteristics assessed at Time 0** | | | |
| *Age, years* |  |  |  |
| Median (Q1, Q3) | 44 (33, 55) | 44 (32, 55) | 0.00 |
| Mean (SD) | 43.37 (13.4) | 43.33 (13.42) | 0.00 |
| *Sex, N (%)* |  |  |  |
| Male | 313,461 (48.11%) | 313,461 (48.11%) | 0.00 |
| Female | 338,084 (51.89%) | 338,084 (51.89%) | 0.00 |
| *Region, N (%)* |  |  |  |
| Northeast | 139,585 (21.42%) | 139,585 (21.42%) | 0.00 |
| South | 113,510 (17.42%) | 113,510 (17.42%) | 0.00 |
| Midwest | 114,493 (17.57%) | 114,493 (17.57%) | 0.00 |
| West | 283,957 (43.58%) | 283,957 (43.58%) | 0.00 |
| Pregnant at Time 0, N (%) | 2,557 (0.39%) | 2,557 (0.39%) | 0.00 |
| Characteristics in the 365 Days Before Time 0, N (%) | | | |
| *Hospitalizations* |  |  |  |
| 0 | 456,478 (70.06%) | 453,734 (69.64%) | 0.01 |
| 1 | 103,083 (15.82%) | 103,735 (15.92%) | 0.00 |
| 2+ | 91,984 (14.12%) | 94,076 (14.44%) | 0.01 |
| *Emergency department visits* |  |  |  |
| 0 | 587,420 (90.16%) | 580,072 (89.03%) | 0.04 |
| 1 | 51,337 (7.88%) | 55,763 (8.56%) | 0.02 |
| 2+ | 12,788 (1.96%) | 15,710 (2.41%) | 0.03 |
| Skilled nursing facility stay | 567 (0.09%) | 550 (0.08%) | 0.00 |
| Influenza vaccination | 239,416 (36.75%) | 239,416 (36.75%) | 0.00 |
| Pneumococcal vaccination | 10,819 (1.66%) | 9,675 (1.48%) | 0.01 |
| Encounter for cancer screening | 182,176 (27.96%) | 174,072 (26.72%) | 0.03 |
| Eye examination | 52,718 (8.09%) | 48,741 (7.48%) | 0.02 |
| Colonoscopy | 28,306 (4.34%) | 26,338 (4.04%) | 0.02 |
| Bone mineral density test | 10,114 (1.55%) | 9,424 (1.45%) | 0.01 |
| Well-check/well-child preventive healthcare visit | 251,488 (38.60%) | 241,716 (37.10%) | 0.03 |
| Arthritis | 95,284 (14.62%) | 95,699 (14.69%) | 0.00 |
| Lipid abnormality | 140,791 (21.61%) | 134,374 (20.62%) | 0.02 |
| Ambulance use or life support services | 11,600 (1.78%) | 12,988 (1.99%) | 0.02 |
| Weakness | 12,206 (1.87%) | 12,208 (1.87%) | 0.00 |
| Pregnancy completion before Time 0 | 11,057 (1.70%) | 12,237 (1.88%) | 0.01 |
| Characteristics Assessed Using all Available Data, N (%) | | | |
| Autoimmune disorders | 34,439 (5.29%) | 33,671 (5.17%) | 0.01 |
| Cancer | 43,865 (6.73%) | 42,441 (6.51%) | 0.01 |
| Chronic kidney disease or renal disease | 17,312 (2.66%) | 17,201 (2.64%) | 0.00 |
| Chronic liver disease | 35,180 (5.40%) | 35,288 (5.42%) | 0.00 |
| Chronic lung diseases (e.g., asthma, COPD, cystic fibrosis, pulmonary embolism) | 81,057 (12.44%) | 80,729 (12.39%) | 0.00 |
| Dementia or other neurological conditions | 51,559 (7.91%) | 52,066 (7.99%) | 0.00 |
| Diabetes mellitus, type 1 or 2 | 59,510 (9.13%) | 56,297 (8.64%) | 0.02 |
| Down syndrome | 313 (0.05%) | 212 (0.03%) | 0.01 |
| Heart conditions (e.g., heart failure, coronary artery disease, arrhythmias) | 100,214 (15.38%) | 101,431 (15.57%) | 0.01 |
| Hypertension | 158,070 (24.26%) | 156,296 (23.99%) | 0.01 |
| Immunocompromised state | 31,211 (4.79%) | 31,211 (4.79%) | 0.00 |
| Mental health conditions | 193,580 (29.71%) | 191,353 (29.37%) | 0.01 |
| Obese or severely obese | 151,991 (23.33%) | 149,445 (22.94%) | 0.01 |
| Sickle cell disease or thalassemia | 2,011 (0.31%) | 2,055 (0.32%) | 0.00 |
| Stroke or cerebrovascular disease | 14,143 (2.17%) | 14,716 (2.26%) | 0.01 |
| Tuberculosis | 575 (0.09%) | 614 (0.09%) | 0.00 |
| At least 1 COVID-19 laboratory performed | 290,717 (44.62%) | 266,802 (40.95%) | 0.07 |
| COVID-19 diagnoses occurring outside of a hospital or emergency department | 27,212 (4.18%) | 27,004 (4.14%) | 0.00 |
| Hospitalization or emergency department-diagnosed COVID-19 | 4,739 (0.73%) | 4,899 (0.75%) | 0.00 |

ASD = absolute standardized difference; COVID-19 = coronavirus disease 2019; Q1, Q3 = first and third quartiles; SD = standard deviation.

1. Characteristics of Adults Aged 18-64 Years Vaccinated With JNJ-7836735 COVID-19 Vaccine and Matched Unvaccinated Comparators

A. Optum

| Characteristic | Individuals vaccinated with JNJ-7836735  N = 49,285 | Matched unvaccinated individuals  N = 49,285 | ASD |
| --- | --- | --- | --- |
| **Characteristics assessed at Time 0** | | | |
| *Age, years* |  |  |  |
| Median (Q1, Q3) | 44 (33, 54) | 44 (33, 54) |  |
| Mean (SD) | 42.91 (12.88) | 42.89 (12.87) | 0.00 |
| *Sex, N (%)* |  |  |  |
| Male | 27,842 (56.49%) | 27,842 (56.49%) | 0.00 |
| Female | 21,443 (43.51%) | 21,443 (43.51%) | 0.00 |
| *Region, N (%)* |  |  |  |
| Northeast | 5,838 (11.85%) | 5,838 (11.85%) | 0.00 |
| South | 10,032 (20.36%) | 10,032 (20.36%) | 0.00 |
| Midwest | 22,568 (45.79%) | 22,568 (45.79%) | 0.00 |
| West | 10,847 (22.01%) | 10,847 (22.01%) | 0.00 |
| Pregnant at Time 0, N (%) | 72 (0.15%) | 72 (0.15%) | 0.00 |
| Characteristics in the 365 Days Before Time 0, N (%) | | | |
| *Hospitalizations* |  |  |  |
| 0 | 34,682 (70.37%) | 34,074 (69.14%) | 0.03 |
| 1 | 8,388 (17.02%) | 8,557 (17.36%) | 0.01 |
| 2+ | 6,215 (12.61%) | 6,654 (13.50%) | 0.03 |
| *Emergency department visits* |  |  |  |
| 0 | 44,621 (90.54%) | 43,909 (89.09%) | 0.05 |
| 1 | 3,803 (7.72%) | 4,308 (8.74%) | 0.04 |
| 2+ | 861 (1.75%) | 1,068 (2.17%) | 0.03 |
| Skilled nursing facility stay | 31 (0.06%) | 36 (0.07%) | 0.00 |
| Influenza vaccination | 16,343 (33.16%) | 16,343 (33.16%) | 0.00 |
| Pneumococcal vaccination | 568 (1.15%) | 538 (1.09%) | 0.01 |
| Encounter for cancer screening | 13,424 (27.24%) | 12,875 (26.12%) | 0.03 |
| Eye examination | 2,976 (6.04%) | 2,837 (5.76%) | 0.01 |
| Colonoscopy | 2,006 (4.07%) | 1,981 (4.02%) | 0.00 |
| Bone mineral density test | 529 (1.07%) | 493 (1.00%) | 0.01 |
| Well-check/well-child preventive healthcare visit | 19,704 (39.98%) | 18,851 (38.25%) | 0.04 |
| Arthritis | 6,600 (13.39%) | 6,455 (13.10%) | 0.01 |
| Lipid abnormality | 8,711 (17.67%) | 8,650 (17.55%) | 0.00 |
| Ambulance use or life support services | 791 (1.60%) | 890 (1.81%) | 0.02 |
| Weakness | 941 (1.91%) | 924 (1.87%) | 0.00 |
| Pregnancy completion before Time 0 | 568 (2.65%) | 856 (3.99%) | 0.08 |
| Characteristics Assessed Using all Available Data, N (%) | | | |
| Autoimmune disorders | 2,186 (4.44%) | 1,994 (4.05%) | 0.02 |
| Cancer | 2,822 (5.73%) | 2,622 (5.32%) | 0.02 |
| Chronic kidney disease or renal disease | 861 (1.75%) | 929 (1.88%) | 0.01 |
| Chronic liver disease | 1,943 (3.94%) | 2,068 (4.20%) | 0.01 |
| Chronic lung diseases (e.g., asthma, COPD, cystic fibrosis, pulmonary embolism) | 5,011 (10.17%) | 5,031 (10.21%) | 0.00 |
| Dementia or other neurological conditions | 3,168 (6.43%) | 3,115 (6.32%) | 0.00 |
| Diabetes mellitus, type 1 or 2 | 3,400 (6.90%) | 3,489 (7.08%) | 0.01 |
| Down syndrome | < 11 | < 11 | 0.00 |
| Heart conditions (e.g., heart failure, coronary artery disease, arrhythmias) | 6,147 (12.47%) | 6,519 (13.23%) | 0.02 |
| Hypertension | 10,468 (21.24%) | 10,757 (21.83%) | 0.01 |
| Immunocompromised state | 1,865 (3.78%) | 1,865 (3.78%) | 0.00 |
| Mental health conditions | 13,612 (27.62%) | 13,198 (26.78%) | 0.02 |
| Obese or severely obese | 9,764 (19.81%) | 9,928 (20.14%) | 0.01 |
| Sickle cell disease or thalassemia | 63 (0.13%) | 122 (0.25%) | 0.03 |
| Stroke or cerebrovascular disease | 714 (1.45%) | 822 (1.67%) | 0.02 |
| Tuberculosis | 23 (0.05%) | 24 (0.05%) | 0.00 |
| At least 1 COVID-19 laboratory performed | 17,668 (35.85%) | 16,423 (33.32%) | 0.05 |
| COVID-19 diagnoses occurring outside of a hospital or emergency department | 2,761 (5.60%) | 2,749 (5.58%) | 0.00 |
| Hospitalization or emergency department-diagnosed COVID-19 | 393 (0.80%) | 440 (0.89%) | 0.01 |

ASD = absolute standardized difference; COVID-19 = coronavirus disease 2019; Q1, Q3 = first and third quartiles; SD = standard deviation.

Note: Privacy rules require masking cell sizes of fewer than 11 individuals.

B. CVS Health

| Characteristic | Individuals vaccinated with JNJ-7836735  N = 149,813 | Matched unvaccinated individuals  N = 149,813 | ASD |
| --- | --- | --- | --- |
| Characteristics assessed at Time 0 | | | |
| *Age, years* |  |  |  |
| Median (Q1, Q3) | 44 (32, 54) | 44 (32, 54) | 0.00 |
| Mean (SD) | 42.98 (13.31) | 42.97 (13.32) | 0.00 |
| *Sex, N (%)* |  |  |  |
| Male | 82,697 (55.20%) | 82,697 (55.20%) | 0.00 |
| Female | 67,116 (44.80%) | 67,116 (44.80%) | 0.00 |
| *Region, N (%)* |  |  |  |
| Northeast | 29,728 (19.84%) | 29,728 (19.84%) | 0.00 |
| South | 26,237 (17.51%) | 26,237 (17.51%) | 0.00 |
| Midwest | 28,431 (18.98%) | 28,431 (18.98%) | 0.00 |
| West | 65,417 (43.67%) | 65,417 (43.67%) | 0.00 |
| Pregnant at Time 0, N (%) | 274 (0.18%) | 274 (0.18%) | 0.00 |
| Characteristics in the 365 Days Before Time 0, N (%) | | | |
| *Hospitalizations* |  |  |  |
| 0 | 110,435 (73.72%) | 108,225 (72.24%) | 0.03 |
| 1 | 21,706 (14.49%) | 22,499 (15.02%) | 0.01 |
| 2+ | 17,672 (11.80%) | 19,089 (12.74%) | 0.03 |
| *Emergency department visits* |  |  |  |
| 0 | 136,145 (90.88%) | 134,052 (89.48%) | 0.05 |
| 1 | 11,123 (7.42%) | 12,310 (8.22%) | 0.03 |
| 2+ | 2,545 (1.70%) | 3,451 (2.30%) | 0.04 |
| Skilled nursing facility stay | 110 (0.07%) | 103 (0.07%) | 0.00 |
| Influenza vaccination | 44,501 (29.70%) | 44,501 (29.70%) | 0.00 |
| Pneumococcal vaccination | 1,675 (1.12%) | 1,661 (1.11%) | 0.00 |
| Encounter for cancer screening | 37,861 (25.27%) | 36,403 (24.30%) | 0.02 |
| Eye examination | 10,930 (7.30%) | 10,443 (6.97%) | 0.01 |
| Colonoscopy | 6,043 (4.03%) | 5,801 (3.87%) | 0.01 |
| Bone mineral density test | 1,839 (1.23%) | 1,778 (1.19%) | 0.00 |
| Well-check/well-child preventive healthcare visit | 54,304 (36.25%) | 51,984 (34.70%) | 0.03 |
| Arthritis | 21,142 (14.11%) | 20,700 (13.82%) | 0.01 |
| Lipid abnormality | 28,124 (18.77%) | 28,063 (18.73%) | 0.00 |
| Ambulance use or life support services | 2,439 (1.63%) | 2,882 (1.92%) | 0.02 |
| Weakness | 2,689 (1.79%) | 2,676 (1.79%) | 0.00 |
| Pregnancy completion before Time 0 | 1,703 (1.14%) | 2,198 (1.47%) | 0.03 |
| Characteristics Assessed Using all Available Data, N (%) | | | |
| Autoimmune disorders | 6,805 (4.54%) | 6,812 (4.55%) | 0.00 |
| Cancer | 8,817 (5.89%) | 8,438 (5.63%) | 0.01 |
| Chronic kidney disease or renal disease | 3,300 (2.20%) | 3,559 (2.38%) | 0.01 |
| Chronic liver disease | 6,946 (4.64%) | 7,437 (4.96%) | 0.02 |
| Chronic lung diseases (e.g., asthma, COPD, cystic fibrosis, pulmonary embolism) | 16,433 (10.97%) | 16,778 (11.20%) | 0.01 |
| Dementia or other neurological conditions | 10,781 (7.20%) | 10,868 (7.25%) | 0.00 |
| Diabetes mellitus, type 1 or 2 | 10,737 (7.17%) | 11,694 (7.81%) | 0.02 |
| Down syndrome | 51 (0.03%) | 29 (0.02%) | 0.01 |
| Heart conditions (e.g., heart failure, coronary artery disease, arrhythmias) | 20,073 (13.40%) | 21,716 (14.50%) | 0.03 |
| Hypertension | 31,615 (21.10%) | 33,133 (22.12%) | 0.02 |
| Immunocompromised state | 5,561 (3.71%) | 5,561 (3.71%) | 0.00 |
| Mental health conditions | 42,143 (28.13%) | 41,064 (27.41%) | 0.02 |
| Obese or severely obese | 30,537 (20.38%) | 31,435 (20.98%) | 0.01 |
| Sickle cell disease or thalassemia | 359 (0.24%) | 415 (0.28%) | 0.01 |
| Stroke or cerebrovascular disease | 2,563 (1.71%) | 3,018 (2.01%) | 0.02 |
| Tuberculosis | 98 (0.07%) | 131 (0.09%) | 0.01 |
| At least 1 COVID-19 laboratory performed | 67,266 (44.90%) | 61,505 (41.05%) | 0.08 |
| COVID-19 diagnoses occurring outside of a hospital or emergency department | 7,207 (4.81%) | 7,095 (4.74%) | 0.00 |
| Hospitalization or emergency department-diagnosed COVID-19 | 1,076 (0.72%) | 1,271 (0.85%) | 0.01 |

ASD = absolute standardized difference; COVID-19 = coronavirus disease 2019; Q1, Q3 = first and third quartiles; SD = standard deviation.

1. Estimated Effectiveness of Receiving a Complete Primary Series of COVID-19 Vaccine in Adults Aged 18-64 Years, Compared With Being Unvaccinated, Overall

| COVID-19 outcome | Vaccine exposure group | N | Events | Person-time (days) | HR (95% CI) | VE (95% CI) |
| --- | --- | --- | --- | --- | --- | --- |
| **Optum** | | | | | | |
| Medically diagnosed | BNT162b2 | 341,097 | 11,399 | 68,335,369 | 0.44 (0.42-0.45) | 56% (55%-58%) |
|  | Unvaccinated | 341,097 | 15,007 | 41,954,661 | — | — |
|  | mRNA-1273 | 201,604 | 5,691 | 42,145,941 | 0.34 (0.33-0.36) | 66% (64%-67%) |
|  | Unvaccinated | 201,604 | 9,122 | 25,093,307 | — | — |
|  | JNJ-7836735 | 49,285 | 2,215 | 10,249,729 | 0.56 (0.53-0.60) | 44% (40%-47%) |
|  | Unvaccinated | 49,285 | 2,327 | 6,337,889 | — | — |
| Hospital/ED-diagnosed | BNT162b2 | 341,097 | 1,066 | 69,030,682 | 0.18 (0.17-0.20) | 82% (80%-83%) |
|  | Unvaccinated | 341,097 | 3,470 | 42,860,899 | — | — |
|  | mRNA-1273 | 201,604 | 494 | 42,501,963 | 0.13 (0.12-0.14) | 87% (86%-88%) |
|  | Unvaccinated | 201,604 | 2,131 | 25,680,890 | — | — |
|  | JNJ-7836735 | 49,285 | 274 | 10,387,121 | 0.31 (0.26-0.36) | 69% (64%-74%) |
|  | Unvaccinated | 49,285 | 548 | 6,480,273 | — | — |
| **CVS Health** | | | | | | |
| Medically diagnosed | BNT162b2 | 1,151,775 | 40,116 | 268,538,241 | 0.51 (0.5-0.52) | 49% (48%-50%) |
|  | Unvaccinated | 1,151,775 | 47,292 | 167,925,461 | — | — |
|  | mRNA-1273 | 651,545 | 19,347 | 153,442,531 | 0.43 (0.42-0.44) | 57% (56%-58%) |
|  | Unvaccinated | 651,545 | 27,079 | 95,983,285 | — | — |
|  | JNJ-7836735 | 149,813 | 6,931 | 36,342,423 | 0.64 (0.62-0.66) | 36% (34%-38%) |
|  | Unvaccinated | 149,813 | 6,507 | 22,691,059 | — | — |
| Hospital/ED-diagnosed | BNT162b2 | 1,151,775 | 4,496 | 272,234,682 | 0.24 (0.23-0.25) | 76% (75%-77%) |
|  | Unvaccinated | 1,151,775 | 11,464 | 172,260,575 | — | — |
|  | mRNA-1273 | 651,545 | 2,042 | 155,254,572 | 0.18 (0.17-0.19) | 82% (81%-83%) |
|  | Unvaccinated | 651,545 | 6,884 | 98,442,974 | — | — |
|  | JNJ-7836735 | 149,813 | 957 | 37,004,803 | 0.35 (0.32-0.38) | 65% (62%-68%) |
|  | Unvaccinated | 149,813 | 1,702 | 23,256,138 | — | — |

CI = confidence interval; COVID-19 = coronavirus disease 2019; ED = emergency department; HR = hazard ratio; VE = vaccine effectiveness.

Note: — indicates the reference group.

1. Estimated Effectiveness of Receiving a Complete Primary Series of COVID-19 Vaccine in Adults Aged 18-64 Years, Compared With Being Unvaccinated, by Previous COVID-19 Diagnosis Status

| Subgroup | COVID-19 outcome | Vaccine exposure group | N | Events | Person-time (days) | HR (95% CI) | VE (95% CI) |
| --- | --- | --- | --- | --- | --- | --- | --- |
| Optum | | | | | | | |
| With previous COVID-19 diagnosis^a^ | Medically diagnosed | BNT162b2 | 18,962 | 759 | 3,389,905 | 0.66 (0.59-0.74) | 34% (26%-41%) |
|  |  | Unvaccinated | 18,962 | 779 | 2,334,640 | — | — |
|  |  | mRNA-1273 | 9,914 | 341 | 1,857,921 | 0.55 (0.47-0.64) | 45% (36%-53%) |
|  |  | Unvaccinated | 9,914 | 409 | 1,248,699 | — | — |
|  | Hospital/ED-diagnosed | BNT162b2 | 18,962 | 39 | 3,454,998 | 0.28 (0.18-0.43) | 72% (57%-82%) |
|  |  | Unvaccinated | 18,962 | 92 | 2,381,274 | — | — |
|  |  | mRNA-1273 | 9,914 | 16 | 1,886,452 | 0.25 (0.14-0.45) | 75% (55%-86%) |
|  |  | Unvaccinated | 9,914 | 42 | 1,280,414 | — | — |
| Without a previous COVID-19 diagnosis | Medically diagnosed | BNT162b2 | 322,135 | 10,640 | 64,945,464 | 0.42 (0.41-0.44) | 58% (56%-59%) |
|  |  | Unvaccinated | 322,135 | 14,228 | 39,620,021 | — | — |
|  |  | mRNA-1273 | 191,690 | 5,350 | 40,288,020 | 0.33 (0.32-0.35) | 67% (65%-68%) |
|  |  | Unvaccinated | 191,690 | 8,713 | 23,844,608 | — | — |
|  | Hospital/ED-diagnosed | BNT162b2 | 322,135 | 1,027 | 65,575,684 | 0.18 (0.16-0.19) | 82% (81%-84%) |
|  |  | Unvaccinated | 322,135 | 3,378 | 40,479,625 | — | — |
|  |  | mRNA-1273 | 191,690 | 478 | 40,615,511 | 0.13 (0.12-0.14) | 87% (86%-88%) |
|  |  | Unvaccinated | 191,690 | 2,089 | 24,400,476 | — | — |
| CVS Health | | | | | | | |
| With previous COVID-19 diagnosis ^a^ | Medically diagnosed | BNT162b2 | 56,266 | 2,768 | 12,001,685 | 0.71 (0.67-0.75) | 29% (25%-33%) |
|  |  | Unvaccinated | 56,266 | 2,745 | 8,365,317 | — | — |
|  |  | mRNA-1273 | 29,029 | 1,350 | 6,297,879 | 0.66 (0.61-0.72) | 34% (28%-39%) |
|  |  | Unvaccinated | 29,029 | 1,436 | 4,371,147 | — | — |
|  |  | JNJ-7836735 | 7,660 | 406 | 1,743,282 | 0.74 (0.64-0.85) | 26% (15%-36%) |
|  |  | Unvaccinated | 7,660 | 371 | 1,164,613 | — | — |
|  | Hospital/ED-diagnosed | BNT162b2 | 56,266 | 186 | 12,324,343 | 0.44 (0.35-0.53) | 56% (47%-65%) |
|  |  | Unvaccinated | 56,266 | 306 | 8,644,775 | — | — |
|  |  | mRNA-1273 | 29,029 | 87 | 6,472,352 | 0.34 (0.26-0.45) | 66% (55%-74%) |
|  |  | Unvaccinated | 29,029 | 174 | 4,519,359 | — | — |
|  |  | JNJ-7836735 | 7,660 | 28 | 1,790,309 | 0.59 (0.36-0.99) | 41% (1%-64%) |
|  |  | Unvaccinated | 7,660 | 34 | 1,198,980 | — | — |
| Without a previous COVID-19 diagnosis | Medically diagnosed | BNT162b2 | 1,095,509 | 37,348 | 256,536,556 | 0.50 (0.49-0.50) | 50% (50%-51%) |
|  |  | Unvaccinated | 1,095,509 | 44,547 | 159,560,144 | — | — |
|  |  | mRNA-1273 | 622,516 | 17,997 | 147,144,652 | 0.42 (0.41-0.42) | 58% (58%-59%) |
|  |  | Unvaccinated | 622,516 | 25,643 | 91,612,138 | — | — |
|  |  | JNJ-7836735 | 142,153 | 6,525 | 34,599,141 | 0.63 (0.61-0.65) | 37% (35%-39%) |
|  |  | Unvaccinated | 142,153 | 6,136 | 21,526,446 | — | — |
|  | Hospital/ED-diagnosed | BNT162b2 | 1,095,509 | 4,310 | 259,910,339 | 0.24 (0.23-0.25) | 76% (75%-77%) |
|  |  | Unvaccinated | 1,095,509 | 11,158 | 163,615,800 | — | — |
|  |  | mRNA-1273 | 622,516 | 1,955 | 148,782,220 | 0.17 (0.17-0.18) | 83% (82%-83%) |
|  |  | Unvaccinated | 622,516 | 6,710 | 93,923,615 | — | — |
|  |  | JNJ-7836735 | 142,153 | 929 | 35,214,494 | 0.34 (0.32-0.37) | 66% (63%-68%) |
|  |  | Unvaccinated | 142,153 | 1,668 | 22,057,158 | — | — |

CI = confidence interval; COVID-19 = coronavirus disease 2019; ED = emergency department; HR = hazard ratio; VE = vaccine effectiveness.

Note: — indicates the reference group.

^a^ Having a diagnosis of COVID-19 in any medically attended setting at any point before Time 0.

1. Estimated Effectiveness of Receiving a Complete Primary Series of COVID-19 Vaccine Compared with Being Unvaccinated, Corrected for Potentially Missing Vaccine Records

| COVID-19 outcome | Vaccine | Hypothesized sensitivity of vaccination exposure measurement | Corrected VE (95% CI) |
| --- | --- | --- | --- |
| Optum | | | |
| Medically diagnosed | BNT162b2 | 100% (uncorrected) | 56% (55%-58%) |
|  |  | 83% | 61% (60%-63%) |
|  |  | 71% | 67% (66%-68%) |
|  | mRNA-1273 | 100% (uncorrected) | 66% (64%-67%) |
|  |  | 83% | 70% (68%-71%) |
|  |  | 71% | 75% (74%-76%) |
|  | JNJ-7836735 | 100% (uncorrected) | 44% (40%-47%) |
|  |  | 83% | 49% (45%-52%) |
|  |  | 71% | 56% (53%-58%) |
| Hospital/ED-diagnosed | BNT162b2 | 100% (uncorrected) | 82% (80%-83%) |
|  |  | 83% | 85% (83%-85%) |
|  |  | 71% | 88% (86%-88%) |
|  | mRNA-1273 | 100% (uncorrected) | 87% (86%-88%) |
|  |  | 83% | 89% (88%-90%) |
|  |  | 71% | 91% (91%-92%) |
|  | JNJ-7836735 | 100% (uncorrected) | 69% (64%-74%) |
|  |  | 83% | 73% (69%-77%) |
|  |  | 71% | 78% (74%-81%) |
| CVS Health | | | |
| Medically diagnosed | BNT162b2 | 100% (uncorrected) | 49% (48%-50%) |
|  |  | 89% | 52% (52%-53%) |
|  |  | 69% | 63% (62%-64%) |
|  | mRNA-1273 | 100% (uncorrected) | 57% (56%-58%) |
|  |  | 89% | 60% (59%-61%) |
|  |  | 69% | 69% (69%-70%) |
|  | JNJ-7836735 | 100% (uncorrected) | 36% (34%-38%) |
|  |  | 89% | 38% (36%-40%) |
|  |  | 69% | 46% (44%-48%) |
| Hospital/ED-diagnosed | BNT162b2 | 100% (uncorrected) | 76% (75%-77%) |
|  |  | 89% | 78% (77%-79%) |
|  |  | 69% | 84% (83%-85%) |
|  | mRNA-1273 | 100% (uncorrected) | 82% (81%-83%) |
|  |  | 89% | 84% (83%-85%) |
|  |  | 69% | 89% (88%-89%) |
|  | JNJ-7836735 | 100% (uncorrected) | 65% (62%-68%) |
|  |  | 89% | 67% (64%-70%) |
|  |  | 69% | 74% (72%-76%) |

CI = confidence interval; COVID-19 = coronavirus disease 2019; VE = vaccine effectiveness.

1. Estimated Effectiveness of Receiving a Complete Primary Series of COVID-19 Vaccine Compared With Being Unvaccinated, Over Time

| Vaccine | Time Point | Medically diagnosed COVID-19 | | Hospital/ED-diagnosed COVID-19 | |
| --- | --- | --- | --- | --- | --- |
|  |  | VE (95% CI) | RD (95% CI) | VE (95% CI) | RD (95% CI) |
| **Optum** | | | | | |
| BNT162b2 | Day 14 | 22% (16%-28%) | -0.0008 (-0.0011 to -0.0006) | 32% (17%-45%) | -0.0002 (-0.0003 to -0.0001) |
|  | Day 28 | 30% (25%-34%) | -0.0017 (-0.0020 to -0.0014) | 41% (29%-51%) | -0.0004 (-0.0006 to -0.0003) |
|  | Day 42 | 43% (39%-46%) | -0.0039 (-0.0044 to -0.0035) | 57% (50%-64%) | -0.0010 (-0.0012 to -0.0008) |
|  | Day 60 | 57% (55%-59%) | -0.0084 (-0.0089 to -0.0079) | 73% (68%-77%) | -0.0023 (-0.0025 to -0.0020) |
|  | Day 90 | 64% (63%-66%) | -0.0135 (-0.0142 to -0.0128) | 79% (77%-82%) | -0.0036 (-0.0039 to -0.0033) |
|  | Day 183 | 65% (64%-67%) | -0.0367 (-0.0379 to -0.0353) | 87% (85%-88%) | -0.0116 (-0.0123 to -0.0111) |
|  | Day 270 | 56% (55%-58%) | -0.0564 (-0.0582 to -0.0544) | 83% (82%-85%) | -0.0202 (-0.0212 to -0.0193) |
| mRNA-1273 | Day 14 | 26% (18%-34%) | -0.0011 (-0.0015 to -0.0007) | 46% (29%-59%) | -0.0003 (-0.0005 to -0.0002) |
|  | Day 28 | 46% (41%-50%) | -0.0038 (-0.0043 to -0.0032) | 63% (55%-71%) | -0.0010 (-0.0012 to -0.0008) |
|  | Day 42 | 54% (50%-57%) | -0.0062 (-0.0068 to -0.0056) | 72% (66%-77%) | -0.0017 (-0.0019 to -0.0014) |
|  | Day 60 | 63% (60%-65%) | -0.0097 (-0.0104 to -0.0090) | 79% (74%-82%) | -0.0025 (-0.0028 to -0.0022) |
|  | Day 90 | 69% (67%-71%) | -0.0148 (-0.0157 to -0.0140) | 83% (80%-86%) | -0.0038 (-0.0041 to -0.0034) |
|  | Day 183 | 74% (73%-75%) | -0.0404 (-0.0419 to -0.0389) | 91% (89%-92%) | -0.0119 (-0.0127 to -0.0112) |
|  | Day 270 | 68% (67%-69%) | -0.0680 (-0.0704 to -0.0654) | 88% (87%-90%) | -0.0216 (-0.0229 to -0.0205) |
| JNJ-7836735 | Day 14 | 24% (5%-39%) | -0.0009 (-0.0017 to -0.0002) | 42% (7%-66%) | -0.0004 (-0.0007 to -0.0001) |
|  | Day 60 | 51% (43%-57%) | -0.0075 (-0.0089 to -0.0060) | 64% (51%-74%) | -0.0023 (-0.0030 to -0.0016) |
|  | Day 90 | 55% (49%-60%) | -0.0117 (-0.0136 to -0.0099) | 70% (61%-78%) | -0.0035 (-0.0044 to -0.0027) |
|  | Day 183 | 52% (49%-56%) | -0.0298 (-0.0333 to -0.0266) | 74% (69%-79%) | -0.0106 (-0.0124 to -0.0090) |
|  | Day 270 | 42% (39%-46%) | -0.0441 (-0.0500 to -0.0388) | 69% (64%-75%) | -0.0175 (-0.0202 to -0.0151) |
| **CVS Health** | | | | | |
| BNT162b2 | Day 14 | 25% (21%-28%) | -0.0008 (-0.0009 to -0.0007) | 34% (25%-42%) | -0.0002 (-0.0003 to -0.0001) |
|  | Day 28 | 38% (36%-40%) | -0.0024 (-0.0026 to -0.0022) | 48% (43%-53%) | -0.0006 (-0.0007 to -0.0005) |
|  | Day 42 | 48% (47%-50%) | -0.0043 (-0.0046 to -0.0041) | 62% (58%-65%) | -0.0012 (-0.0013 to -0.0011) |
|  | Day 60 | 56% (55%-58%) | -0.0067 (-0.0070 to -0.0065) | 70% (68%-72%) | -0.0018 (-0.0020 to -0.0017) |
|  | Day 90 | 62% (61%-64%) | -0.0106 (-0.0109 to -0.0103) | 77% (75%-79%) | -0.0029 (-0.0031 to -0.0028) |
|  | Day 183 | 62% (62%-63%) | -0.0272 (-0.0277 to -0.0267) | 83% (82%-84%) | -0.0090 (-0.0092 to -0.0087) |
|  | Day 270 | 54% (53%-54%) | -0.0416 (-0.0425 to -0.0407) | 79% (79%-80%) | -0.0155 (-0.0159 to -0.0151) |
| mRNA-1273 | Day 14 | 16% (10%-21%) | -0.0005 (-0.0007 to -0.0003) | 42% (31%-51%) | -0.0002 (-0.0003 to -0.0002) |
|  | Day 28 | 39% (36%-42%) | -0.0024 (-0.0026 to -0.0022) | 62% (57%-67%) | -0.0008 (-0.0009 to -0.0007) |
|  | Day 42 | 49% (47%-51%) | -0.0044 (-0.0047 to -0.0041) | 72% (69%-75%) | -0.0014 (-0.0015 to -0.0012) |
|  | Day 60 | 58% (56%-60%) | -0.0069 (-0.0073 to -0.0066) | 78% (76%-81%) | -0.0021 (-0.0022 to -0.0019) |
|  | Day 90 | 65% (64%-67%) | -0.0109 (-0.0113 to -0.0105) | 84% (82%-85%) | -0.0032 (-0.0034 to -0.0031) |
|  | Day 183 | 71% (70%-72%) | -0.0295 (-0.0303 to -0.0288) | 89% (88%-90%) | -0.0097 (-0.0101 to -0.0093) |
|  | Day 270 | 65% (64%-66%) | -0.0499 (-0.0510 to -0.0487) | 86% (85%-87%) | -0.0177 (-0.0183 to -0.0170) |
| JNJ-7836735 | Day 14 | 10% (-5% to 24%) | -0.0002 (-0.0006 to 0.0001) | -16% (-68% to 22%) | 0.0001 (-0.0001 to 0.0002) |
|  | Day 28 | 26% (18%-34%) | -0.0013 (-0.0018 to -0.0008) | 31% (11%-47%) | -0.0004 (-0.0006 to -0.0001) |
|  | Day 42 | 33% (27%-40%) | -0.0024 (-0.0031 to -0.0019) | 48% (35%-59%) | -0.0008 (-0.0011 to -0.0006) |
|  | Day 60 | 39% (35%-44%) | -0.0042 (-0.0048 to -0.0035) | 55% (46%-64%) | -0.0014 (-0.0017 to -0.0010) |
|  | Day 90 | 43% (39%-47%) | -0.0069 (-0.0077 to -0.0060) | 61% (55%-68%) | -0.0025 (-0.0029 to -0.0020) |
|  | Day 183 | 46% (43%-48%) | -0.0204 (-0.0220 to -0.0188) | 69% (66%-73%) | -0.0083 (-0.0091 to -0.0075) |
|  | Day 270 | 39% (37%-41%) | -0.0326 (-0.0350 to -0.0300) | 68% (65%-71%) | -0.0150 (-0.0162 to -0.0138) |

CI = confidence interval; COVID-19 = coronavirus disease 2019; ED = emergency department; RD = risk difference; VE = vaccine effectiveness.

1. Propensity Score Distributions of Adults Aged 18-64 Years Receiving a Complete Primary Series of COVID-19 Vaccine and Matched Unvaccinated Comparators

A. BNT162b2 vs. Unvaccinated Comparators, Optum


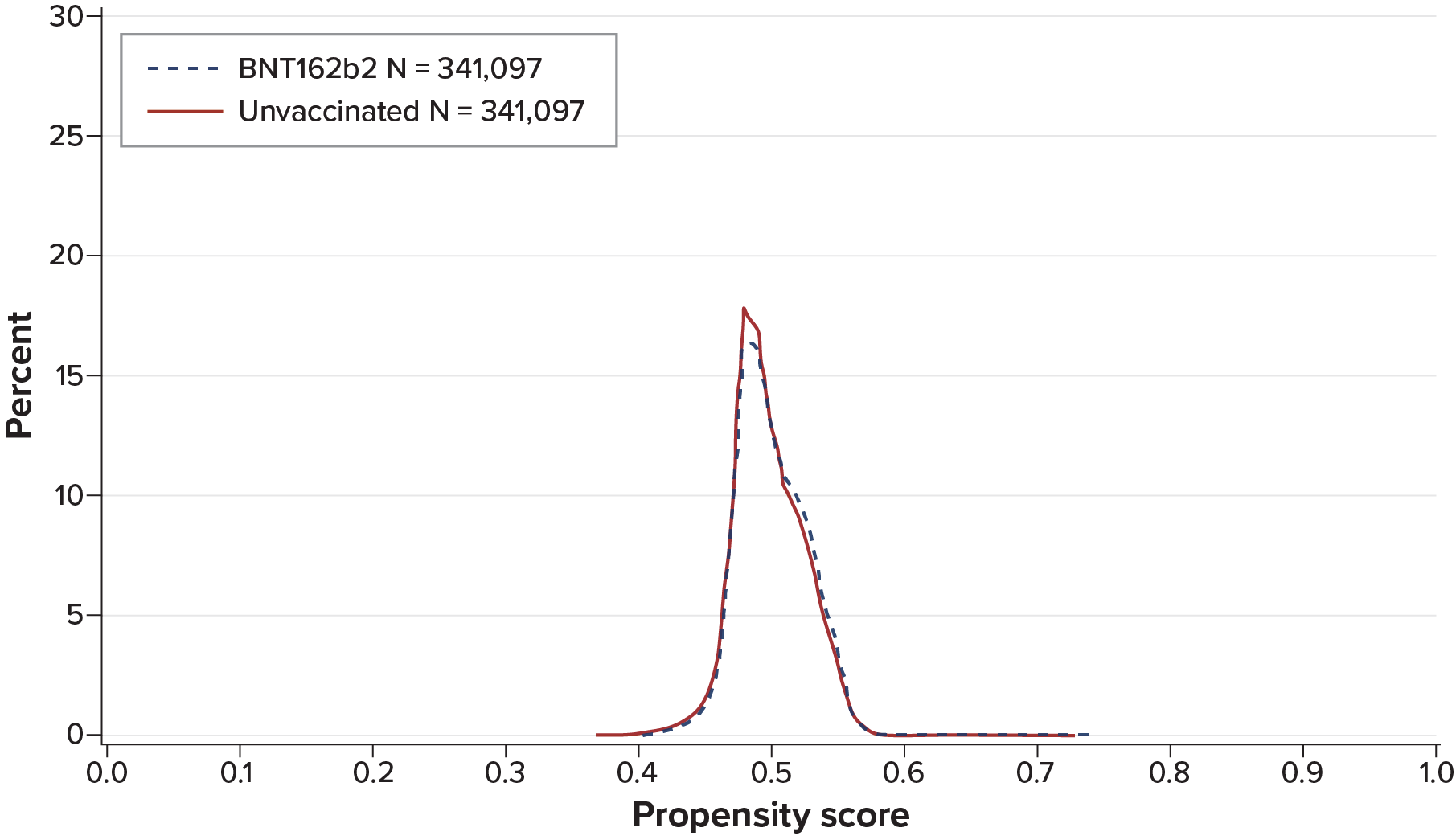


Note: Propensity score variables included: age at index date (linear); female sex indicator; state indicator (categorical with indicator variables); pregnant at time 0; hospitalizations (categorical with indicator variables: 2+, 1, reference = 0); ED visits (categorical with indicator variables: 2+, 1, reference = 0); skilled nursing facility stay indicator; influenza vaccination indicator; pneumococcal vaccination indicator; encounter for cancer screening indicator; eye examination indicator; colonoscopy indicator; bone mineral density test indicator; well-check/well-child preventive healthcare visit indicator; arthritis indicator; lipid abnormality indicator; ambulance use or life support services indicator; weakness indicator; autoimmune disorders indicator; cancer indicator; chronic kidney disease or renal disease indicator; chronic liver disease indicator; chronic lung diseases (e.g., asthma, COPD, cystic fibrosis, pulmonary embolism) indicator; dementia or other neurological conditions indicator; diabetes mellitus, type 1 or 2 indicator; down syndrome indicator; heart conditions (e.g., heart failure, coronary artery disease, arrhythmias) indicator; hypertension indicator; immunocompromised state (identified through diagnoses of immunocompromising conditions and use of immunosuppressive therapies) indicator; mental health conditions indicator; obese or severely obese indicator; sickle cell disease or thalassemia indicator; stroke or cerebrovascular disease indicator; tuberculosis indicator; at least 1 COVID-19 laboratory performed indicator; COVID-19 diagnosis in any setting indicator; delta or omicron variant era indicator; increased risk of COVID-19 indicator; Delta/Omicron era indicator * COVID-19 laboratory test indicator (interaction term).

B. BNT162b2 vs. Unvaccinated Comparators, CVS Health


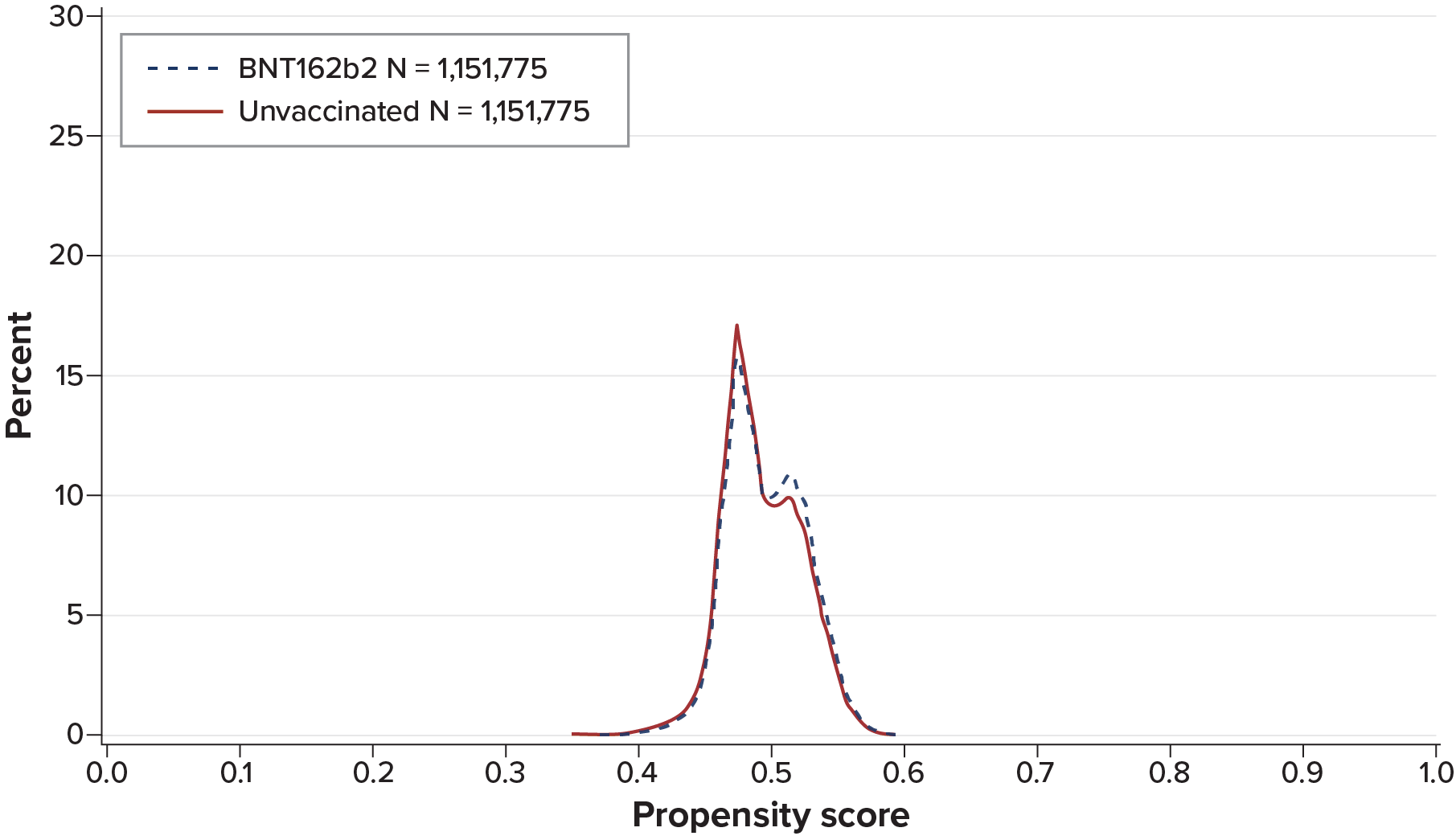


Note: Propensity score variables included: age (categorical with indicator variables:18-24, 25-29, 30-34, 35-39, 40-44, 45-49, 50-54, 55-59, 60-64 years); ambulance use of life support services indicator; arthritis indicator; autoimmune disorders indicator; bone mineral density test indicator; cancer indicator; encounter for cancer screening indicator; chronic kidney disease or renal disease indicator; chronic liver disease indicator; colonoscopy indicator; chronic lung diseases (e.g., asthma, COPD, cystic fibrosis, pulmonary embolism) indicator; county Federal Information Processing System (FIPS) codes (categorical with indicator variables); pregnant at time 0 indicator; COVID-19 diagnoses in any setting indicator; hospitalization or emergency department-diagnosed COVID-19 indicator; at least 1 COVID-19 laboratory performed indicator, COVID-19 diagnoses occurring outside of hospital or ED indicator; COVID-19 vaccination after 31 May 2021 * 1 or more COVID-19 laboratory tests performed (interaction term); COVID-19 vaccination index date in the Delta/Omicron era indicator; diabetes mellitus, type 1 or 2 indicator; Down syndrome indicator; ED visits (categorical with indicator variables: 2+, 1, reference = 0); eye examination indicator; influenza vaccination indicator; heart conditions (e.g., heart failure, coronary artery disease, arrhythmias) indicator; overall binary indicator of the presence of any of these conditions will be defined to identify individuals who may qualify for priority groups for vaccination eligibility; hypertension indicator; hospitalizations (categorical with indicator variables: 2+, 1, reference = 0); lipid abnormality indicator; mental health conditions indicator; dementia or other neurological conditions indicator; obese or severely obese indicator; pneumococcal vaccination indicator; female indicator; male indicator; sickle cell disease or thalassemia indicator; skilled nursing facility stay indicator; IIS jurisdiction (categorical with indicator variables); stroke or cerebrovascular disease indicator; tuberculosis indicator; immunocompromised state indicator; pregnancy completion before time 0 indicator; weakness indicator; well-check/well-child preventive healthcare visit indicator.

C. mRNA-1273 vs. Unvaccinated Comparators, Optum


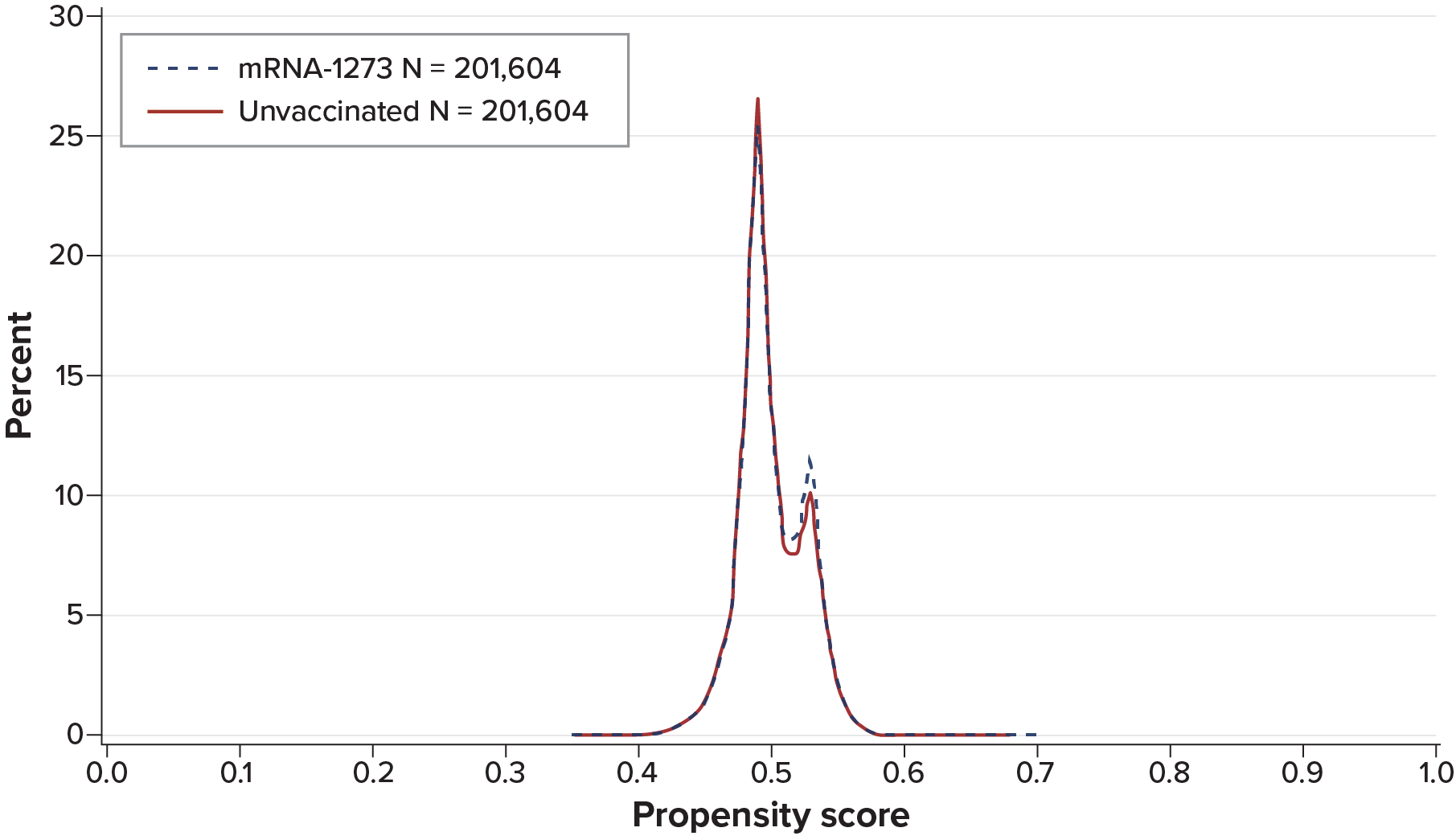


Note: Propensity score variables included: age at index date (linear); female sex indicator; state indicator (categorical with indicator variables); pregnant at time 0; hospitalizations (categorical with indicator variables: 2+, 1, reference = 0); ED visits (categorical with indicator variables: 2+, 1, reference = 0); skilled nursing facility stay indicator; influenza vaccination indicator; pneumococcal vaccination indicator; encounter for cancer screening indicator; eye examination indicator; colonoscopy indicator; bone mineral density test indicator; well-check/well-child preventive healthcare visit indicator; arthritis indicator; lipid abnormality indicator; ambulance use or life support services indicator; weakness indicator; autoimmune disorders indicator; cancer indicator; chronic kidney disease or renal disease indicator; chronic liver disease indicator; chronic lung diseases (e.g., asthma, COPD, cystic fibrosis, pulmonary embolism) indicator; dementia or other neurological conditions indicator; diabetes mellitus, type 1 or 2 indicator; down syndrome indicator; heart conditions (e.g., heart failure, coronary artery disease, arrhythmias) indicator; hypertension indicator; immunocompromised state (identified through diagnoses of immunocompromising conditions and use of immunosuppressive therapies) indicator; mental health conditions indicator; obese or severely obese indicator; sickle cell disease or thalassemia indicator; stroke or cerebrovascular disease indicator; tuberculosis indicator; at least 1 COVID-19 laboratory performed indicator; COVID-19 diagnosis in any setting indicator; delta or omicron variant era indicator; increased risk of COVID-19 indicator; Delta/Omicron era indicator * COVID-19 laboratory test indicator (interaction term).

D. mRNA-1273 vs. Unvaccinated Comparators, CVS Health


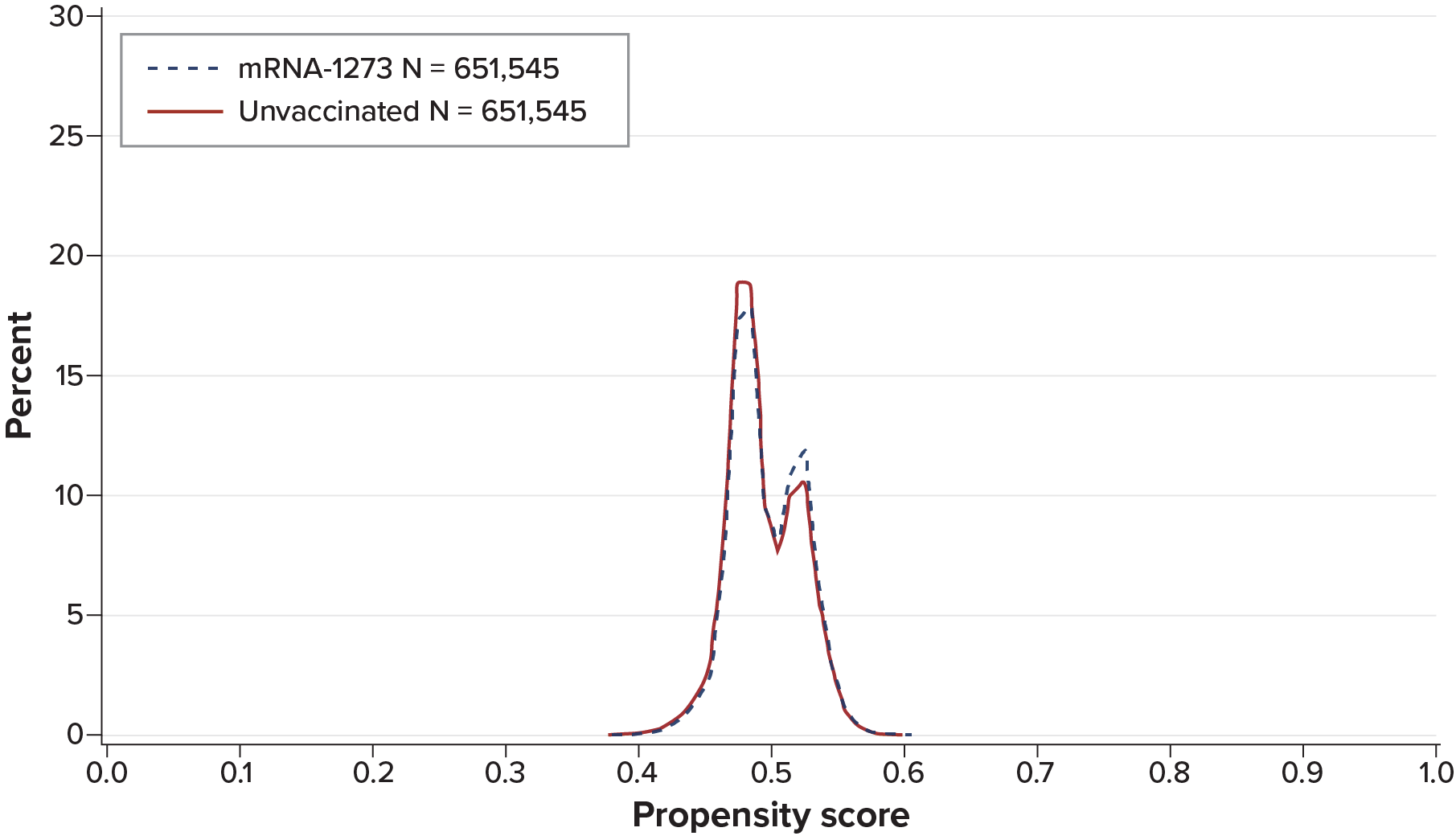


Note: Propensity score variables included: age (categorical with indicator variables:18-24, 25-29, 30-34, 35-39, 40-44, 45-49, 50-54, 55-59, 60-64 years); ambulance use of life support services indicator; arthritis indicator; autoimmune disorders indicator; bone mineral density test indicator; cancer indicator; encounter for cancer screening indicator; chronic kidney disease or renal disease indicator; chronic liver disease indicator; colonoscopy indicator; chronic lung diseases (e.g., asthma, COPD, cystic fibrosis, pulmonary embolism) indicator; county Federal Information Processing System (FIPS) codes (categorical with indicator variables); pregnant at time 0 indicator; COVID-19 diagnoses in any setting indicator; hospitalization or emergency department-diagnosed COVID-19 indicator; at least 1 COVID-19 laboratory performed indicator, COVID-19 diagnoses occurring outside of hospital or ED indicator; COVID-19 vaccination after 31 May 2021 * 1 or more COVID-19 laboratory tests performed (interaction term); COVID-19 vaccination index date in the Delta/Omicron era indicator; diabetes mellitus, type 1 or 2 indicator; Down syndrome indicator; ED visits (categorical with indicator variables: 2+, 1, reference = 0); eye examination indicator; influenza vaccination indicator; heart conditions (e.g., heart failure, coronary artery disease, arrhythmias) indicator; overall binary indicator of the presence of any of these conditions will be defined to identify individuals who may qualify for priority groups for vaccination eligibility; hypertension indicator; hospitalizations (categorical with indicator variables: 2+, 1, reference = 0); lipid abnormality indicator; mental health conditions indicator; dementia or other neurological conditions indicator; obese or severely obese indicator; pneumococcal vaccination indicator; female indicator; male indicator; sickle cell disease or thalassemia indicator; skilled nursing facility stay indicator; IIS jurisdiction (categorical with indicator variables); stroke or cerebrovascular disease indicator; tuberculosis indicator; immunocompromised state indicator; pregnancy completion before time 0 indicator; weakness indicator; well-check/well-child preventive healthcare visit indicator.

E. JNJ-7836735 vs. Unvaccinated Comparators, Optum


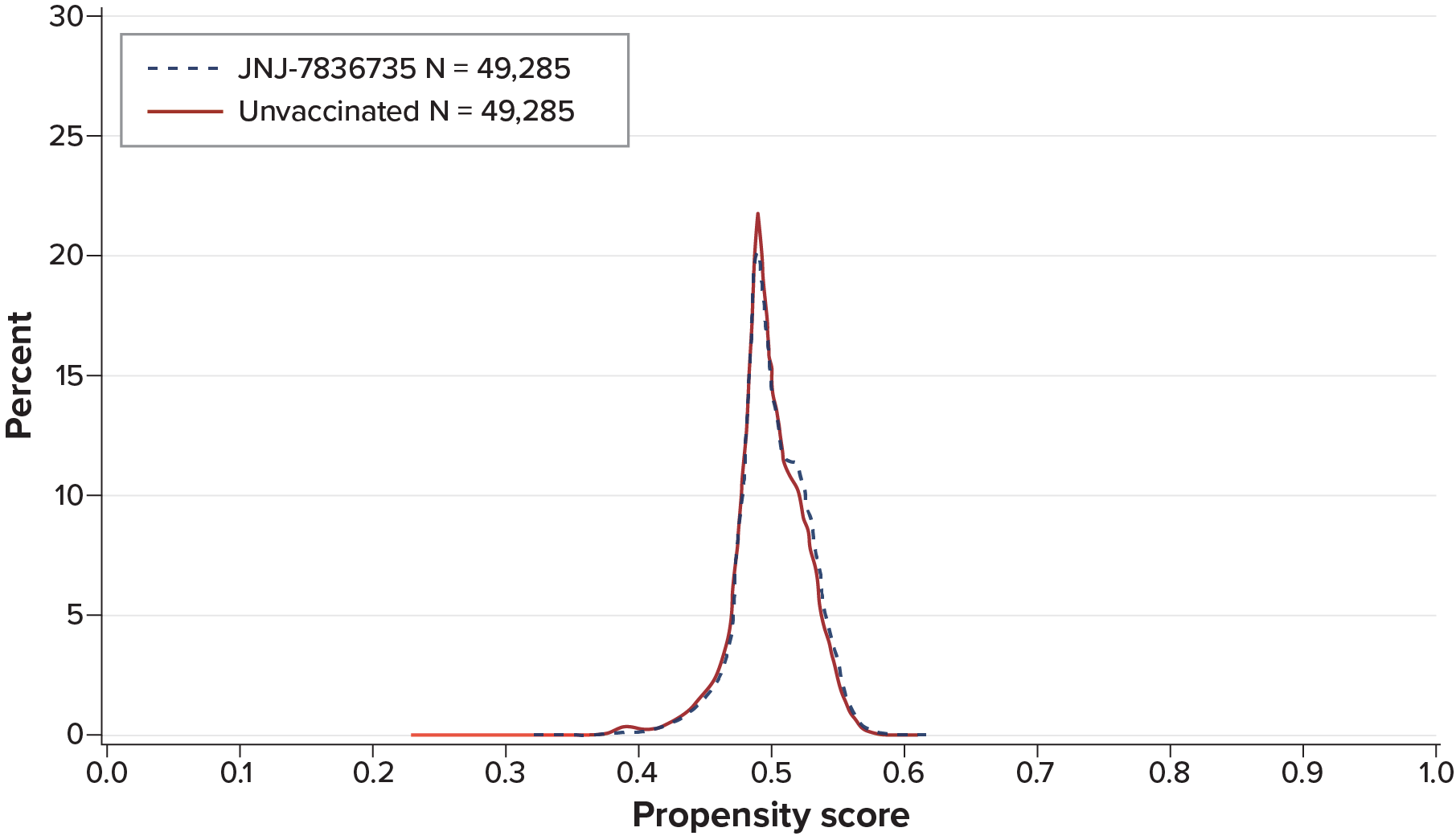


Note: Propensity score variables included: age at index date (linear); female sex indicator; state indicator (categorical with indicator variables); pregnant at time 0; hospitalizations (categorical with indicator variables: 2+, 1, reference = 0); ED visits (categorical with indicator variables: 2+, 1, reference = 0); skilled nursing facility stay indicator; influenza vaccination indicator; pneumococcal vaccination indicator; encounter for cancer screening indicator; eye examination indicator; colonoscopy indicator; bone mineral density test indicator; well-check/well-child preventive healthcare visit indicator; arthritis indicator; lipid abnormality indicator; ambulance use or life support services indicator; weakness indicator; autoimmune disorders indicator; cancer indicator; chronic kidney disease or renal disease indicator; chronic liver disease indicator; chronic lung diseases (e.g., asthma, COPD, cystic fibrosis, pulmonary embolism) indicator; dementia or other neurological conditions indicator; diabetes mellitus, type 1 or 2 indicator; down syndrome indicator; heart conditions (e.g., heart failure, coronary artery disease, arrhythmias) indicator; hypertension indicator; immunocompromised state (identified through diagnoses of immunocompromising conditions and use of immunosuppressive therapies) indicator; mental health conditions indicator; obese or severely obese indicator; sickle cell disease or thalassemia indicator; stroke or cerebrovascular disease indicator; tuberculosis indicator; at least 1 COVID-19 laboratory performed indicator; COVID-19 diagnosis in any setting indicator; Delta or Omicron variant era indicator; increased risk of COVID-19 indicator; Delta/Omicron era indicator * COVID-19 laboratory test indicator (interaction term).

F. JNJ-7836735 vs. Unvaccinated Comparators, CVS Health


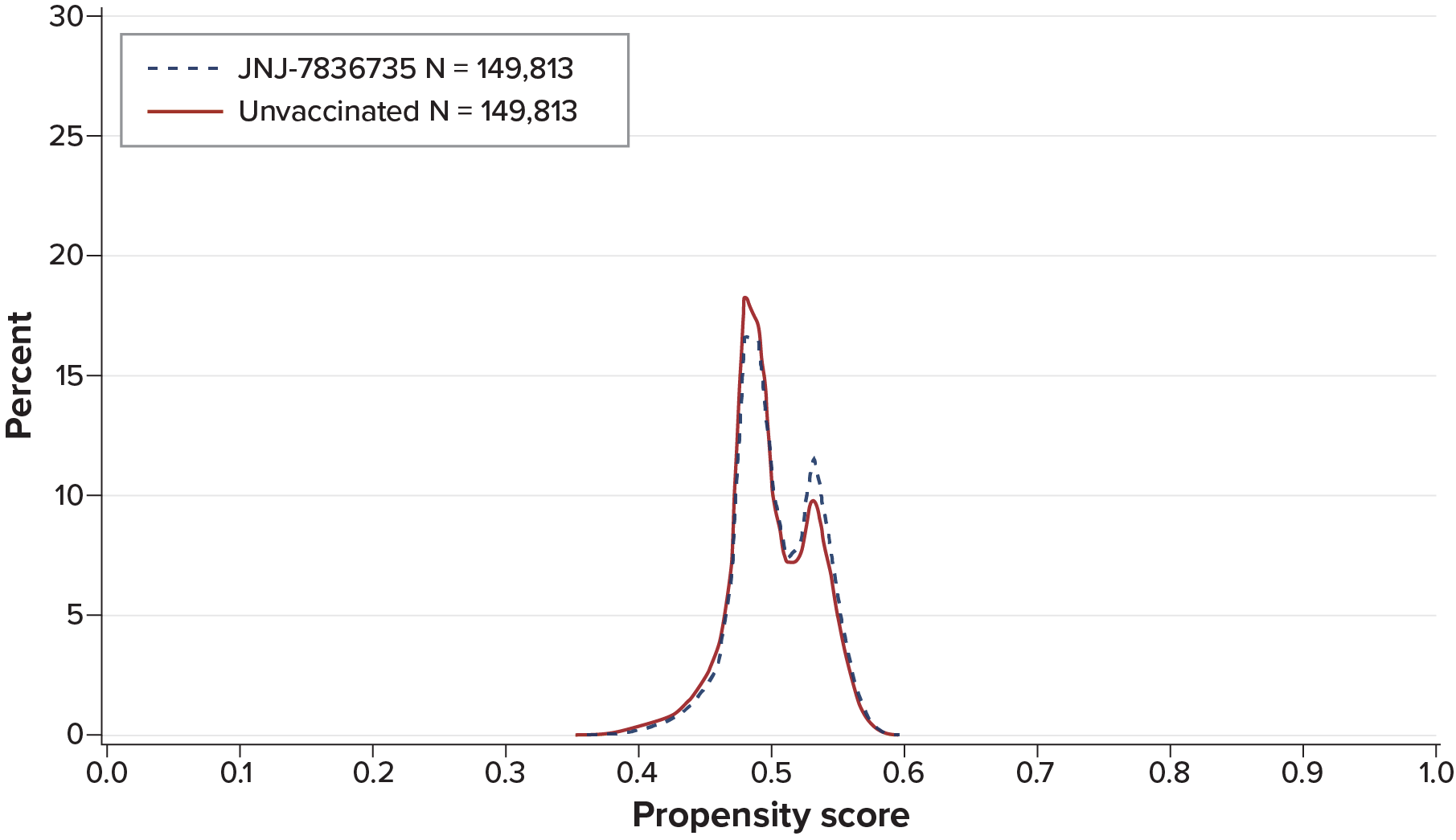


Note: Propensity score variables included: age (categorical with indicator variables:18-24, 25-29, 30-34, 35-39, 40-44, 45-49, 50-54, 55-59, 60-64 years); ambulance use of life support services indicator; arthritis indicator; autoimmune disorders indicator; bone mineral density test indicator; cancer indicator; encounter for cancer screening indicator; chronic kidney disease or renal disease indicator; chronic liver disease indicator; colonoscopy indicator; chronic lung diseases (e.g., asthma, COPD, cystic fibrosis, pulmonary embolism) indicator; county Federal Information Processing System (FIPS) codes (categorical with indicator variables); pregnant at time 0 indicator; COVID-19 diagnoses in any setting indicator; hospitalization or emergency department-diagnosed COVID-19 indicator; at least 1 COVID-19 laboratory performed indicator, COVID-19 diagnoses occurring outside of hospital or ED indicator; COVID-19 vaccination after 31 May 2021 * 1 or more COVID-19 laboratory tests performed (interaction term); COVID-19 vaccination index date in the Delta/Omicron era indicator; diabetes mellitus, type 1 or 2 indicator; Down syndrome indicator; ED visits (categorical with indicator variables: 2+, 1, reference = 0); eye examination indicator; influenza vaccination indicator; heart conditions (e.g., heart failure, coronary artery disease, arrhythmias) indicator; overall binary indicator of the presence of any of these conditions will be defined to identify individuals who may qualify for priority groups for vaccination eligibility; hypertension indicator; hospitalizations (categorical with indicator variables: 2+, 1, reference = 0); lipid abnormality indicator; mental health conditions indicator; dementia or other neurological conditions indicator; obese or severely obese indicator; pneumococcal vaccination indicator; female indicator; male indicator; sickle cell disease or thalassemia indicator; skilled nursing facility stay indicator; IIS jurisdiction (categorical with indicator variables); stroke or cerebrovascular disease indicator; tuberculosis indicator; immunocompromised state indicator; pregnancy completion before time 0 indicator; weakness indicator; well-check/well-child preventive healthcare visit indicator.

COPD = chronic obstructive pulmonary disease; COVID-19 = coronavirus disease 2019; ED = emergency department; IIS = immunization information system.

1. Estimated Effectiveness of Receiving a Complete Primary Series of COVID-19 Vaccine in Adults Aged 18-64 Years, Compared With Being Unvaccinated, Overall and Within Subgroups


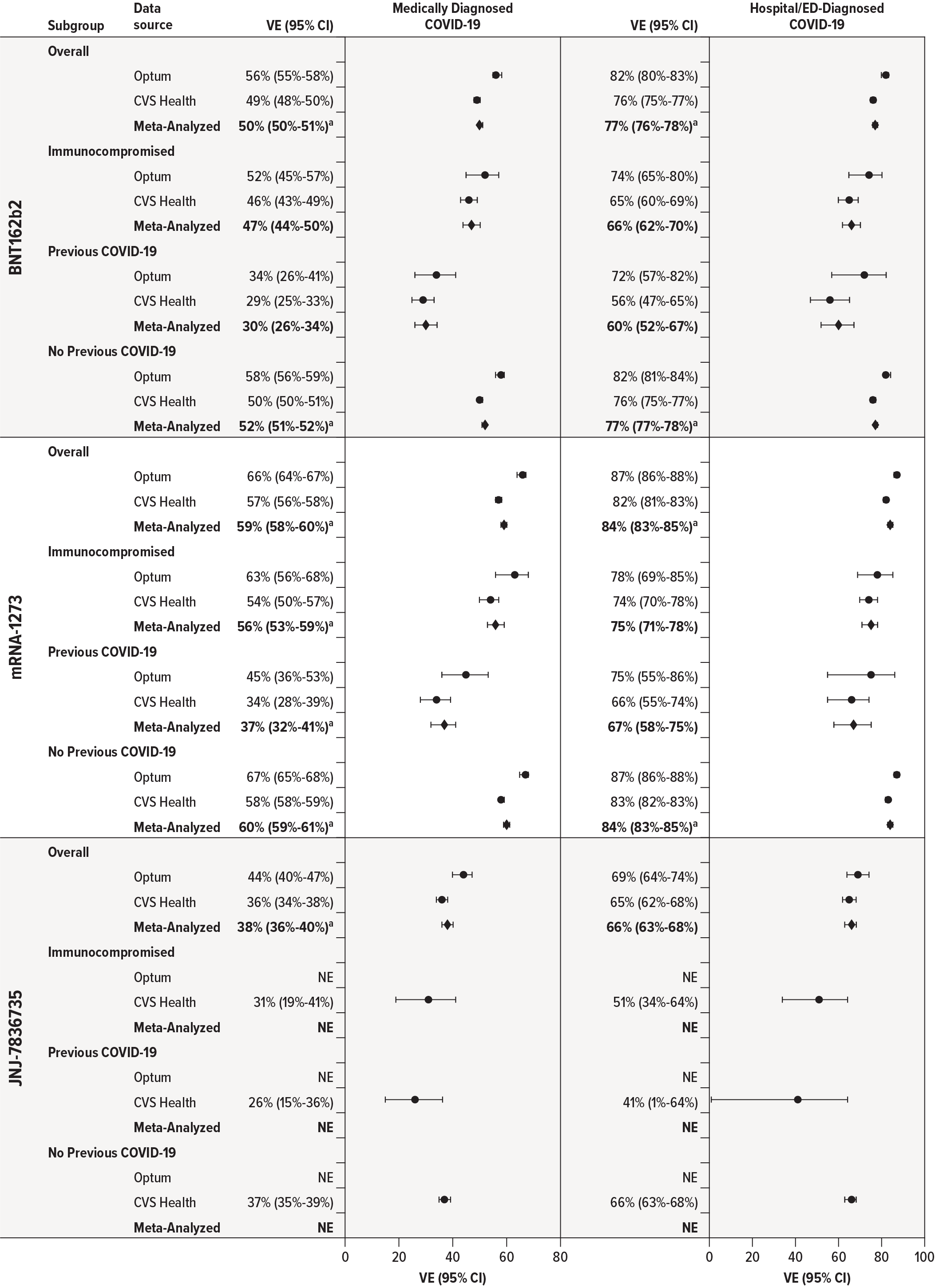


CI = confidence interval; COVID-19 = coronavirus disease 2019; ED = emergency department; VE = vaccine effectiveness.

^a^ Indicates evidence of statistical heterogeneity between data source–specific estimates, *p* < 0.05.

1. Estimated Effectiveness of Receiving a Complete Primary Series of COVID-19 Vaccine in Adults Aged 18-64 Years, Compared With Being Unvaccinated, Primary and Sensitivity Analyses

**
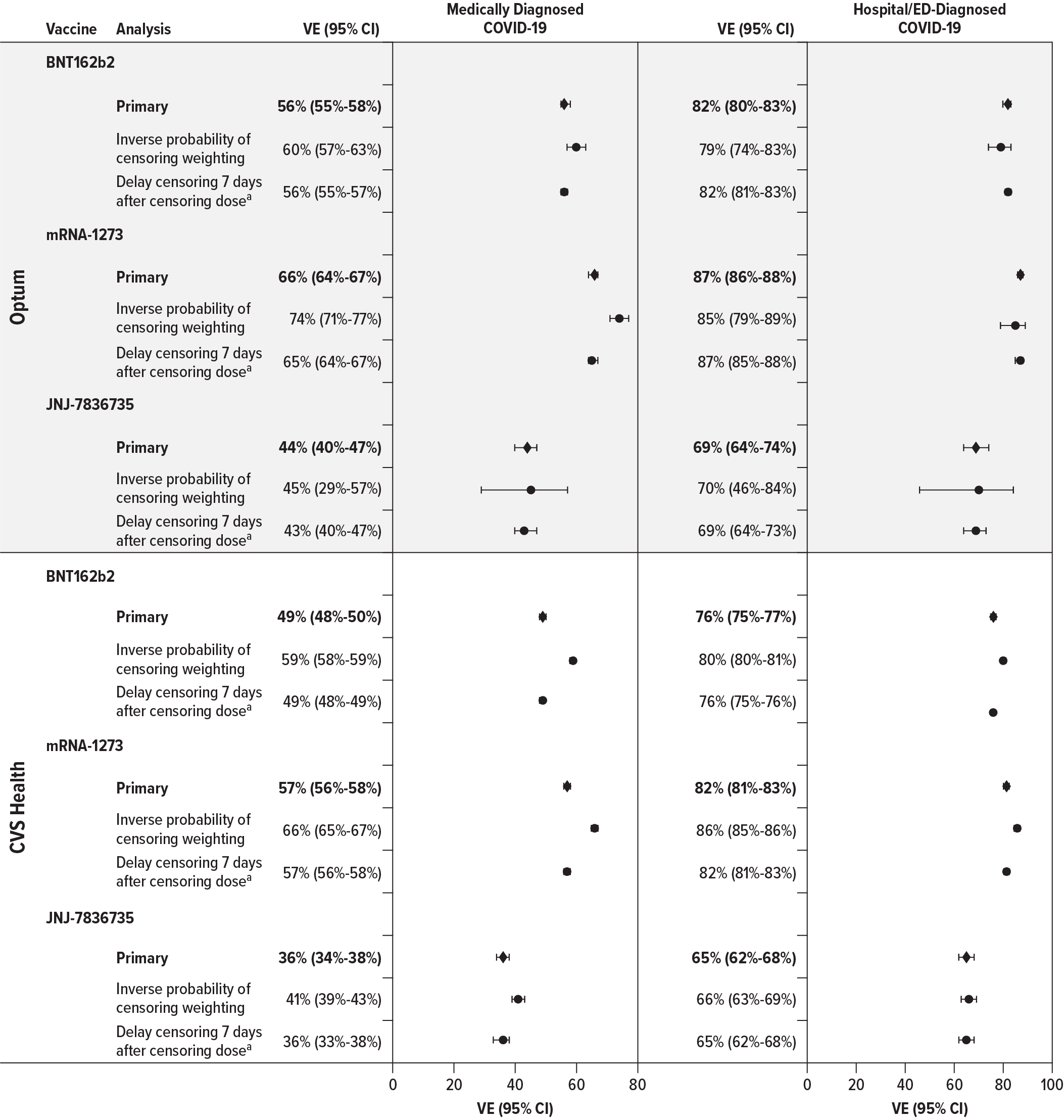
**

CI = confidence interval; COVID-19 = coronavirus disease 2019; ED = emergency department; VE = vaccine effectiveness.

^a^ Censoring doses consisted of individuals in the unvaccinated group receiving any vaccine, or individuals in the vaccinated group receiving Dose 2 too early, Dose 2 of a different brand, or receiving a third dose.

1. Weighted Cumulative Incidence of COVID‑19 Outcomes in Adults Aged 18-64 Years Receiving a Complete Primary Series of COVID-19 Vaccines and Matched Unvaccinated Comparators

A. BNT162b2 vs. Unvaccinated Comparators, Optum

i. Medically Diagnosed COVID-19


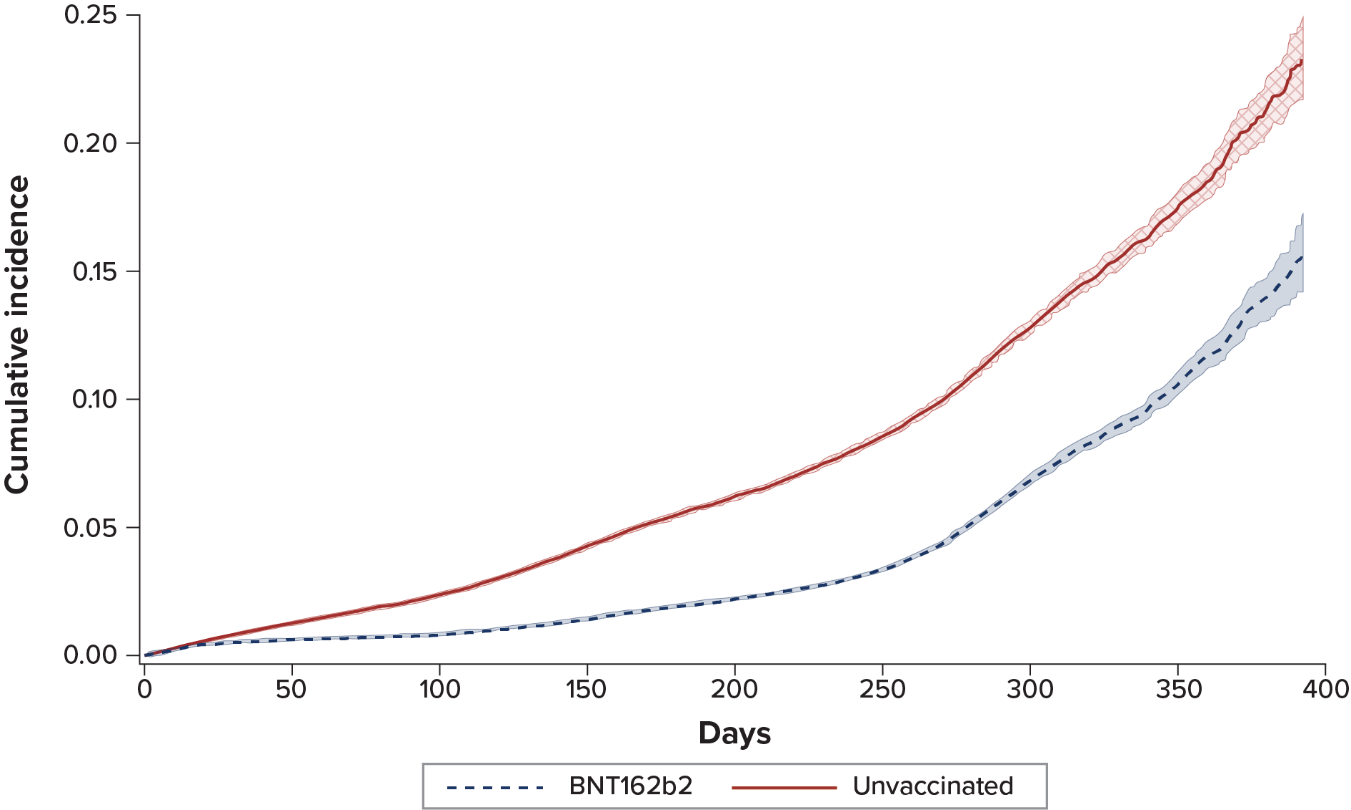


ii. Hospital/ED-Diagnosed COVID-19


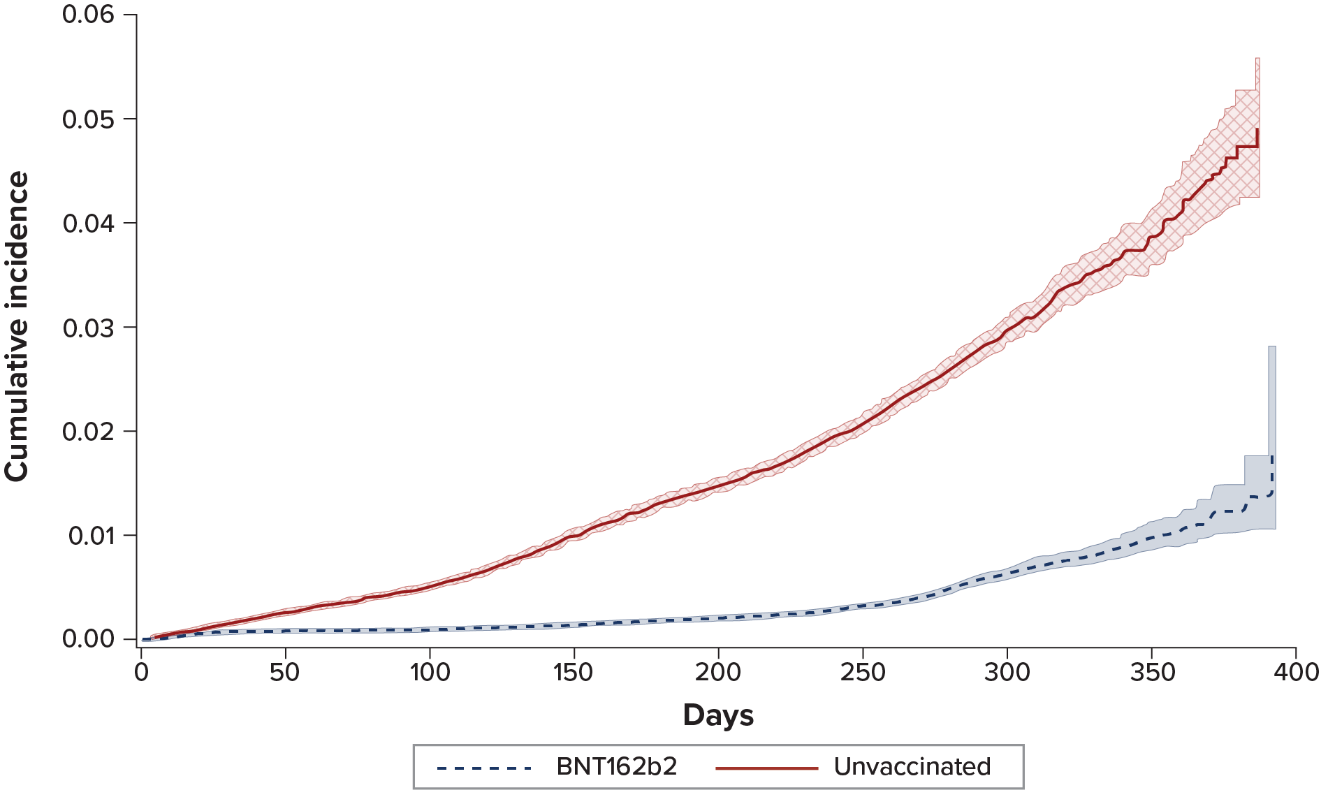


B. mRNA-1273 vs. Unvaccinated Comparators, Optum

i. Medically Diagnosed COVID-19


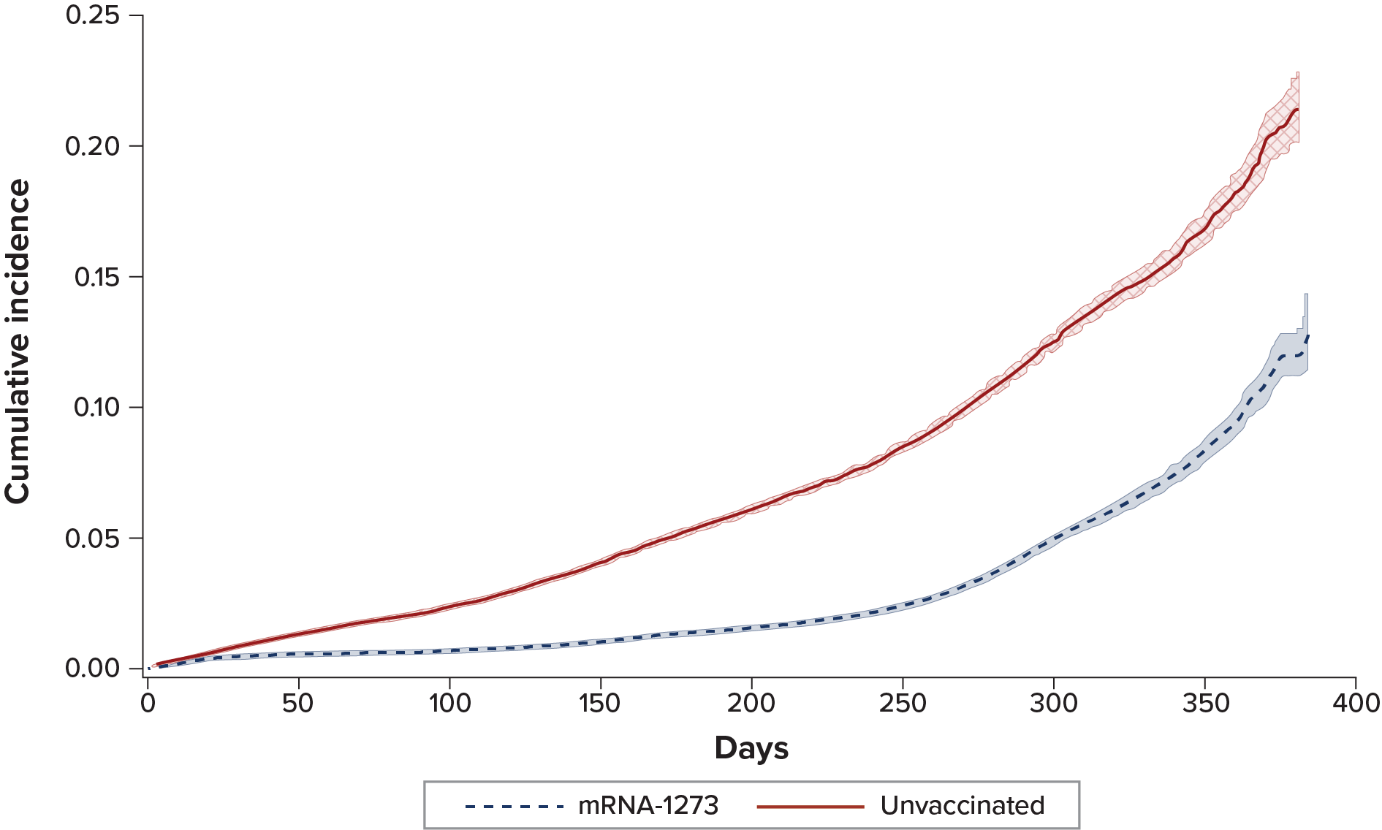


ii. Hospital/ED-Diagnosed COVID-19


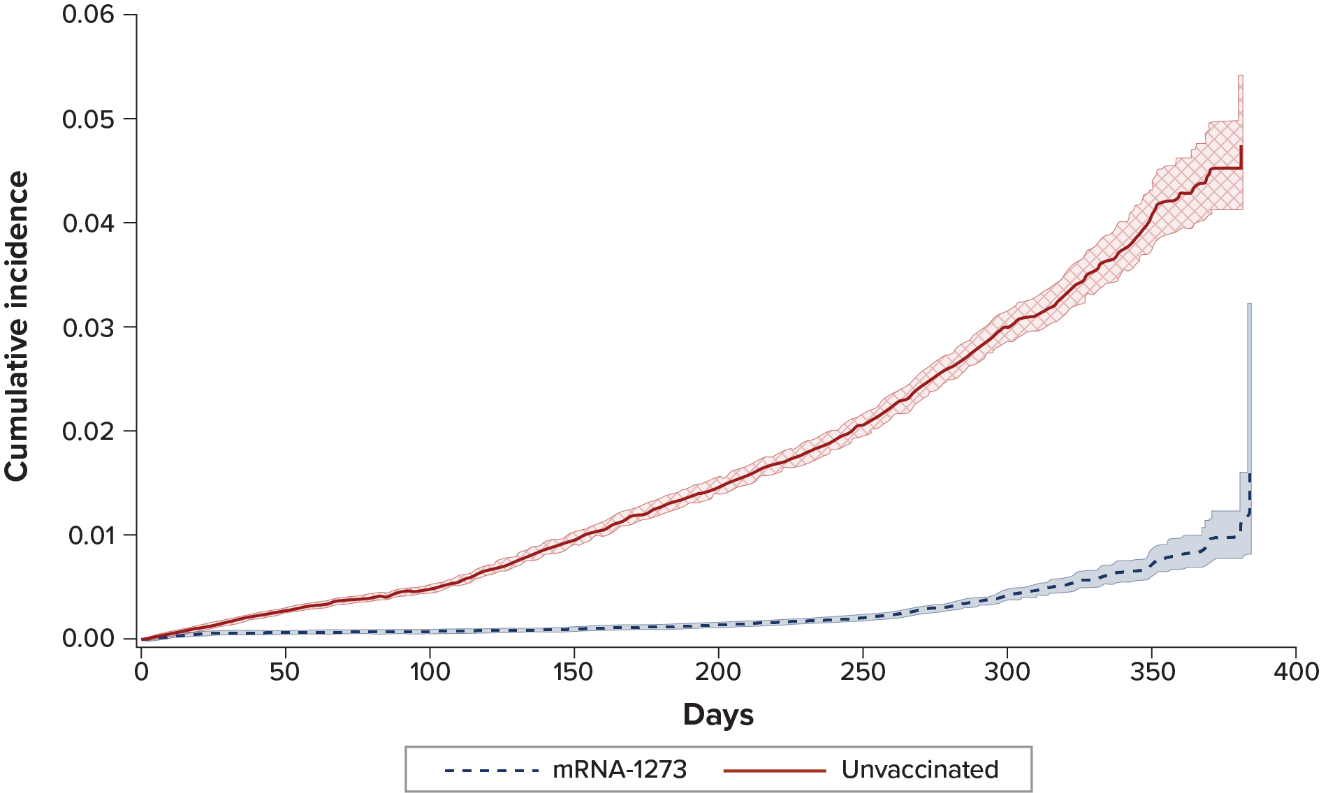


C. JNJ-7836735 vs. Unvaccinated Comparators, Optum

i. Medically Diagnosed COVID-19


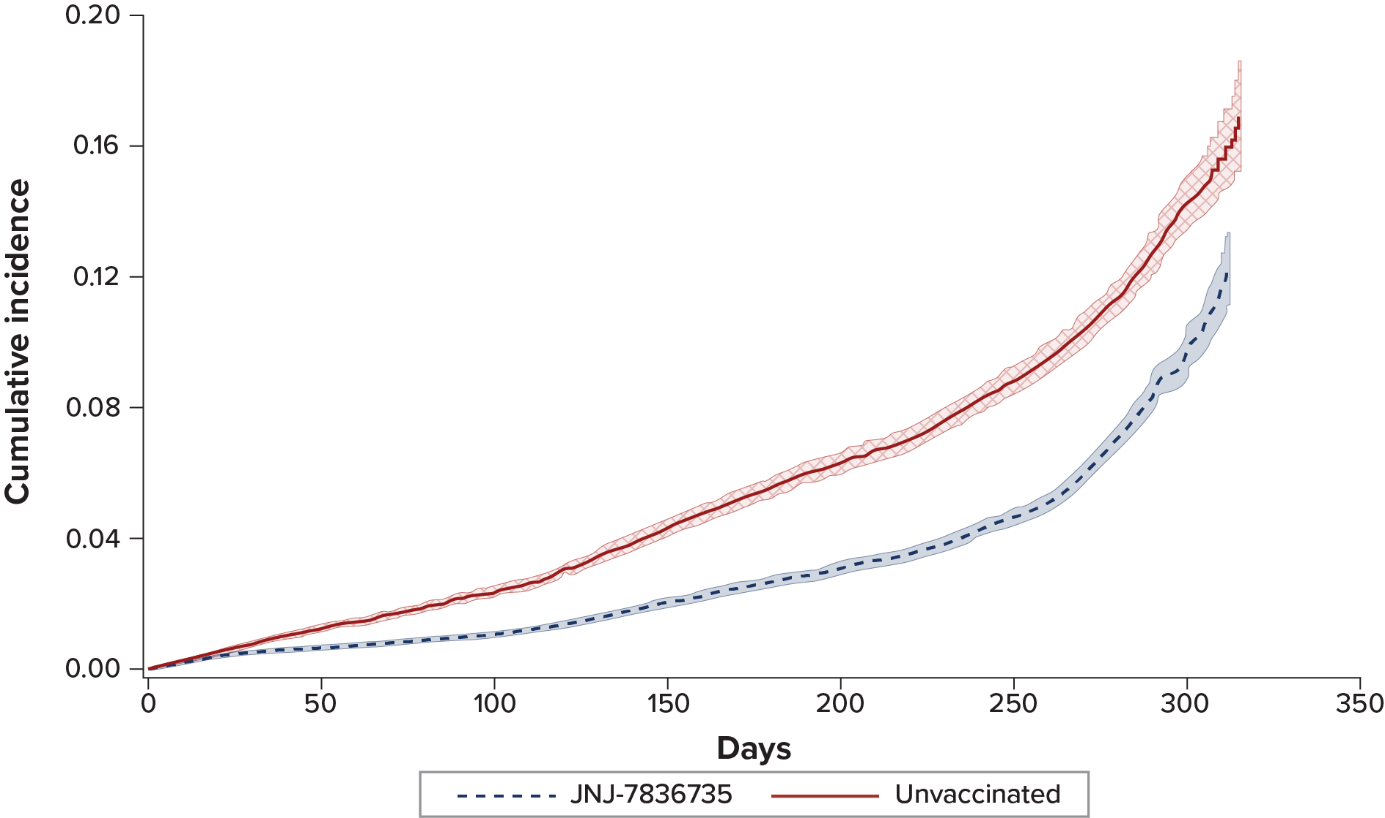


ii. Hospital/ED-Diagnosed COVID-19


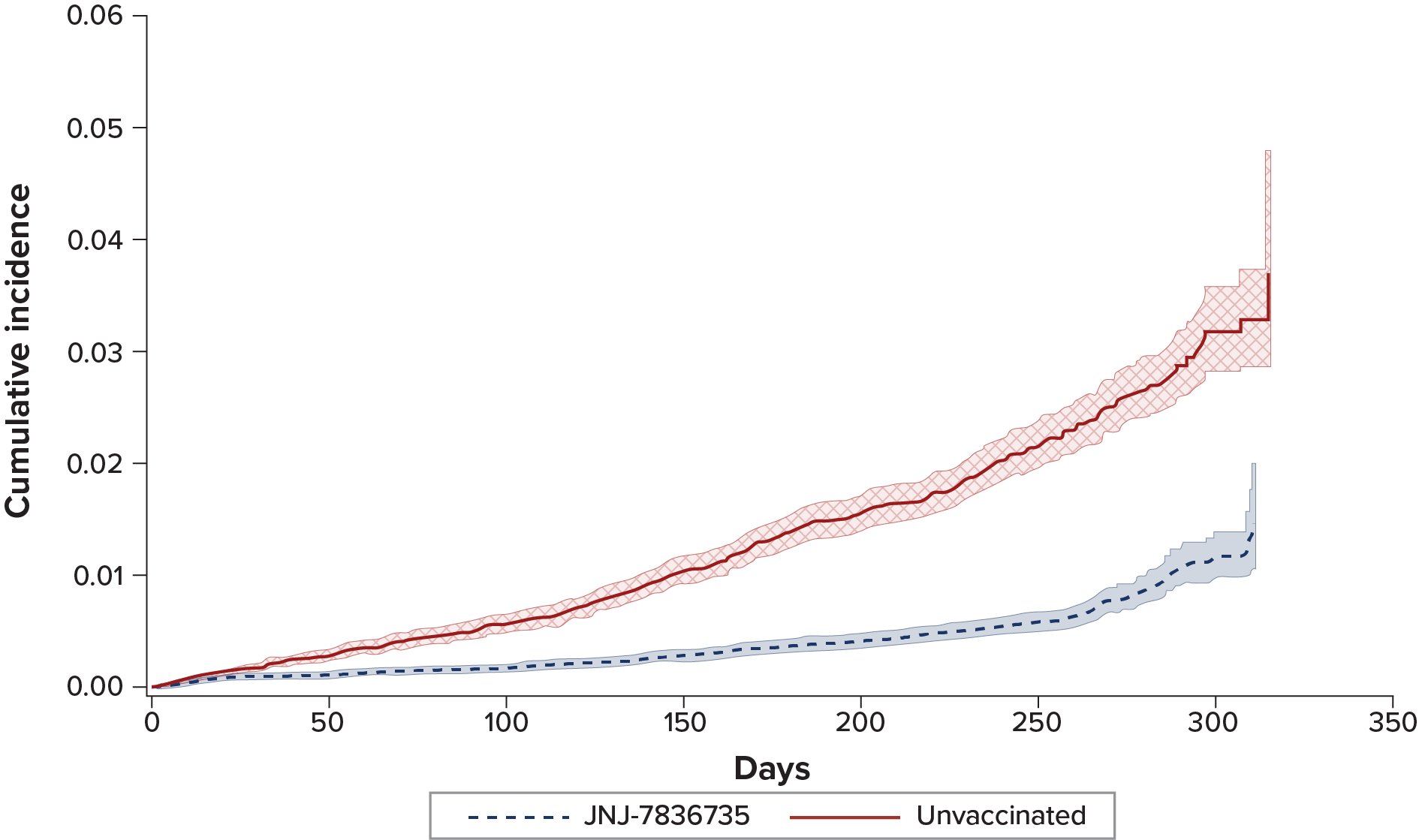


D. BNT162b2 vs. Unvaccinated Comparators, CVS Health

i. Medically Diagnosed COVID-19


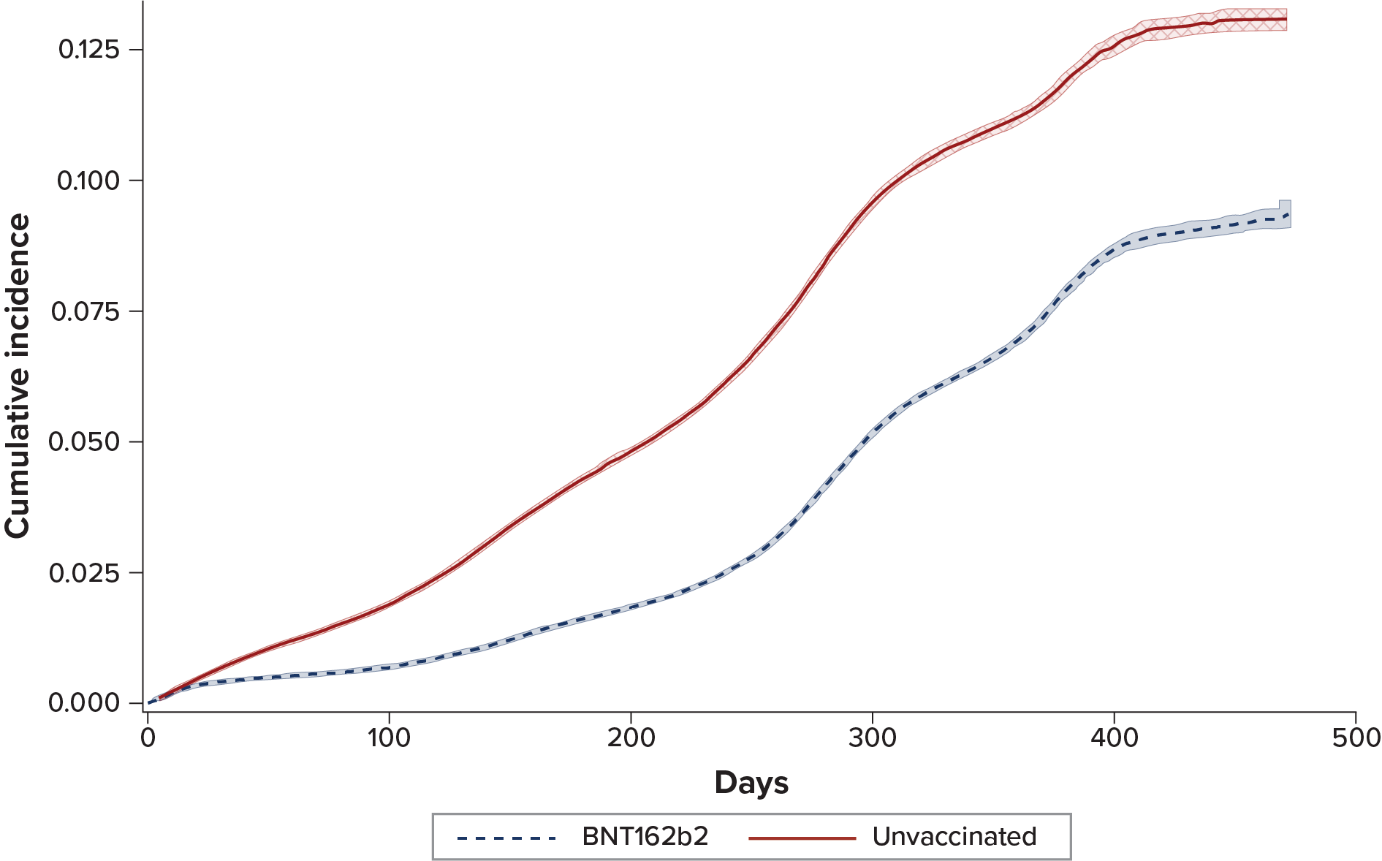


ii. Hospital/ED-Diagnosed COVID-19


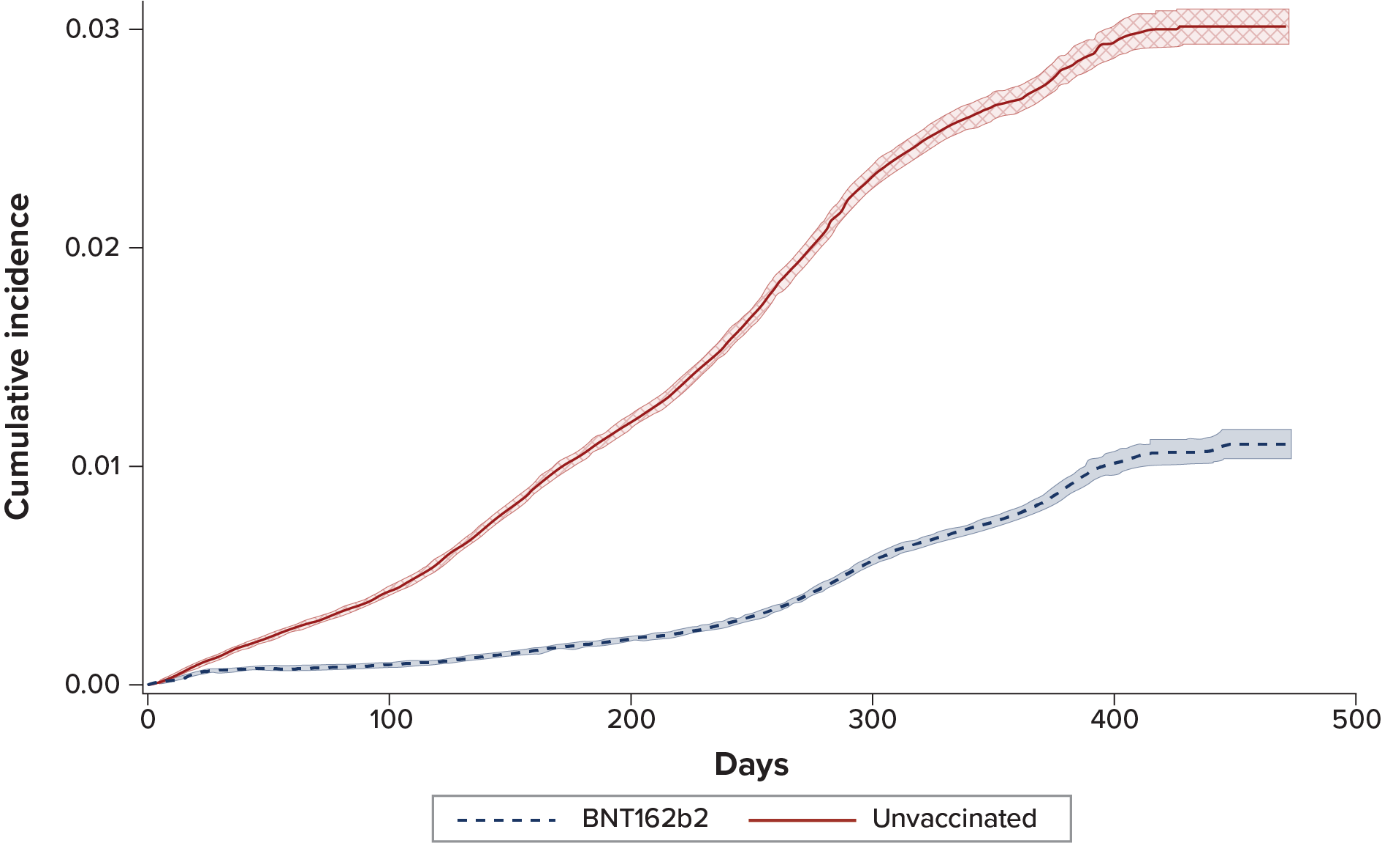


E. mRNA-1273 vs. Unvaccinated Comparators, CVS Health

i. Medically Diagnosed COVID-19


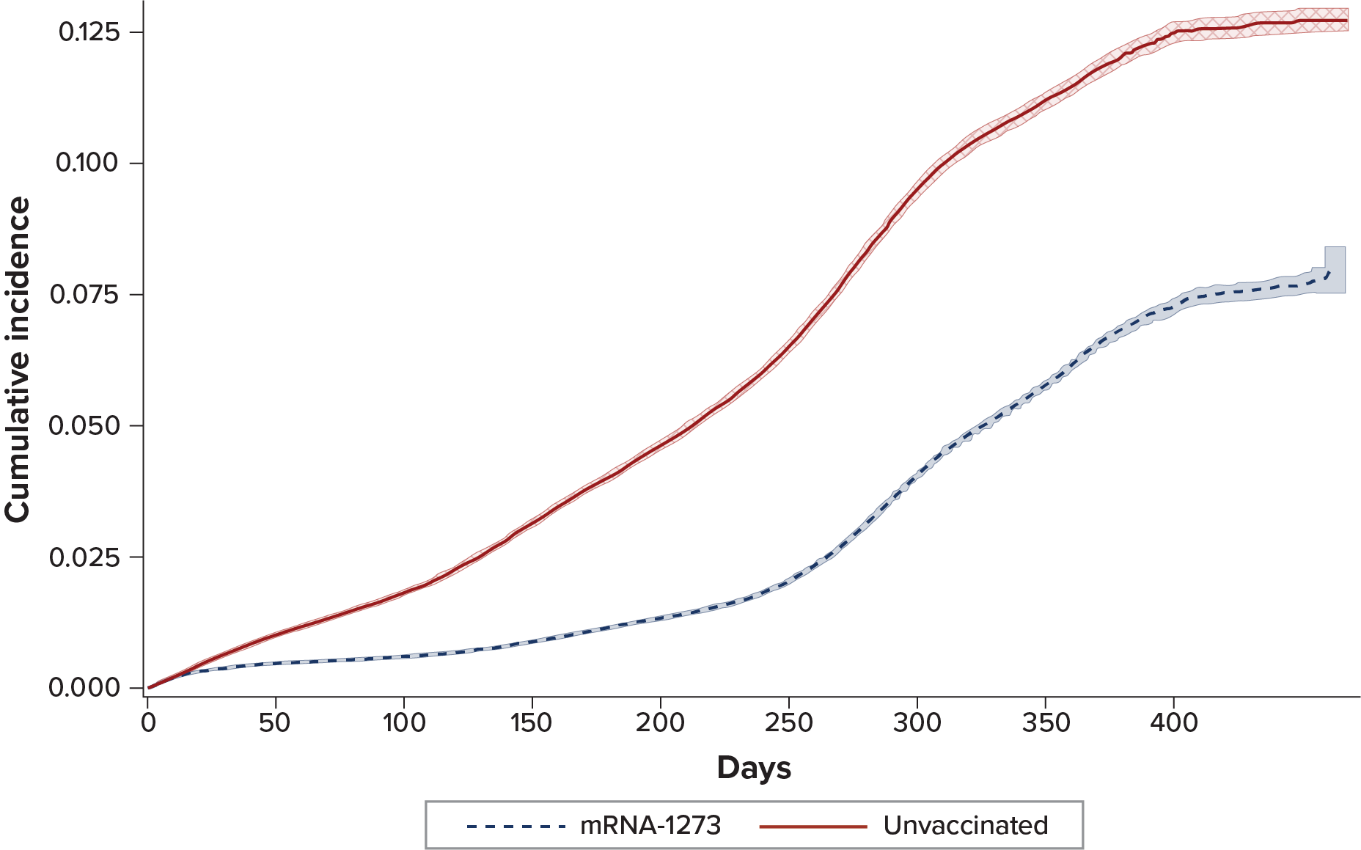


ii. Hospital/ED-Diagnosed COVID-19


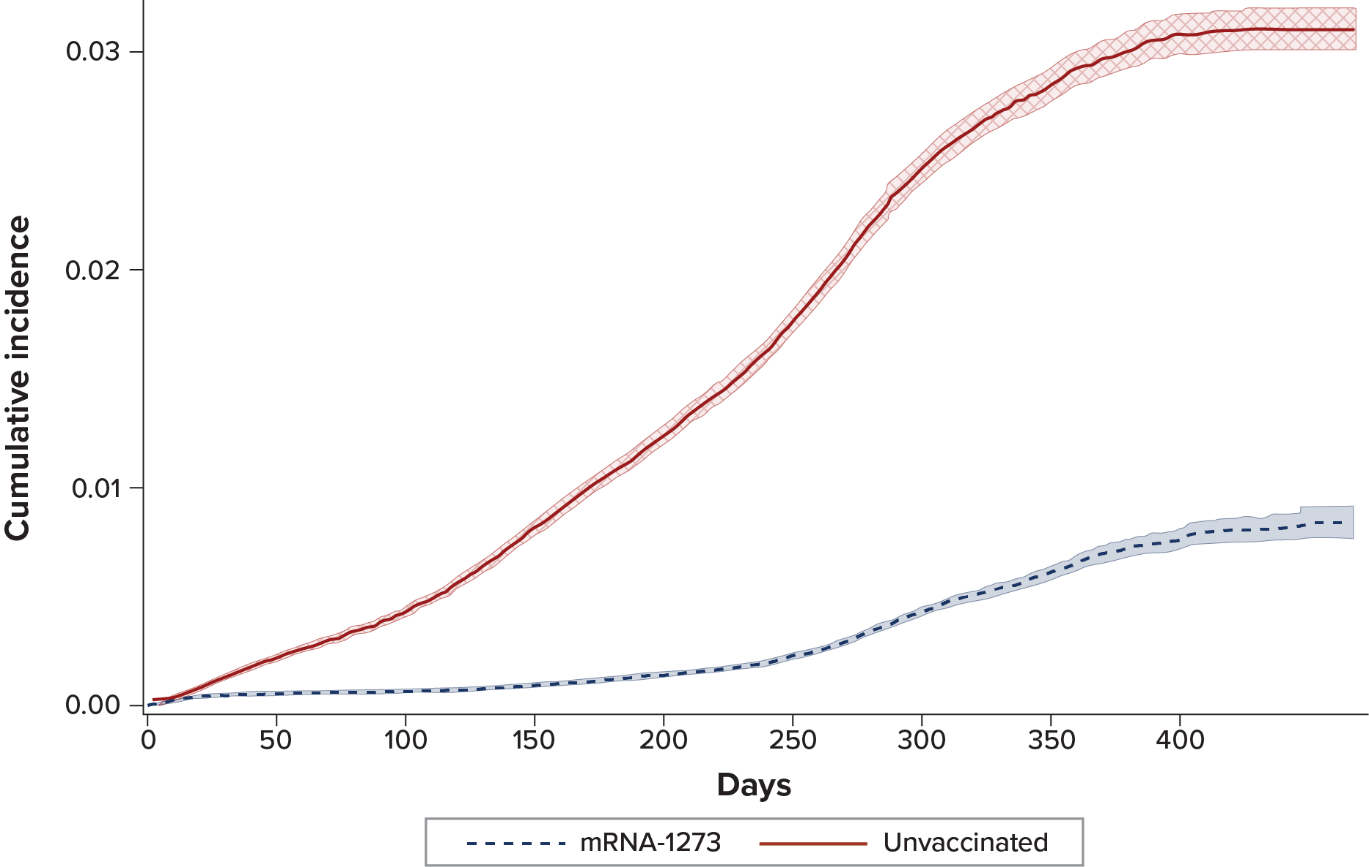


F. JNJ-7836735 vs. Unvaccinated Comparators, CVS Health

i. Medically Diagnosed COVID-19


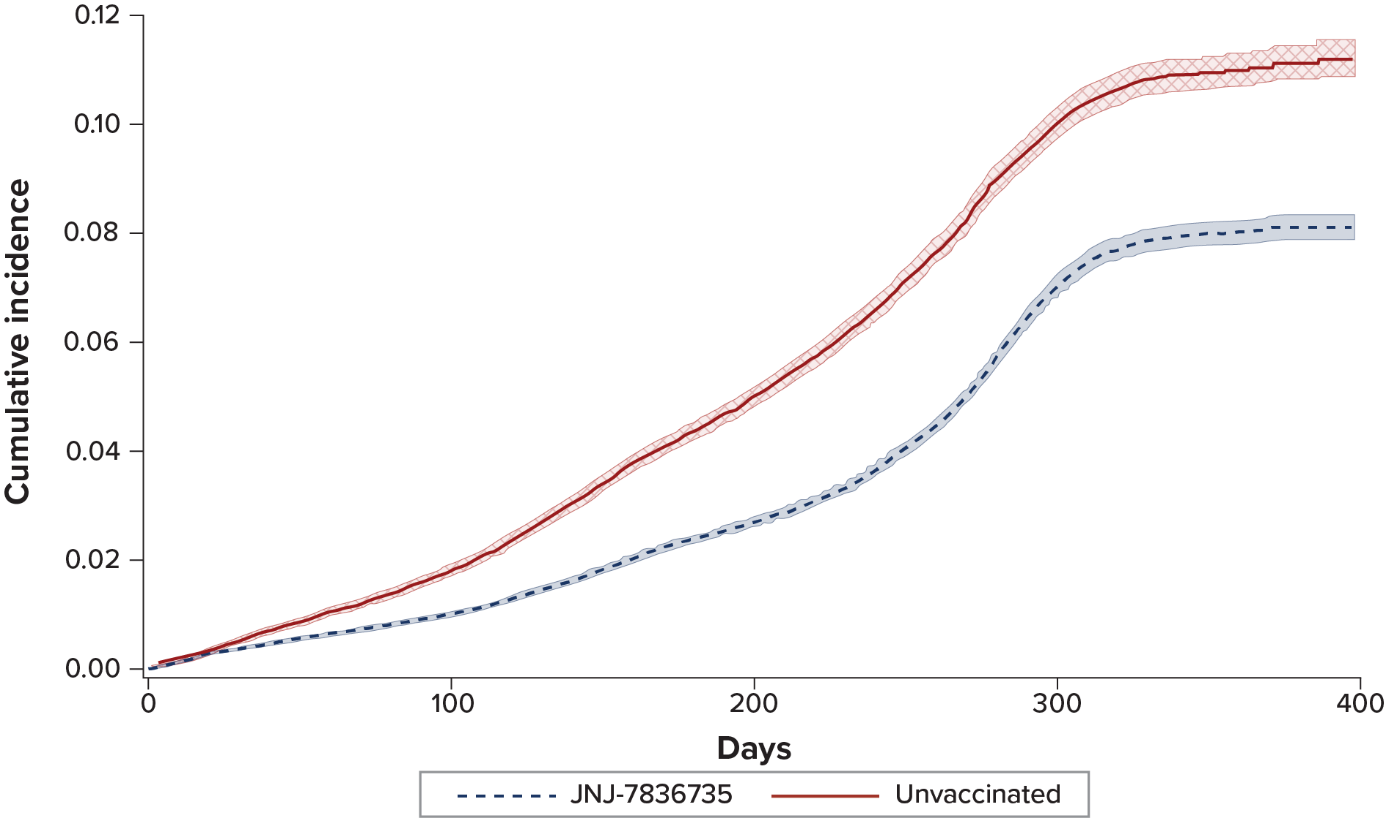


ii. Hospital/ED-Diagnosed COVID-19


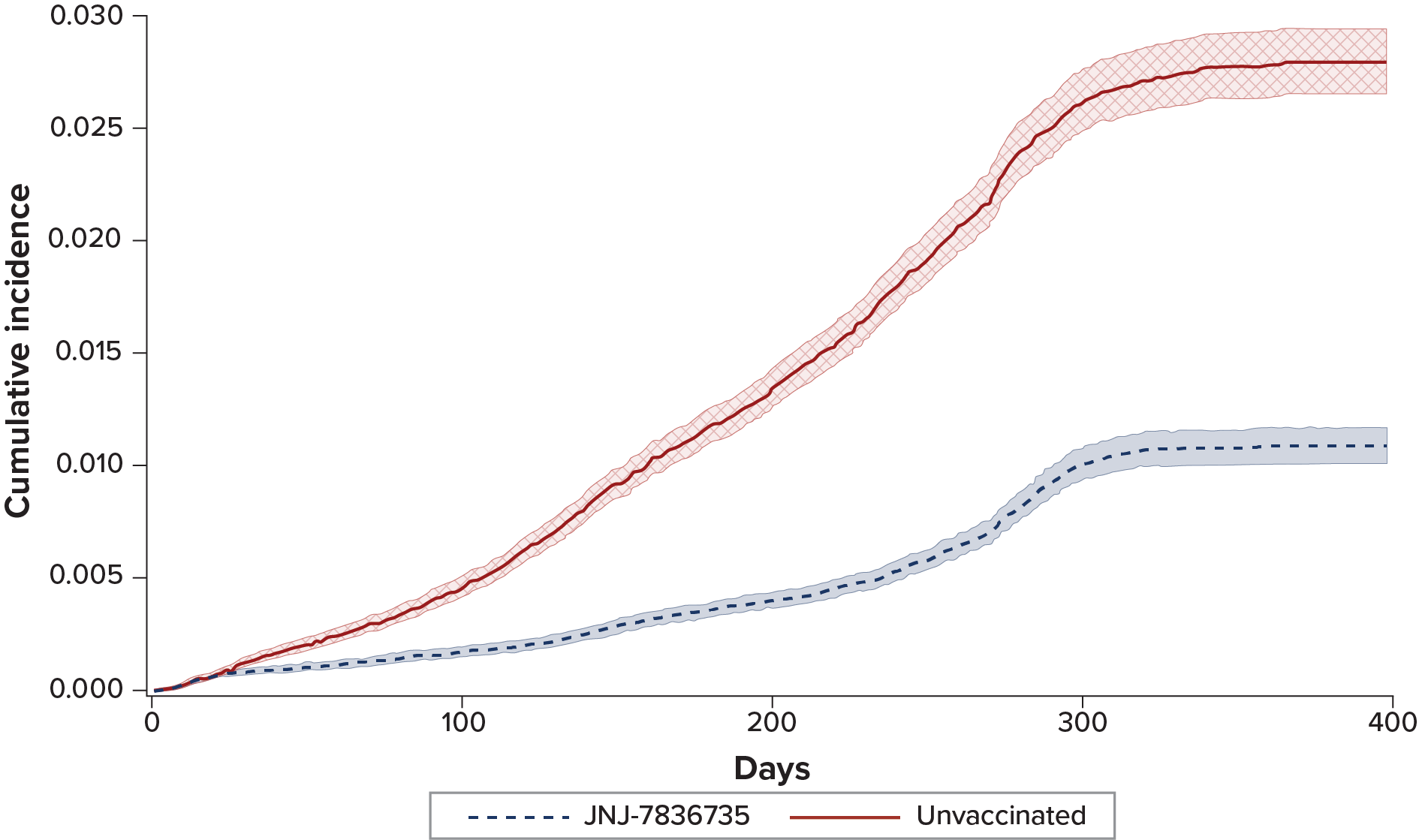


COVID-19 = coronavirus disease 2019.

1. Cumulative Incidence of COVID‑19 Outcomes in Adults Receiving a Complete Primary Series of COVID-19 Vaccines and Matched Unvaccinated Comparators, Negative Control Outcome in the First 14 Days

A. BNT162b2 vs. Unvaccinated Comparators, Optum

i. Medically Diagnosed COVID-19


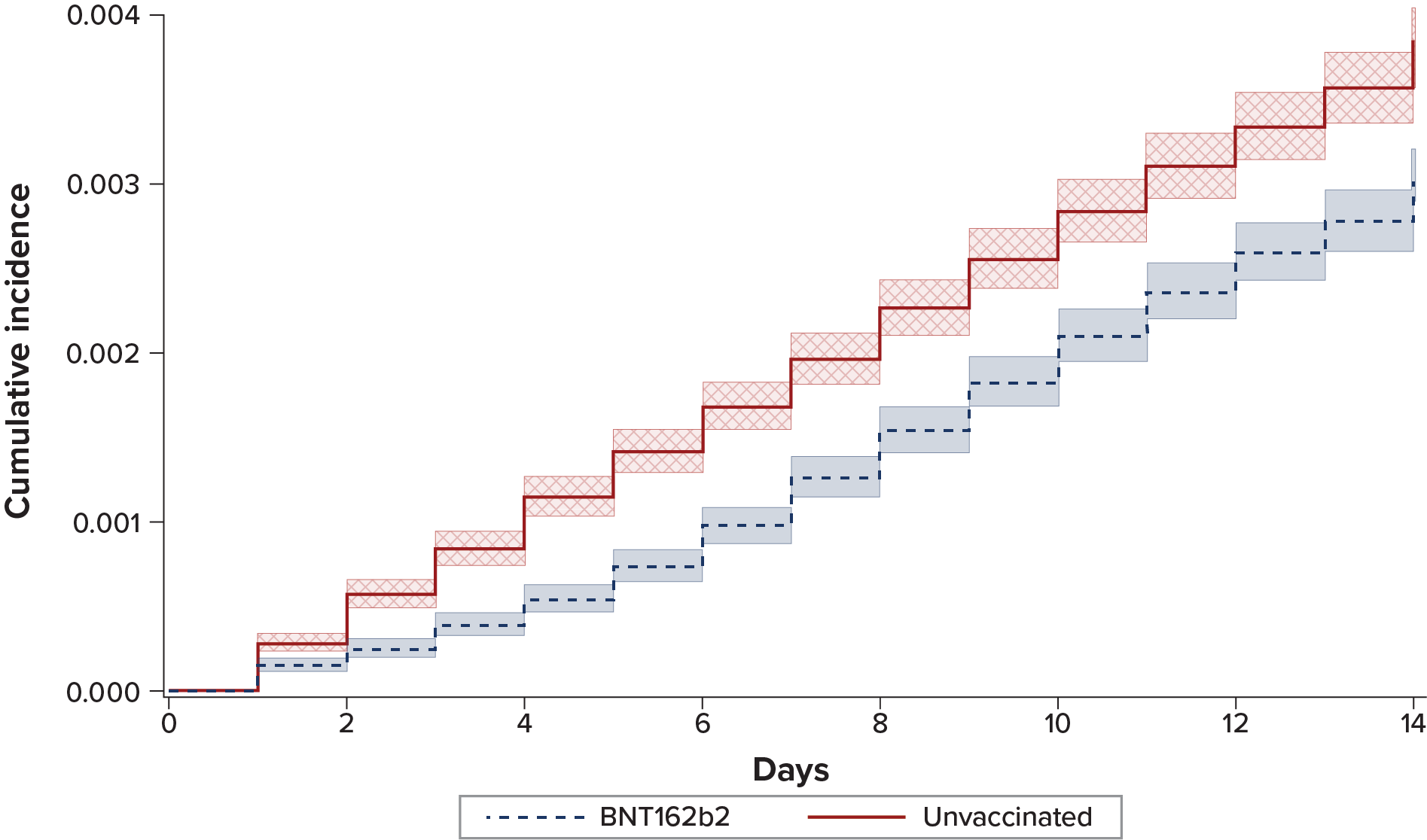


ii. Hospital/ED-Diagnosed COVID-19


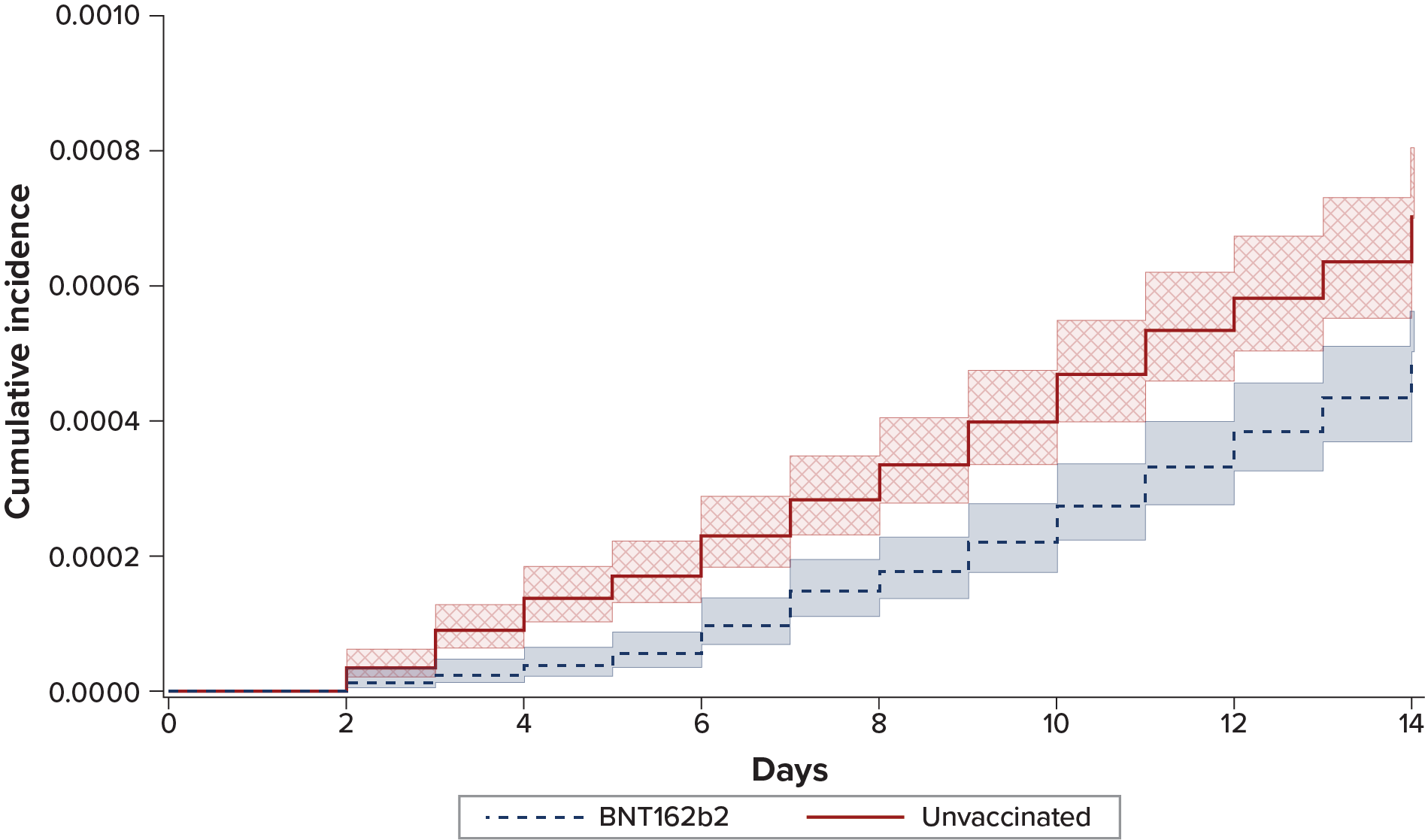


B. mRNA-1273 vs. Unvaccinated Comparators, Optum

i. Medically Diagnosed COVID-19


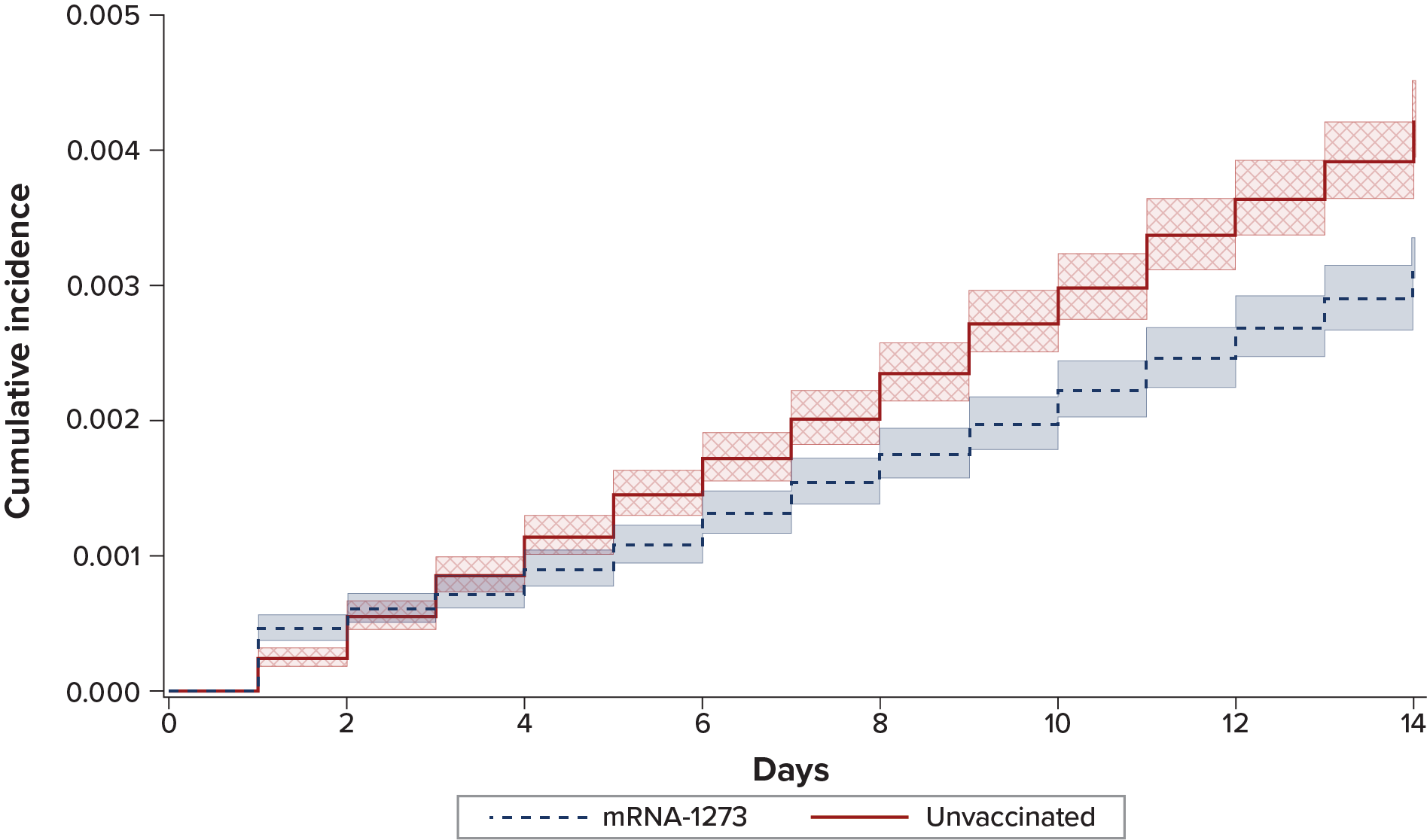


ii. Hospital/ED-Diagnosed COVID-19


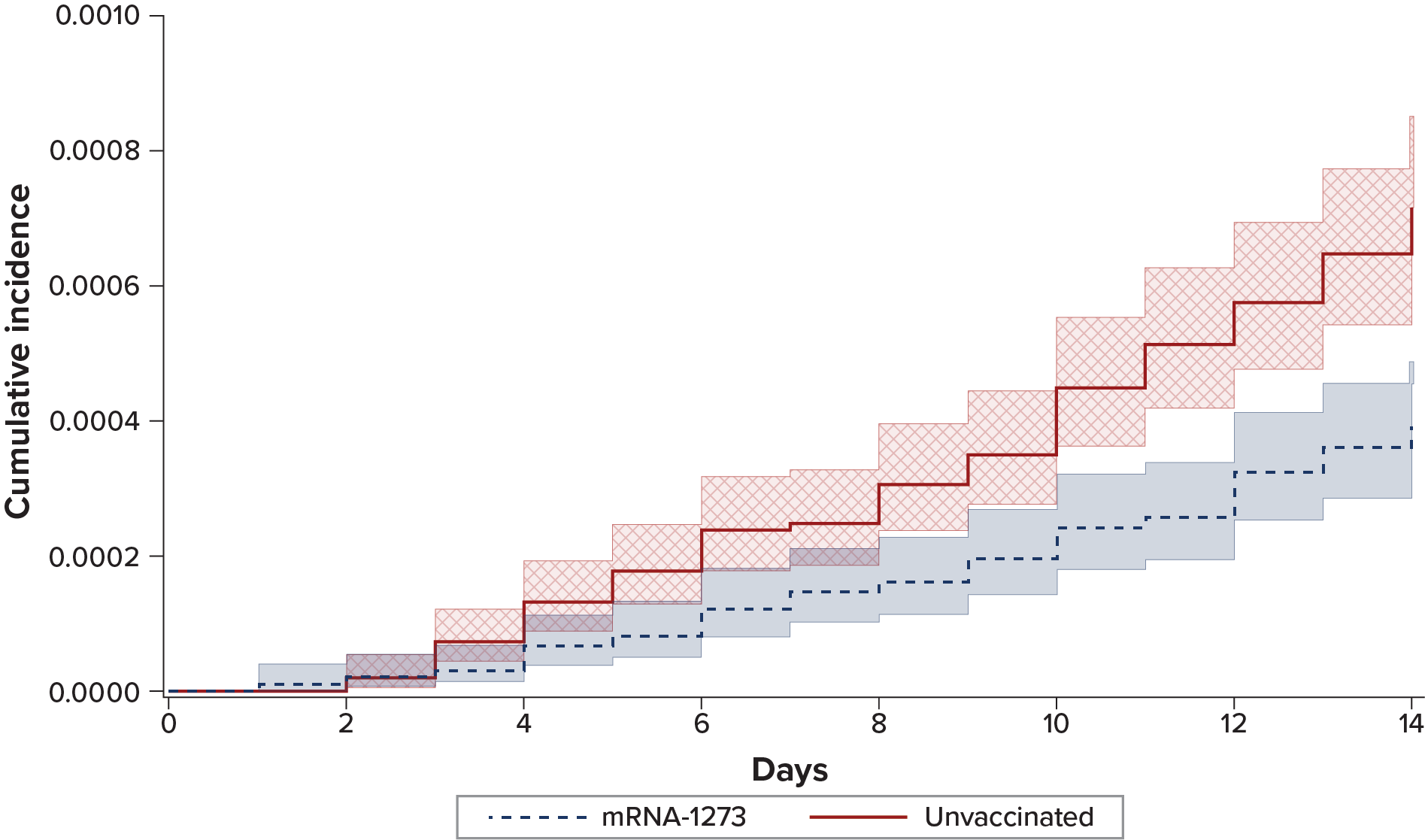


C. JNJ-7836735 vs. Unvaccinated Comparators, Optum

i. Medically Diagnosed COVID-19


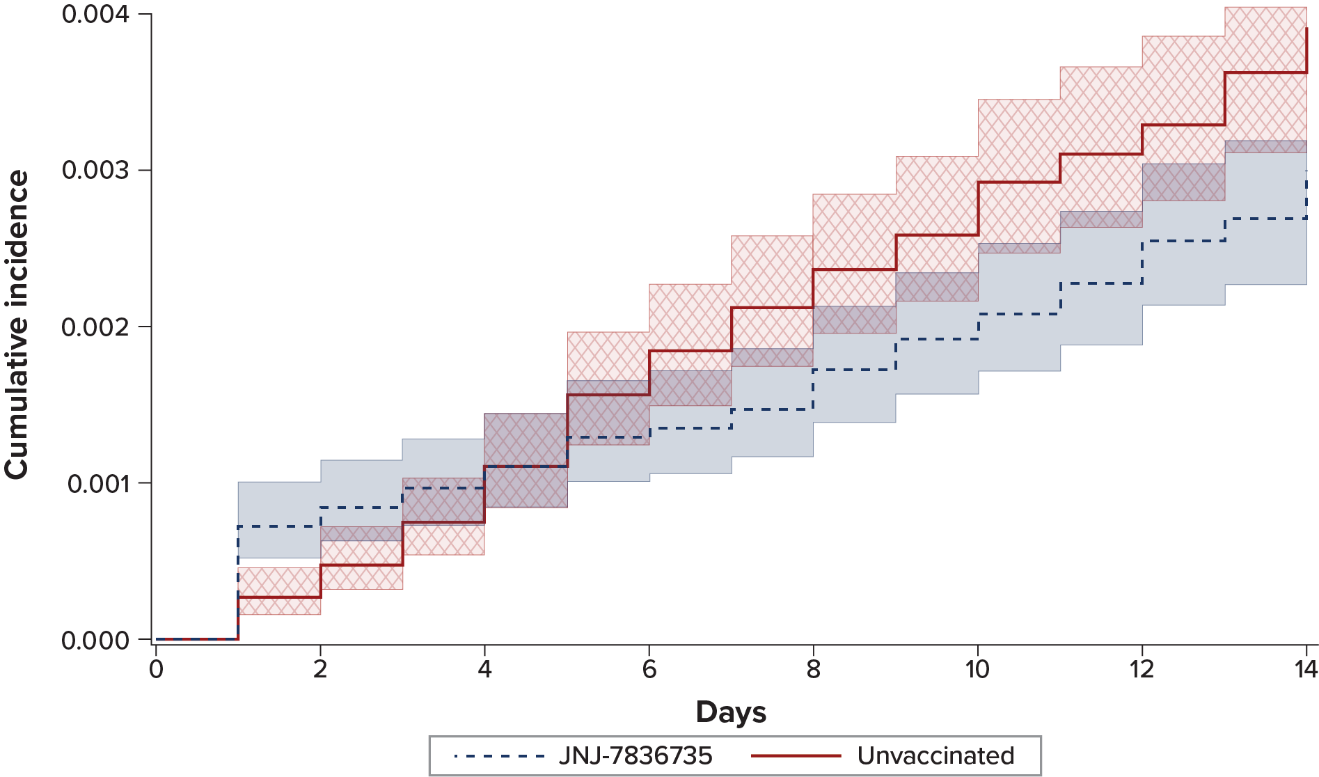


ii. Hospital/ED-Diagnosed COVID-19


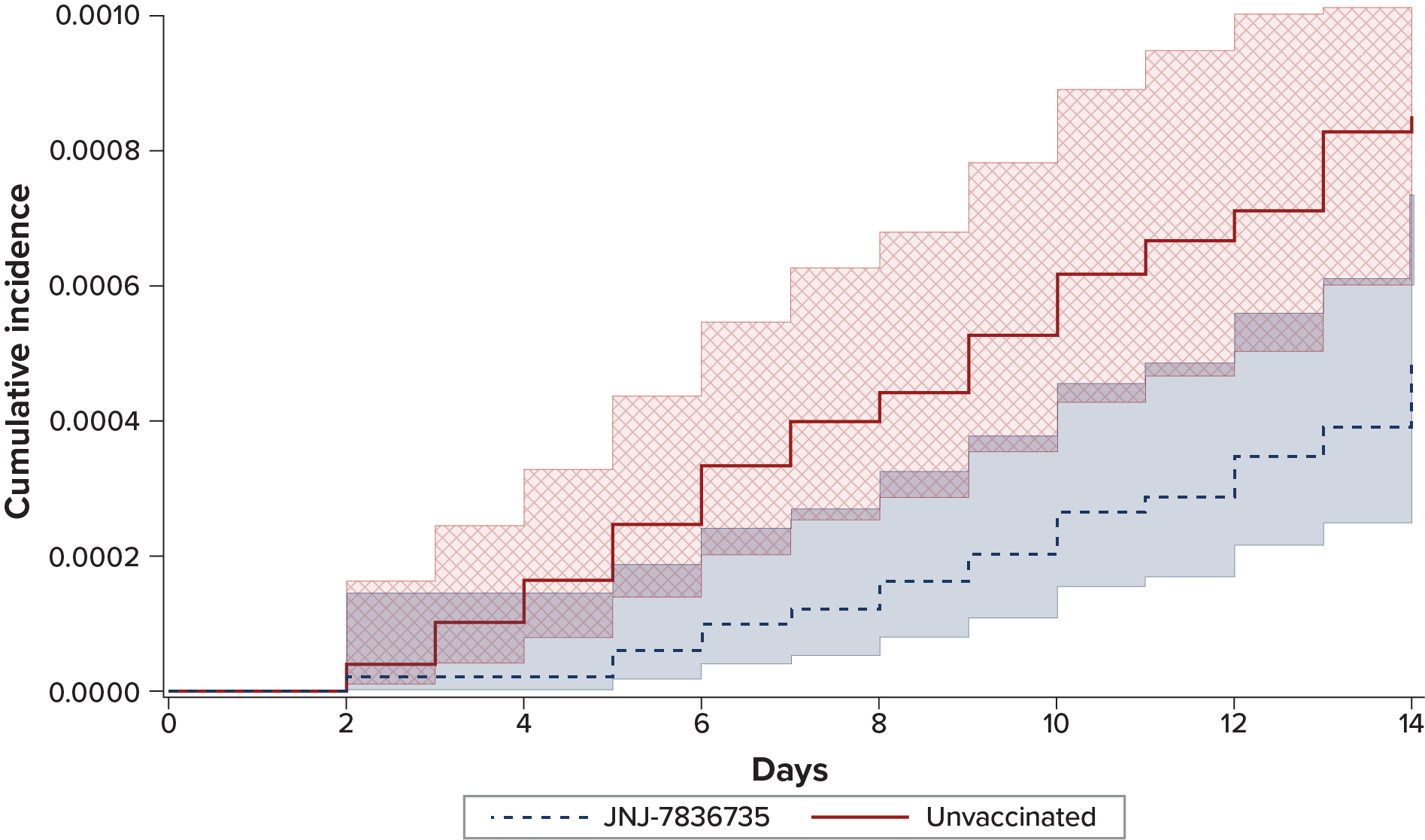


D. BNT162b2 vs. Unvaccinated Comparators, CVS Health

i. Medically Diagnosed COVID-19


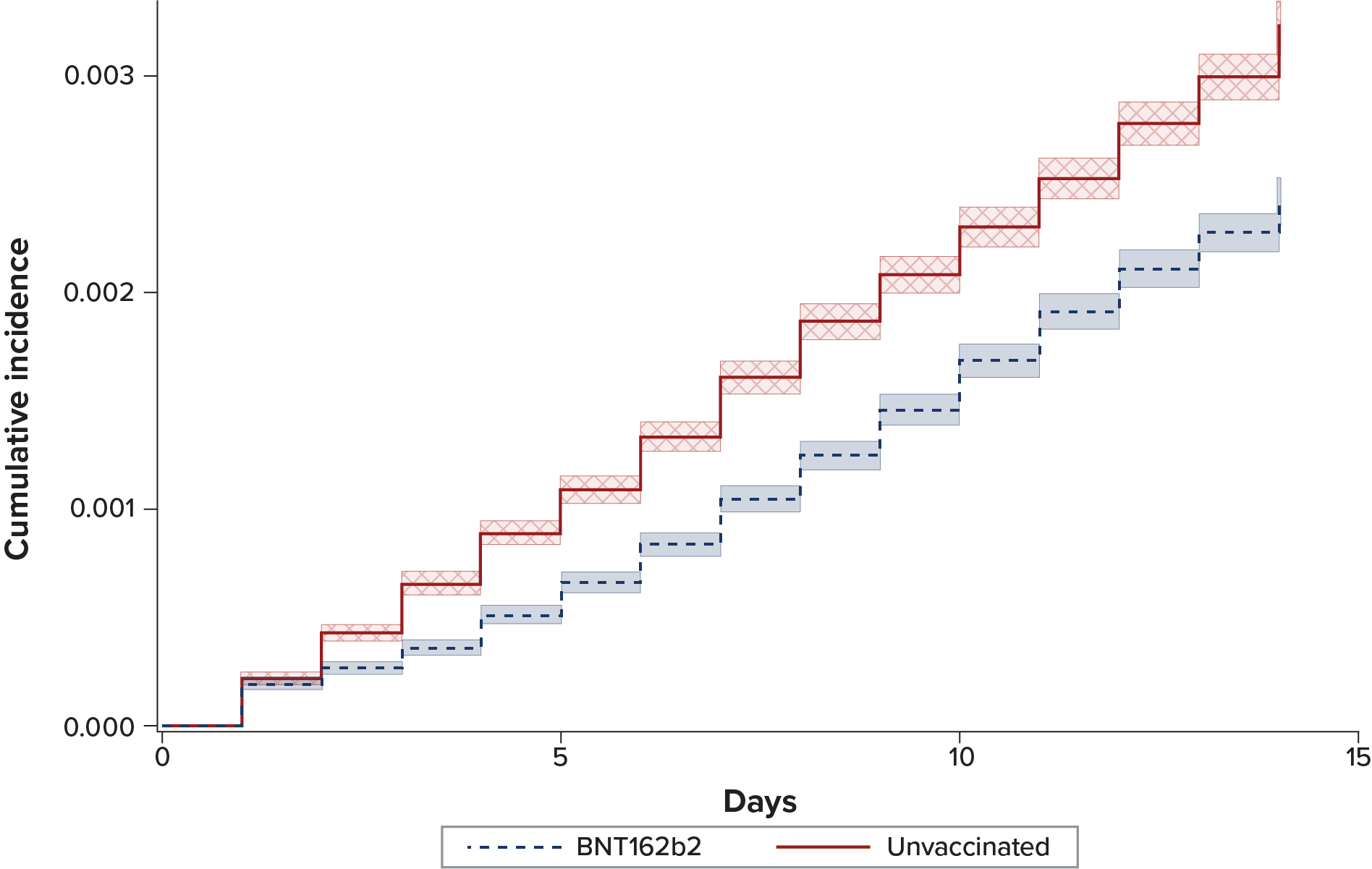


ii. Hospital/ED-Diagnosed COVID-19


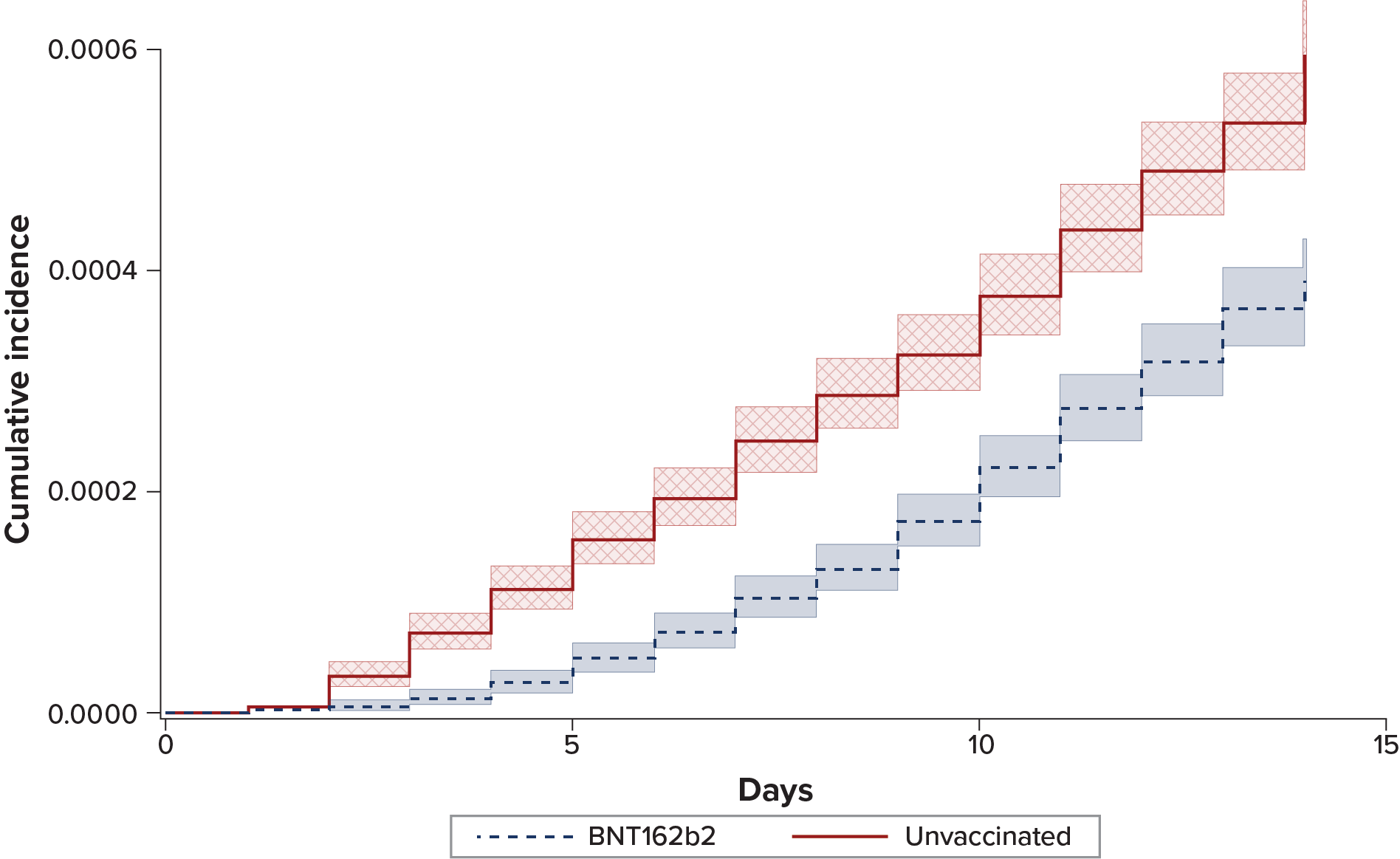


E. mRNA-1273 vs. Unvaccinated Comparators, CVS Health

i. Medically Diagnosed COVID-19


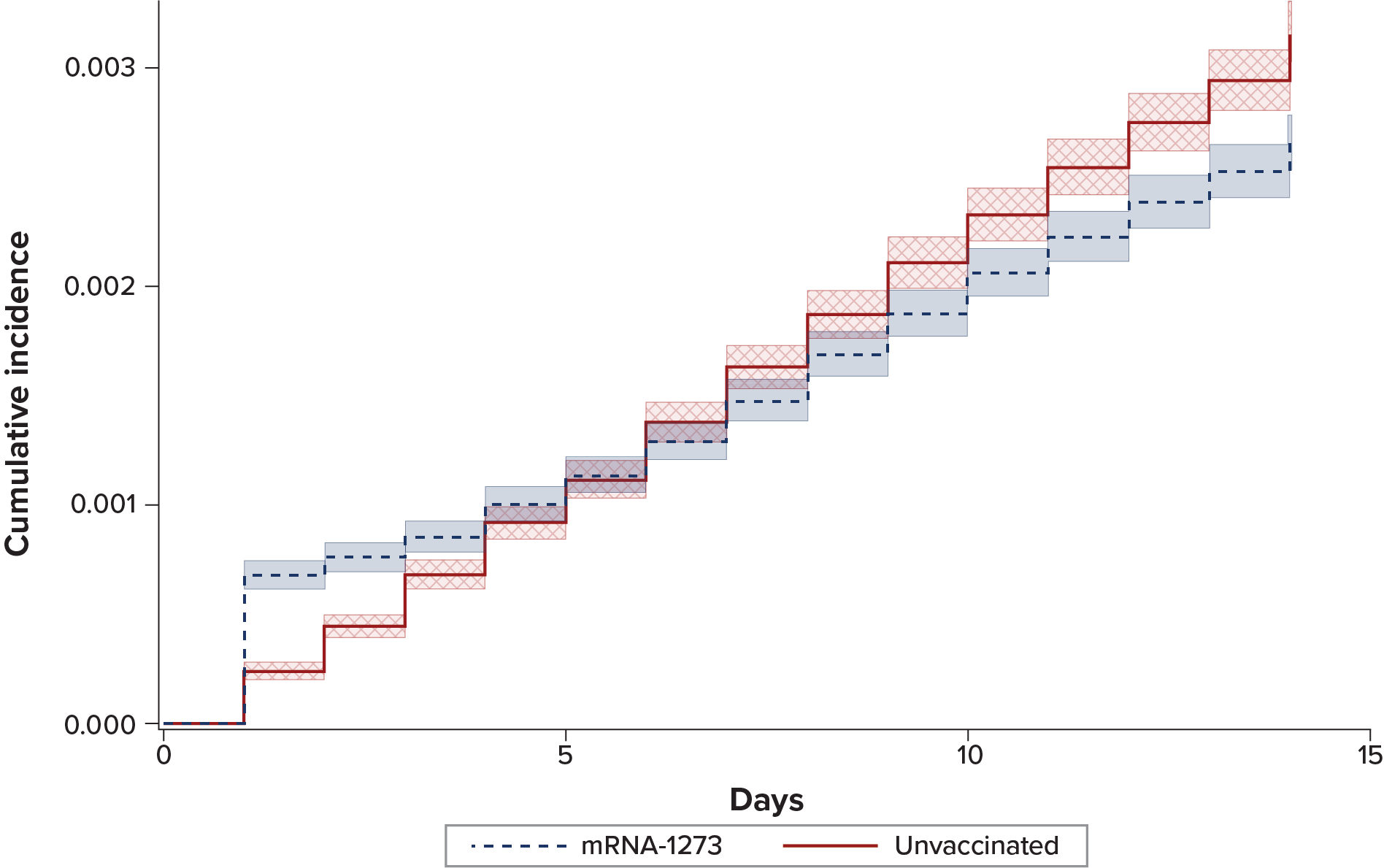


ii. Hospital/ED-Diagnosed COVID-19


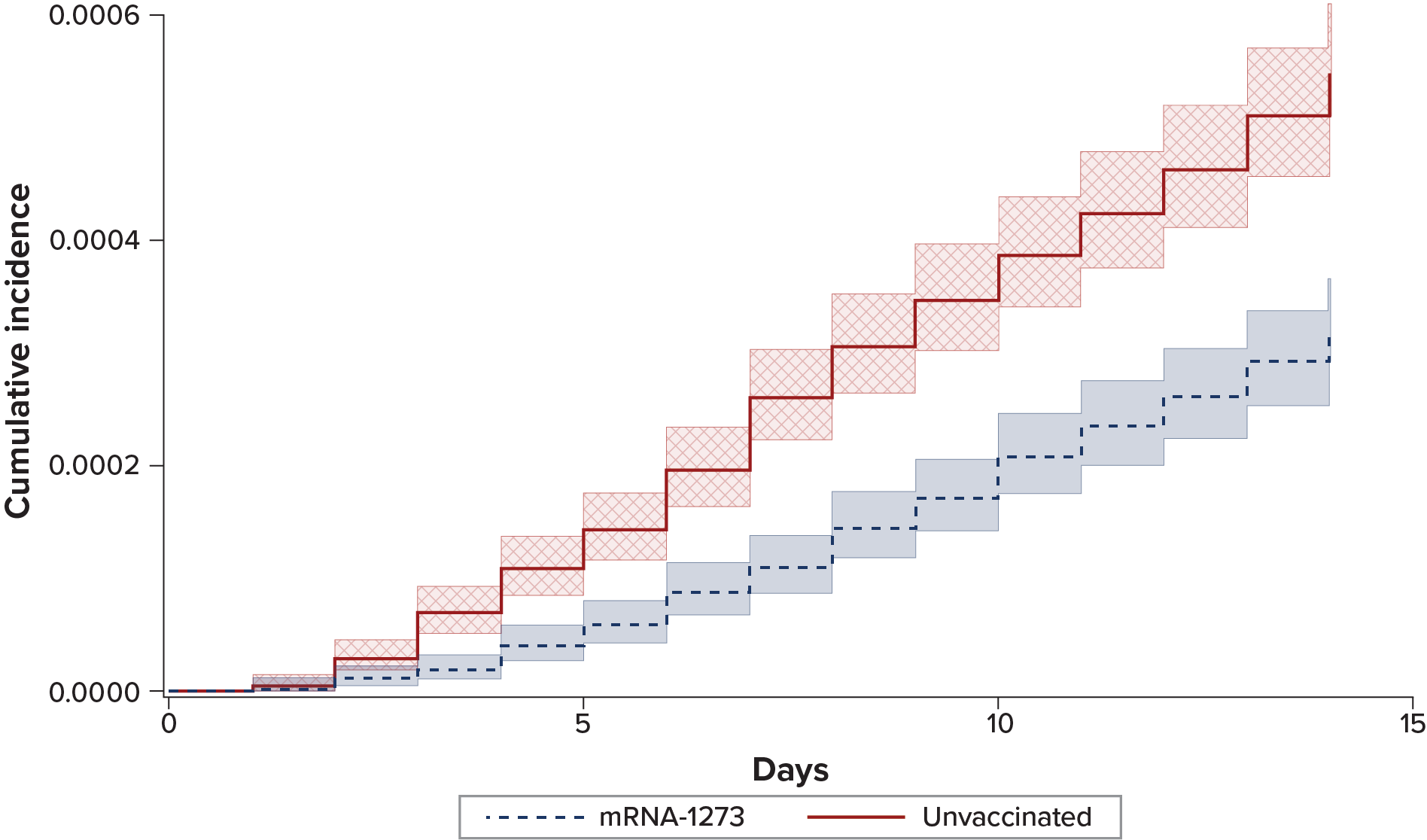


F. JNJ-7836735 vs. Unvaccinated Comparators, CVS Health

i. Medically Diagnosed COVID-19


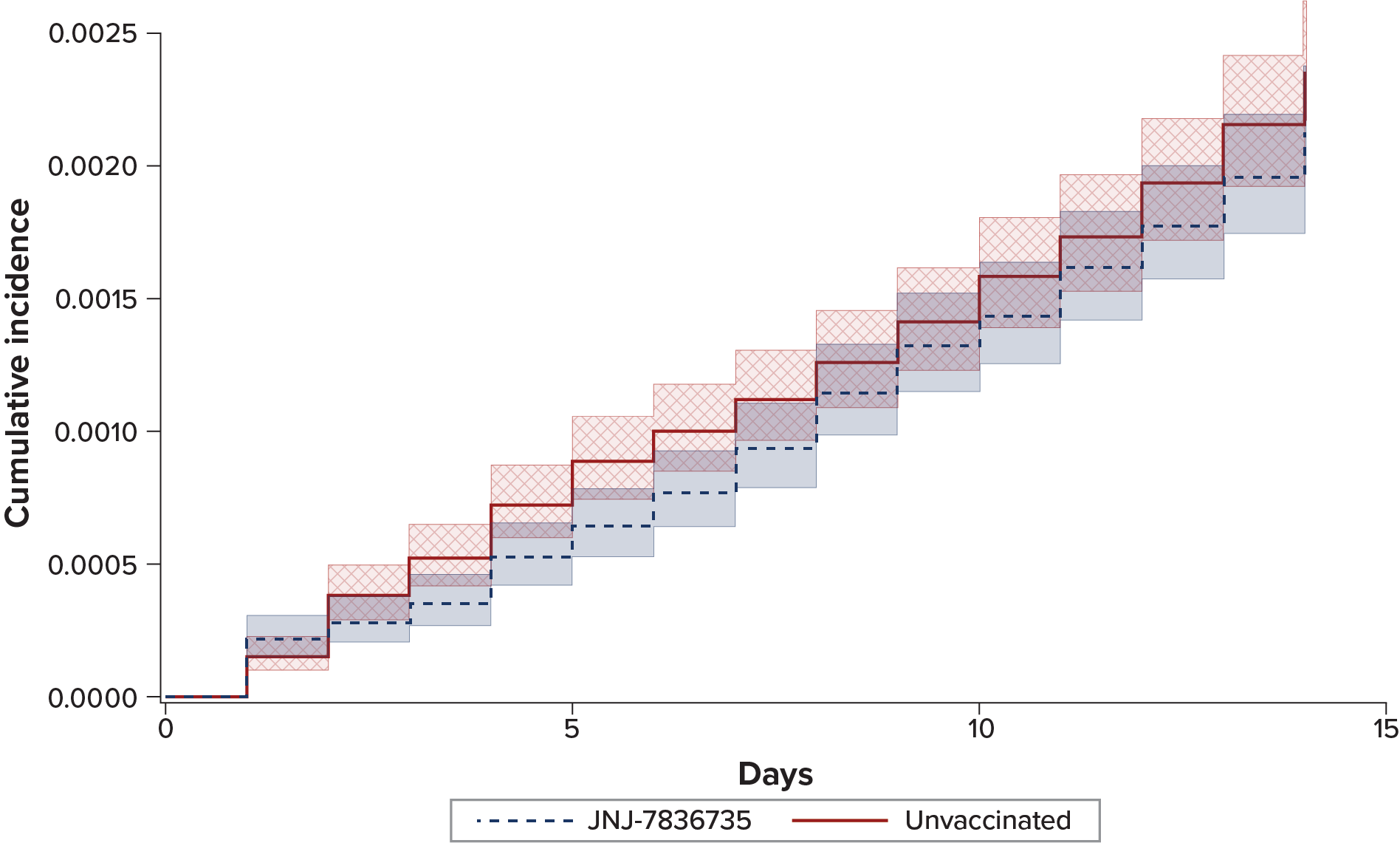


ii. Hospital/ED-Diagnosed COVID-19


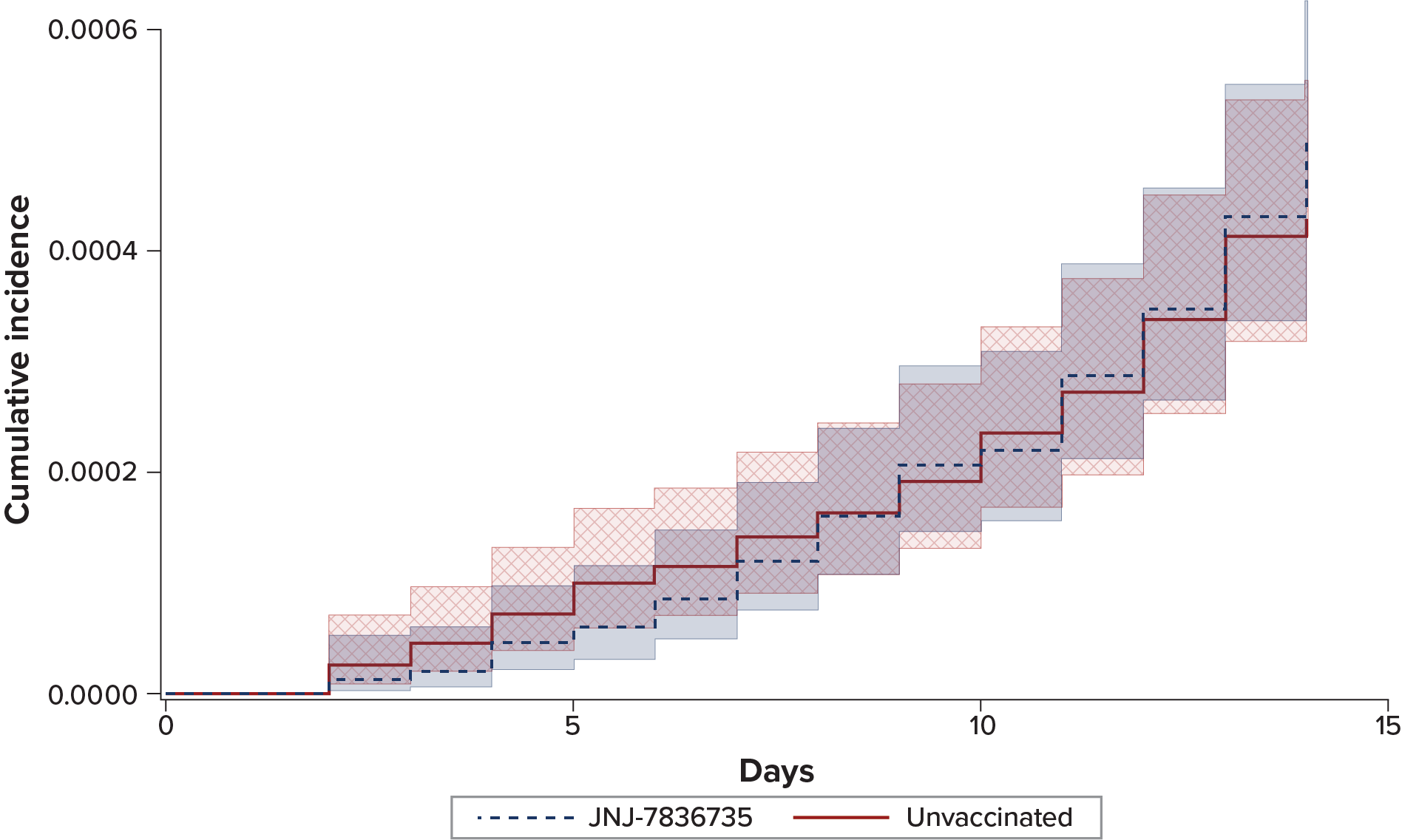


CI = confidence interval; COVID-19 = coronavirus disease 2019; ED = emergency department.

Note: The study Time 0 (the date of vaccination or matched comparator date) is the first day of follow-up, and it is displayed as day 1 in the cumulative incidence figures.

1. Estimated Effectiveness of Receiving a Complete Primary Series of COVID-19 Vaccine in Adults Aged 18-64 Years, Compared With Being Unvaccinated, by SARS-CoV-2 Variant Era

**
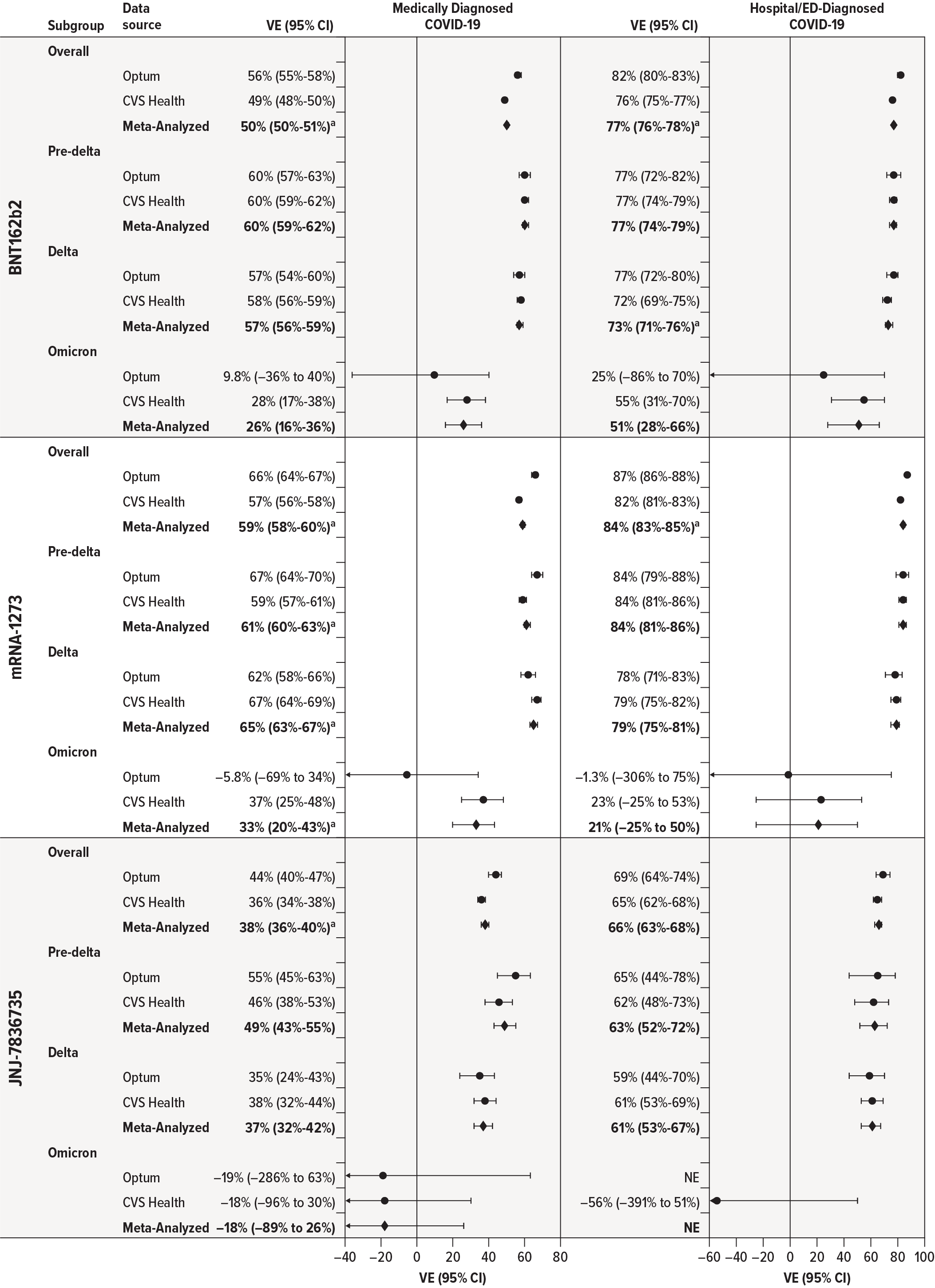
**

CI = confidence interval; COVID-19 = coronavirus disease 2019; ED = emergency department; NC = not calculable; VE = vaccine effectiveness.

^a^ Indicates evidence of statistical heterogeneity between data source–specific estimates, *p* < 0.05.

1. Estimated Relative Effectiveness of Receiving a Complete Primary Series of COVID-19 Vaccine in Adults Aged 18-64 Years, Compared With Receiving a Complete Primary Series of Different COVID-19 Vaccines


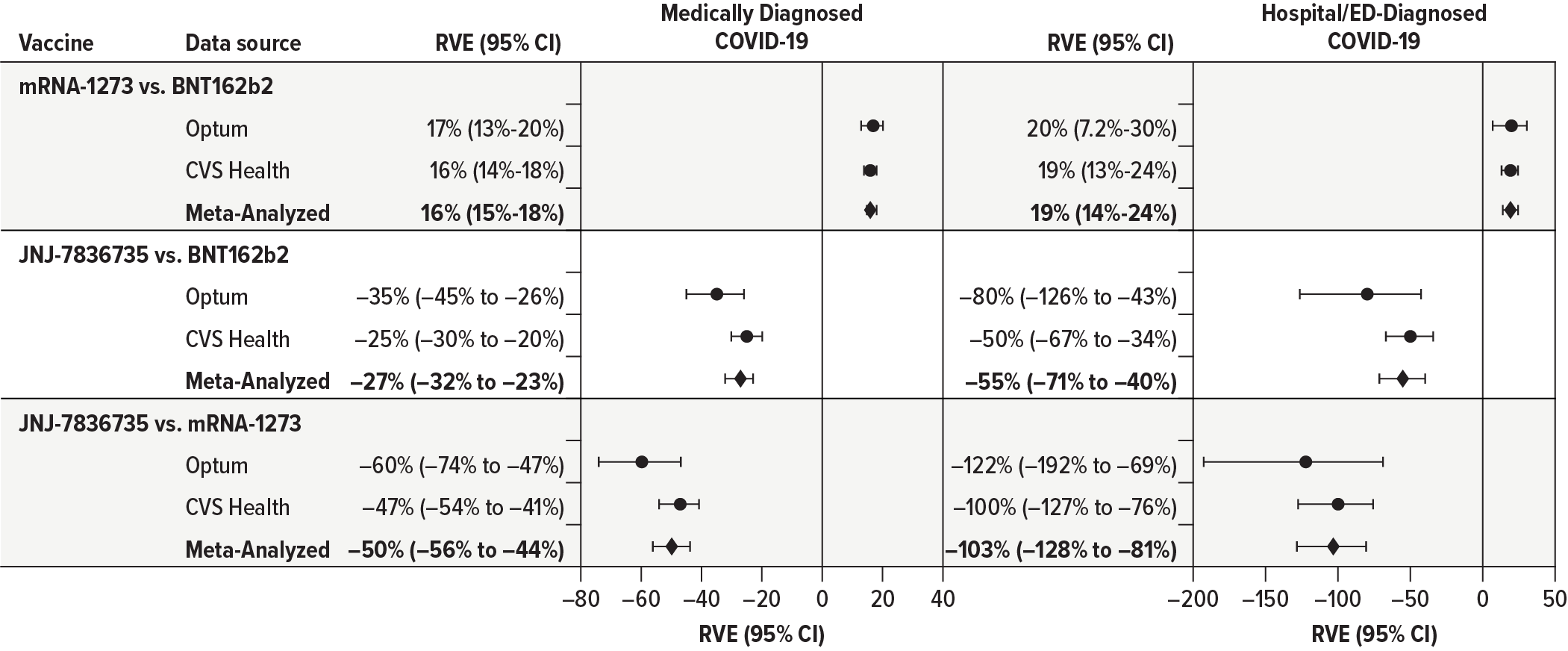


CI = confidence interval; ED = emergency department; RVE = relative vaccine effectiveness.

Note: For the comparison of a complete mRNA-1273 primary series versus complete BNT162b2 primary series, the study period was 18 Dec 2020 through the end of study. For the comparison of a complete JNJ-7836735 primary series versus complete BNT162b2 primary series, the study period was 27 Feb 2021 through the end of study. For the comparison of a complete JNJ-7836735 primary series versus complete mRNA-1273 primary series, the study period was 27 Feb 2021 through the end of study.

1. Estimated Effectiveness of Receiving a Single Dose of a 2-Dose Primary Series of COVID-19 Vaccine in Adults Aged 18‑64 Years, Compared With Being Unvaccinated


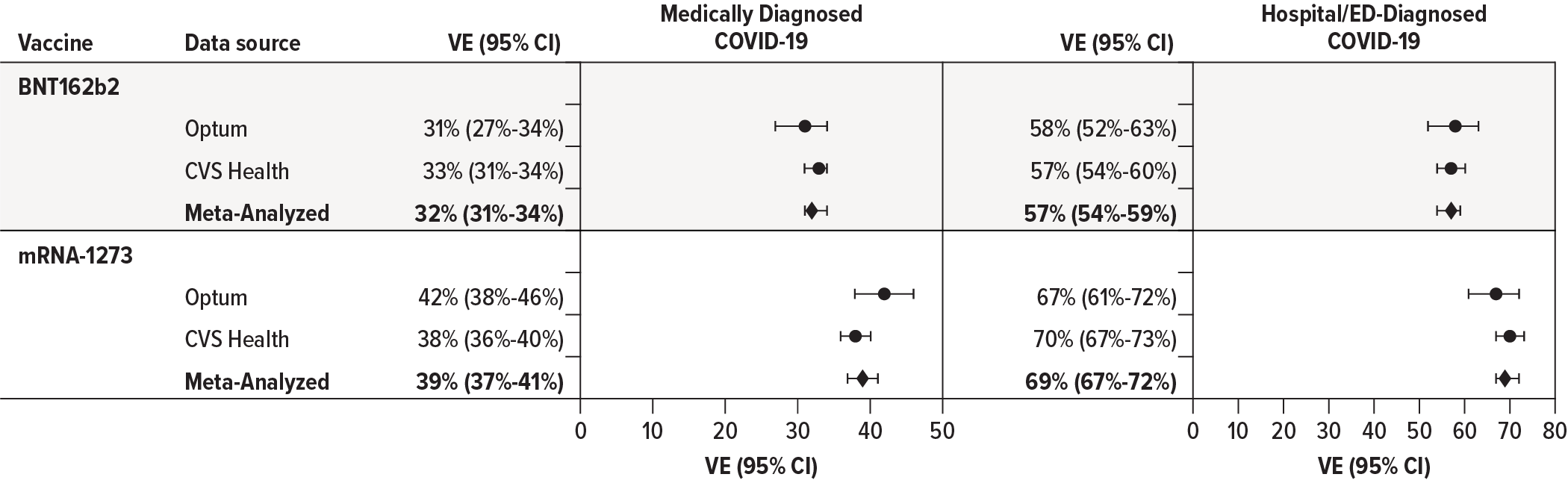


COVD-19 = coronavirus disease 2019; CI = confidence interval; ED = emergency department; VE = vaccine effectiveness.

1. Weighted Cumulative Incidence of COVID‑19 Outcomes in Adults Aged 18-64 Years Receiving a Single Dose of a 2-Dose Primary Series of COVID-19 Vaccine and Matched Unvaccinated Comparators

A. 1 Dose of BNT162b2 vs. Unvaccinated Comparators, Optum

i. Medically Diagnosed COVID-19


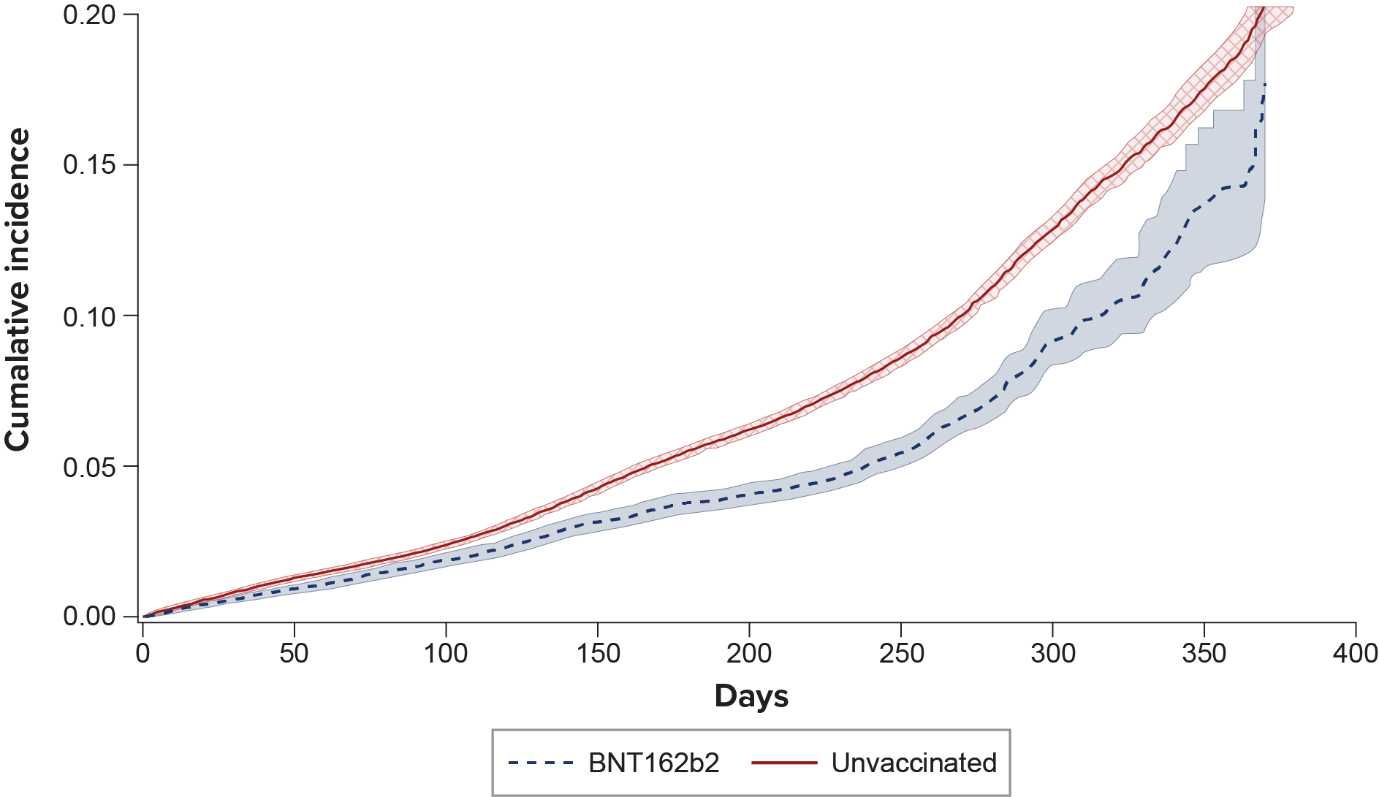


ii. Hospital/ED-Diagnosed COVID-19


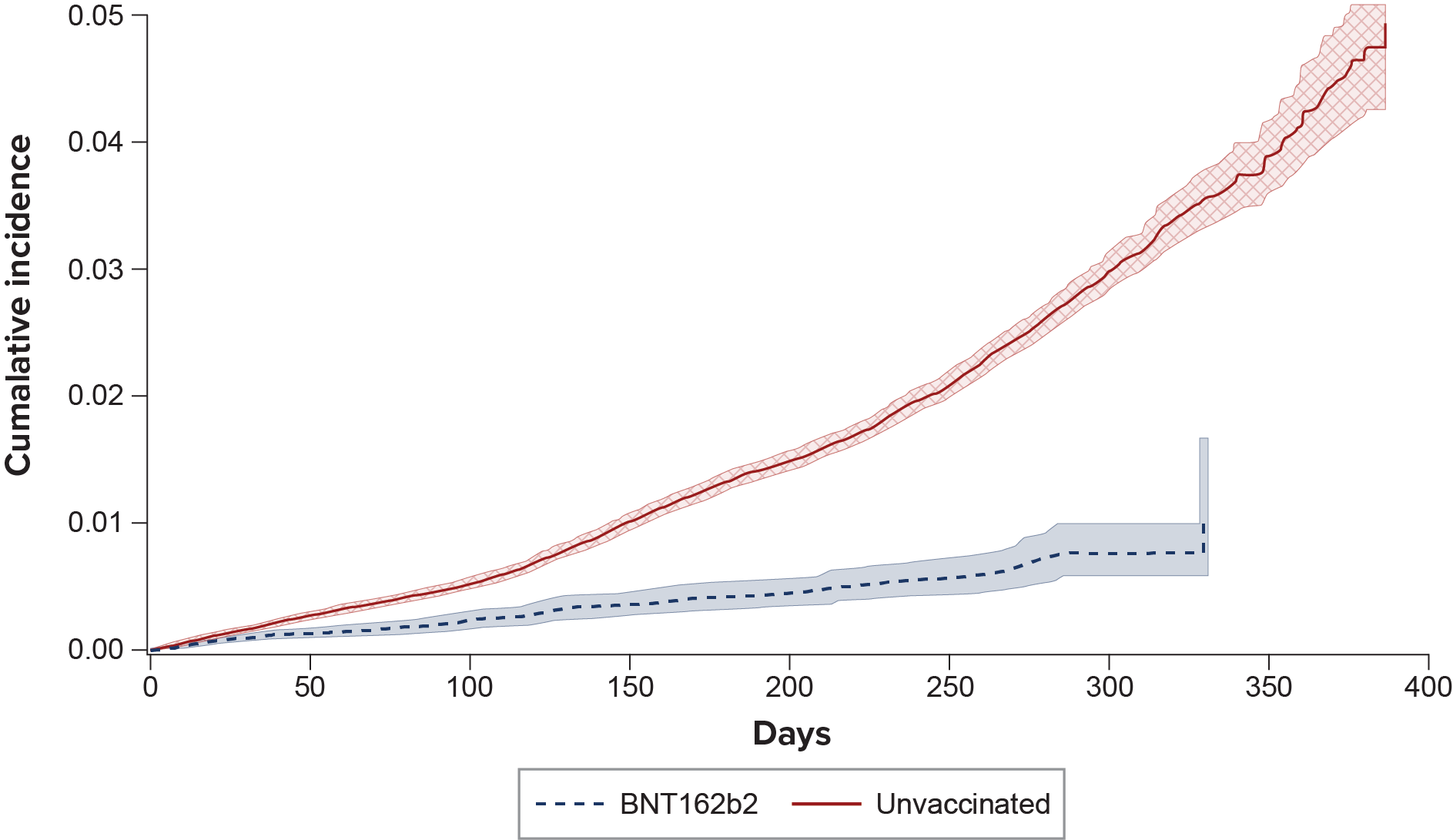


B. 1 Dose of mRNA-1273 vs. Unvaccinated Comparators, Optum

i. Medically Diagnosed COVID-19


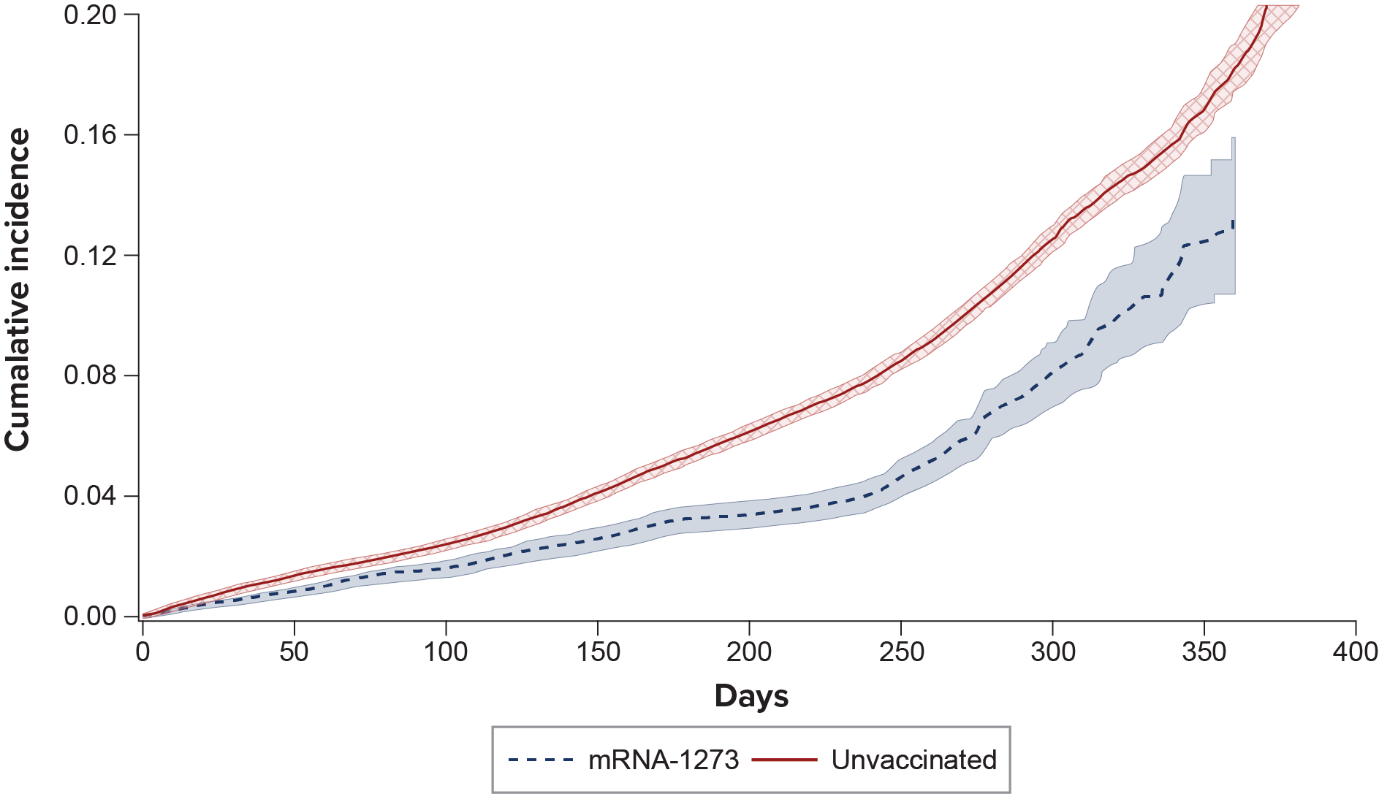


ii. Hospital/ED-Diagnosed COVID-19


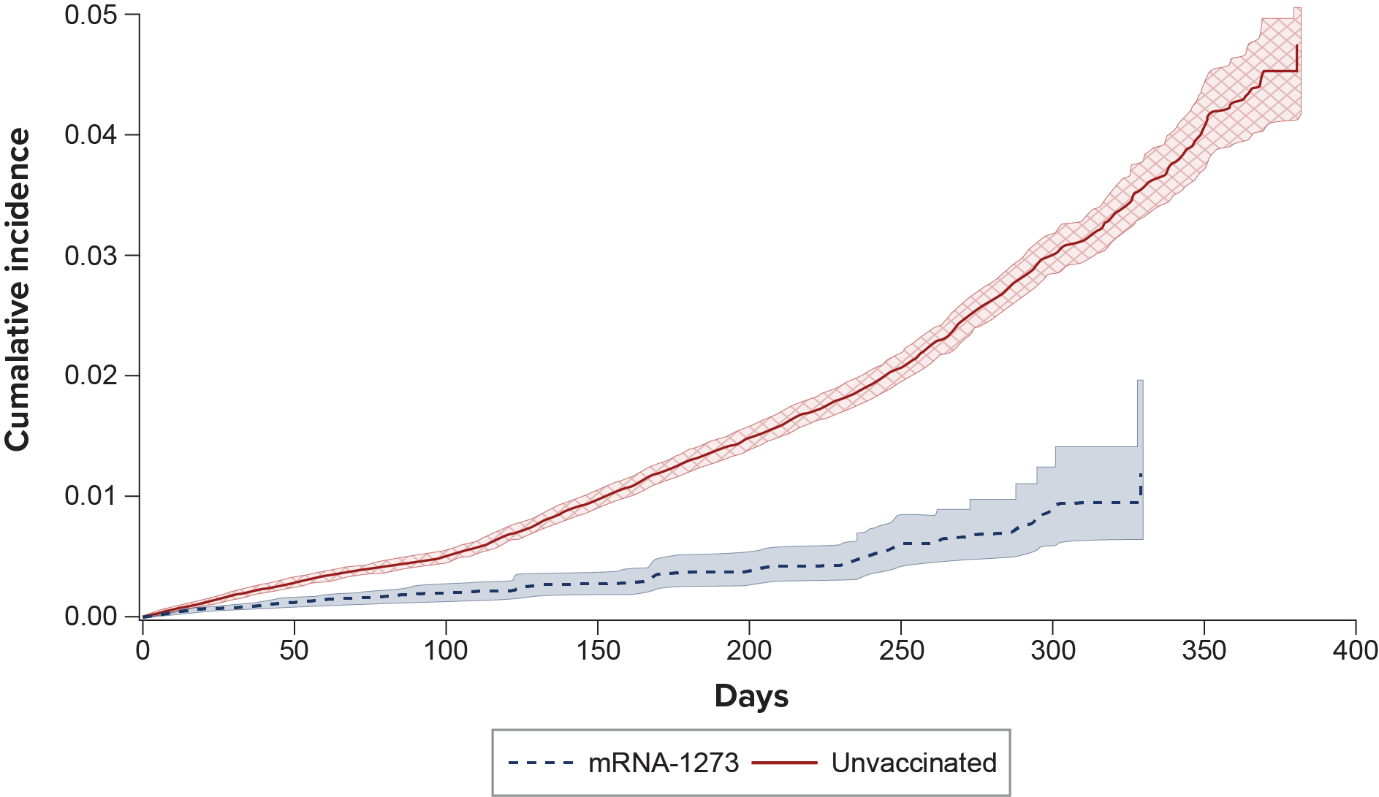


C. 1 Dose of BNT162b2 vs. Unvaccinated Comparators, CVS Health

i. Medically Diagnosed COVID-19


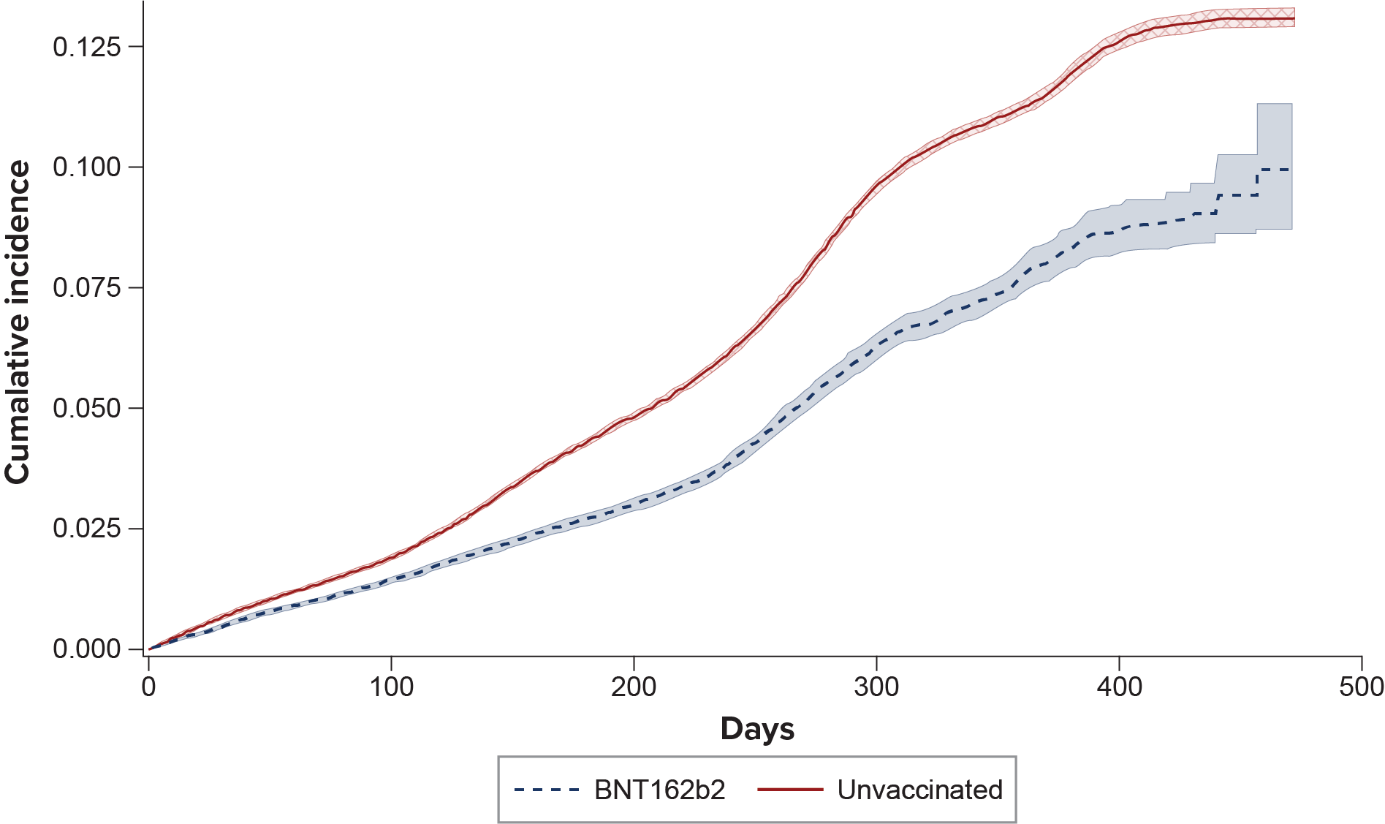


ii. Hospital/ED-Diagnosed COVID-19


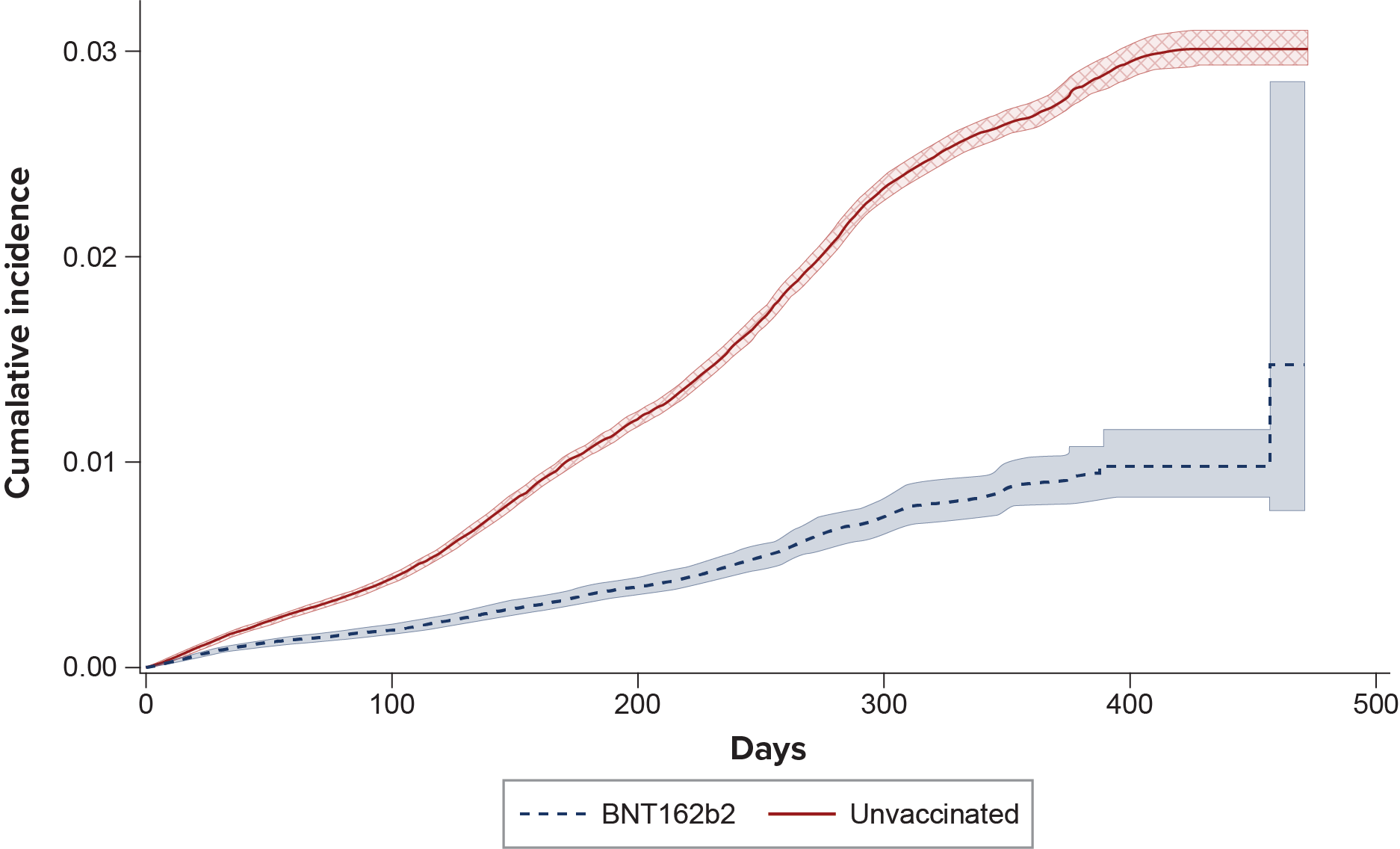


D. 1 Dose of mRNA-1273 vs. Unvaccinated Comparators, CVS Health

i. Medically Diagnosed COVID-19


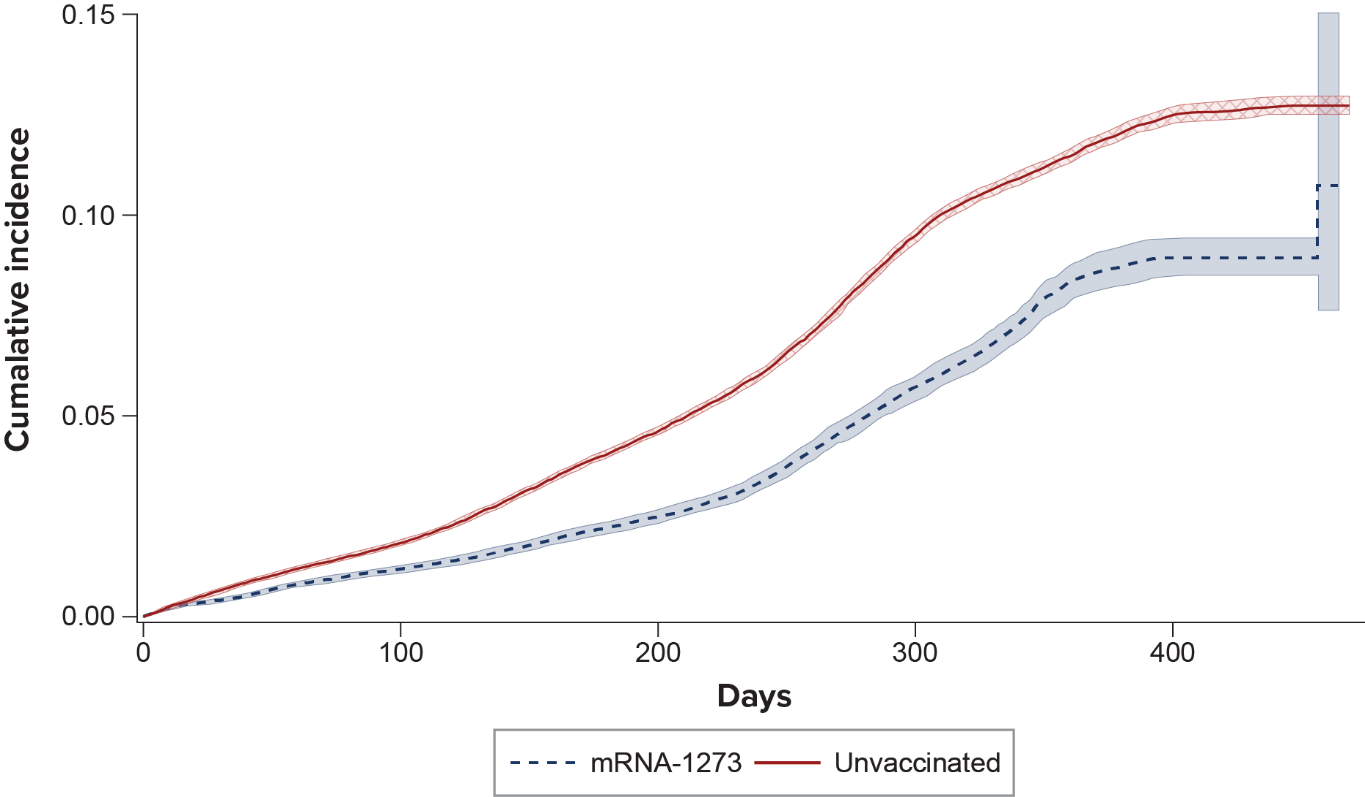


ii. Hospital/ED-Diagnosed COVID-19


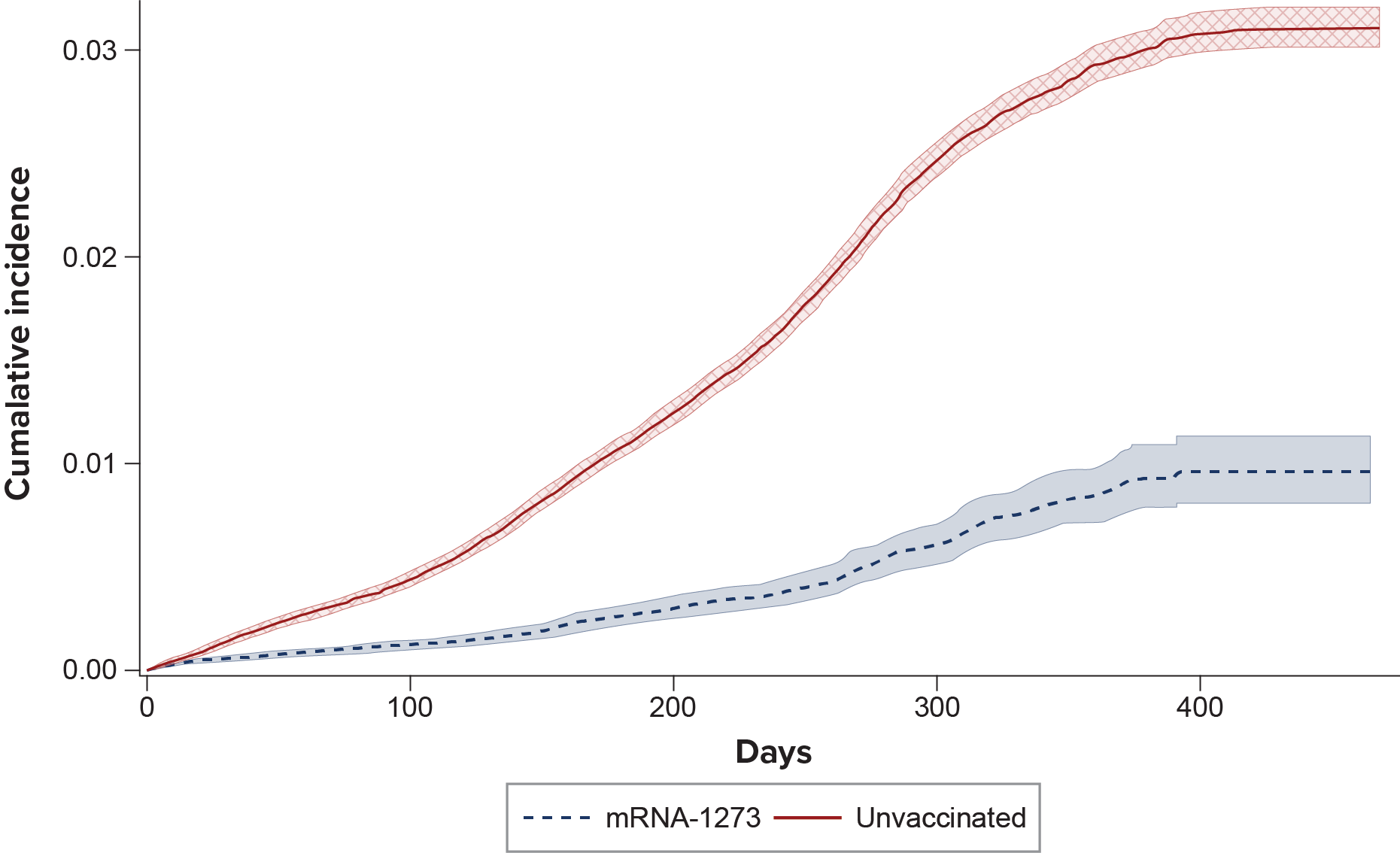


COVID-19 = coronavirus disease 2019.
